# Fluorinated Arylethynyl Substituents in Zn(II) Complexes of Azadipyrromethene Guide Self-Assembly in Crystals

**DOI:** 10.1021/acs.inorgchem.6c03689

**Published:** 2026-10-15

**Authors:** David A. Raufman, Neha Jadhav, Shashika A. Arachchige, Lexi R. Knight, Jayvic C. Jimenez, Geneviève Sauvé

**Affiliations:** † Department of Chemistry, 2546 Case Western Reserve University, 10900 Euclid Avenue, Cleveland, Ohio 44106, United States; ‡ Lawrence Livermore National Laboratory, Livermore, California 94550, United States

## Abstract

Zn­(II) complexes of 2,8-arylethynyl 1,3,7,9-tetraphenyl azadipyrromethenes are promising molecular semiconductors with strong visible-NIR absorption and a nonplanar distorted tetrahedral geometry around the Zn­(II) center, but their charge transport properties are limited by the orientation and strength of intermolecular π–π stacking interactions. To optimize their intermolecular π–π stacking interactions, we explored substitution of the pyrrolic phenylethynyl groups in Zn­(L1)_2_ at the phenyl *meta* positions with either fluorines (Zn­(L1-*m*-2F)_2_) or trifluoromethyls (Zn­(L1-*m*-2CF_3_)_2_). Fluorination had minimal effects on optical properties and slightly deepened frontier orbital energy levels. On the other hand, fluorination strongly affected the complexes’ molecular packing: while Zn­(L1-*m*-2F)_2_ had the same *C*2/*c* space group as Zn­(L1)_2_, the −CF_3_ groups in Zn­(L1-*m*-2CF_3_)_2_ promoted a higher ordered space group of *Fddd*. Further, in Zn­(L1)_2_ and Zn­(L1-*m*-2F)_2_, the phenylethynyl groups stack with the core phenyls of neighboring molecules, whereas in Zn­(L1-*m*-2CF_3_)_2_, the bis­(trifluoromethyl)-substituted phenylethynyl groups of neighboring molecules stack with each other with a shorter π–π stacking distance than in the other two molecules. Computations revealed that fluorination decreases the internal reorganization energy for electron transfer and increases overlap integrals for charge transfer. Fluorination is thus a promising strategy to tune molecular packing and parameters relevant to charge transport.

## Graphical abstract

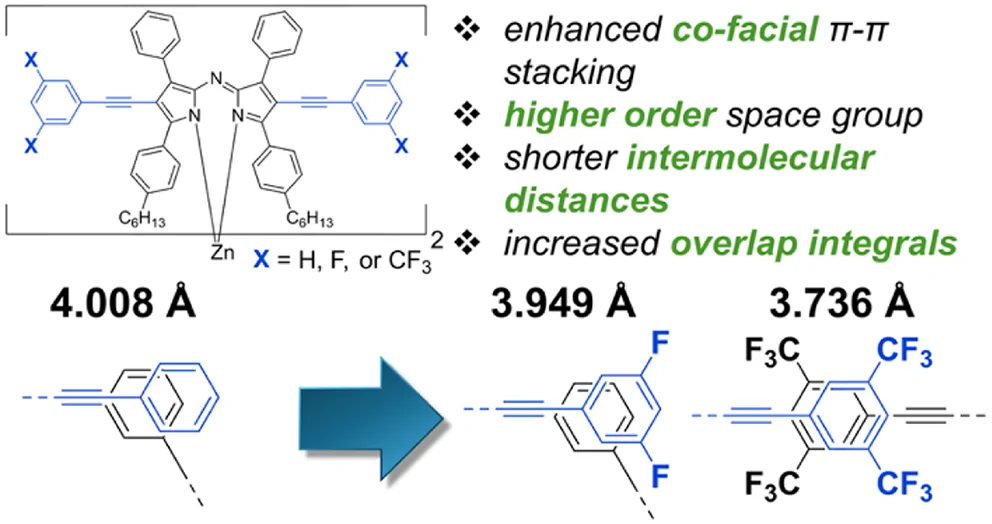

## Introduction

Molecular semiconductors have attracted much attention for their use in a variety of electronic applications, such as organic photovoltaics, field effect transistors, and light-emitting diodes.^1–5^ The performance of organic semiconductors is governed not only by their electronic structure but also by their solid-state structure and film morphology, which makes it a priority to control their molecular packing.^6^ Planar π-conjugated molecules often exhibit strong intermolecular interactions that result in large crystallites. Such well-ordered packing is advantageous for optimizing charge carrier mobility, but charge transport is often limited to one or two dimensions, a problem if the charge transport direction is not well aligned with the application or if the film has defects or cracks. Further, the strong π–π stacking of planar molecular semiconductors often leads to overaggregation, which lowers solubility and can lead to unfavorable phase separation when blended with polymers, limiting their application.^7^ Nonplanar π-conjugated molecules, on the other hand, incorporate 3-dimensional structures that disrupt planarity, thus tuning down the propensity for overaggregation.^8,9^ In addition, the 3-dimensional structures can support isotropic charge transport, enabling new applications.^10,11^ The combined ability to transport charges in multiple directions and decreased tendency to overaggregate are highly desirable for photovoltaic applications.^12^

A well-known example of a nonplanar π-conjugated semiconductor used in organic photovoltaics is fullerene and its derivatives, such as [6,6] phenyl-C61-butyric acid methyl ester (PCBM; Figure 1). The spherical shape facilitates isotropic charge transport, making it easier to extract electrons in blends with conjugated polymers. However, fullerene derivatives have weak visible/NIR absorption, poor energy level tunability, high costs, a tendency to aggregate in blends over time, and poor scalability.^13^ These shortcomings have motivated the search for alternative nonfullerene acceptors (NFAs).^14^ Initial work with planar molecular NFAs led to low power conversion efficiencies due to unfavorable blend morphology with π-conjugated polymer donors. Specifically, larger planar molecules tend to overaggregate and phase-separate on a large scale from the conjugated polymers. Efficiencies increased dramatically when researchers turned down this overaggregation by either adding bulky side chains, as in ITIC,^13^ or by connecting two or more planar molecular semiconductors together with flexible or rotatable linkers to create nonplanar molecules, as in Bis-PDI-T-EG and SBF-4DPPDCV.^15,16^ A less-explored approach to achieving nonplanarity in π-conjugated molecules is through metal coordination. For example, boron subphthalocyanines such as Cl-BsubPc have bowl-like structures, whereas homoleptic M­(II) complexes of azadipyrromethenes (ADPs) such as Zn­(L2)_2_ have a distorted tetrahedral structure.^17–19^ Both were used successfully as NFAs in organic photovoltaics, demonstrating this approach’s potential for the development of nonplanar molecular semiconductors.

**Figure 1:**
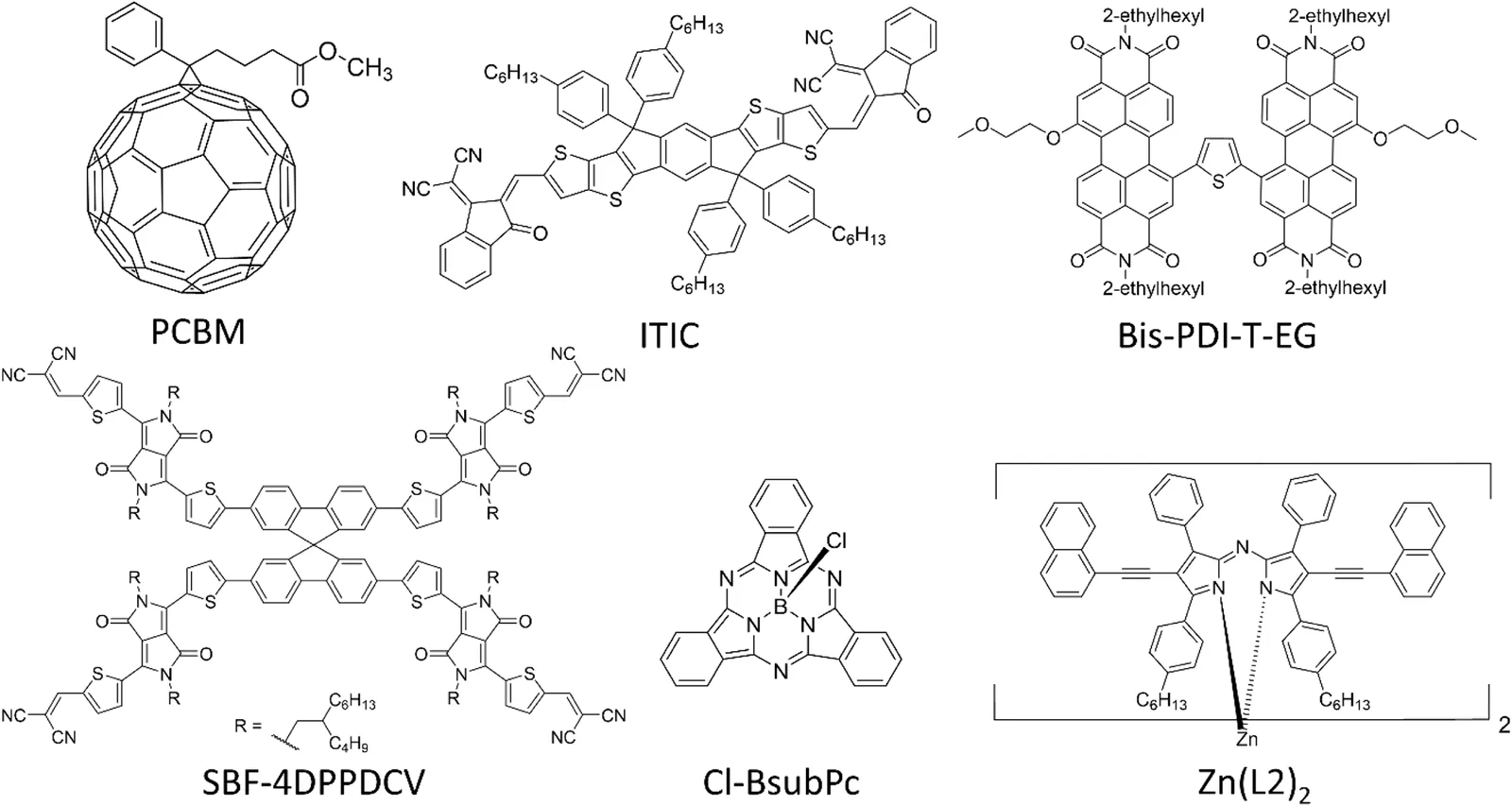
Nonplanar π-conjugated molecular semiconductors mentioned in the text.

Azadipyrromethenes (ADPs) are promising ligands for the development of nonplanar molecular semiconductors.^8,20^ They are bidentate ligands with strong absorption in the visible-NIR region, high electron affinity, good chemical tunability, and low synthetic complexity, especially for the 1,3,7,9-tetraphenyl-ADP derivatives. ADPs are best known when coordinated with difluoroboryl (*aza*-BODIPY) but can also be coordinated with metals. While difluoroboryl coordination results in a planar π-conjugated system, M­(II) complexes of ADP have a nonplanar distorted tetrahedral geometry.^21,22^ We have demonstrated that homoleptic Zn­(II) complexes of ADP are promising NFAs, particularly when arylethynyl groups are installed at the pyrrolic positions.^19,23^ These arylethynyl groups extend π-conjugation, red-shift the absorption, increase electron affinity, and improve device performance in solar cells. Using the closed-shell Zn­(II) metal as opposed to Co­(II) or Ni­(II) is key to ensure a stable excited state required for charge transfer.^24^ In these complexes, Zn­(II) holds the two ligands in place with four intramolecular π–π interactions between the two ligands, and light absorption is ligand-centered. Notably, Zn­(L2)_2_ showed isotropic charge transport and achieved a relatively high power conversion efficiency (PCE) of 5.5% when blended with the polymer donor poly­(3-hexylthiophene) (P3HT).^19,25^ However, its electron mobility was low at 2 × 10^–5^ cm^2^ V^–1^ s^–1^, limiting its performance. Structure–property studies have shown that homoleptic complexes of ADP derivatives can achieve charge carrier mobilities as high as 10^–3^ cm^2^ V^–1^ s^–1^,^26,27^ which is high for mobilities measured by Space Charge Limited Current (SCLC) in diodes, motivating further investigation.

Charge transport in most molecular semiconductors occurs via a hopping mechanism, which can be described as electron transfer from one molecule to a neighboring molecule. Thus, at the microscopic level, intrinsic charge carrier mobility depends on the rate of charge transfer between molecules following the Marcus theory.^28^ To maximize the rate of charge transfer, one must minimize the internal reorganization energy (λ) and maximize the charge transfer integral (or electronic coupling, V). The λ is the energy necessary for molecular structural rearrangement during charge transfer. A small reorganization energy (<0.1 eV) suggests minimal changes in molecular geometry and is associated with high charge carrier mobility and charge separation.^29–32^ DFT calculations have shown that Zn­(II) complexes of ADP derivatives tend to have very small internal reorganization energy for both hole (λ^+^) and electron (λ^–^) transfer due to their large rigid π-conjugated system.^33^ The charge transfer integral reflects the interaction strength between two molecules; higher V values are associated with higher intrinsic mobility.^34^ The transfer integral is very sensitive to the relative position of the two molecules^35^ and is usually estimated using pairs from the crystal packing. Computing V for all pairs helps identify conducting pathways in the crystal.^36^ Nonzero calculated V in oligoacenes with high charge carrier mobility ranges between 8 and 85 meV, depending on the pair and the oligoacene.^35^

Intermolecular π–π stacking interactions are often described as either sandwich, T-shaped, or parallel-displaced.^37,38^ In both sandwich and parallel-displaced types, the aryl groups are stacked parallel to each other, or cofacial (CF), and the difference has to do with whether the aryl groups are perfectly aligned face-to-face (sandwich) or shifted (parallel-displaced). For T-shaped, the edge of one aryl group points toward the face of another. It is generally thought that CF π–π interactions lead to higher mobilities than T-shape due to larger overlap integrals, and there is evidence that CF π–π stacking interactions are especially advantageous for electron transport.^39–41^ One way to encourage CF π–π stacking is using heteroatoms such as fluorines.^42^ Fluorine is especially good for promoting stronger intermolecular interactions due to its small size and very high electron affinity. In addition to the fluoro group (−F), the trifluoromethyl group (−CF_3_) is also of interest because it has a higher Hammett constant than the −F group (σ_m_(CF_3_) = 0.43 vs σ_m_(F) = 0.34, for *meta* substitution), indicating that −CF_3_ has a higher electron-withdrawing character than −F.^43^ Additionally, Sun et al. have shown computationally that trifluoromethylation of benzene stabilizes the cofacial orientation more effectively than fluorination.^40^ The −CF_3_ group also introduces larger steric hindrance compared to the −F group, which could influence molecular packing behavior.

Monofluorination of the naphthylethynyl groups in Zn­(L2)_2_ was found to promote cofacial π–π stacking and increase electron mobility by an order of magnitude.^26^ However, fluorinated naphthalenes are less accessible than fluorinated benzenes. Monofluorination of phenylethynyl groups of Zn­(WS3)_2_ with either −F or −CF_3_ at the *para* position also led to high electron mobility, but the type of the π–π stacking interactions could not be confirmed due to the lack of a crystal structure.^44^ It is thus advantageous to increase the crystallization tendency, which improves the likelihood of obtaining a crystal structure. Interestingly, adding hexyl groups on the proximal phenyls of Zn­(WS3)_2_, see Zn­(L1)_2_ in Figure 2, increases crystallization—the crystal structure of Zn­(L1)_2_ has been published.^19^ To further increase the tendency to crystallize and guide molecular packing, this work investigates introducing two (rather than one) fluorinated groups on each phenylethynyl of Zn­(L1)_2_. To ensure the dipole moment is in the same direction as our previously studied *para*-substituted complex, we chose to place the fluorinated substituents at the *meta* positions: *meta*-difluoro substitution, Zn­(L1-*m*-2F)_2_ and *meta*-bis­(trifluoromethyl), Zn­(L1-m-2CF_3_)_2_ shown in Figure 2. All complexes were successfully synthesized and formed crystals suitable for X-ray diffraction, enabling us to better understand the effect of fluorination on molecular packing and on parameters relevant to charge transport.

**Figure 2:**
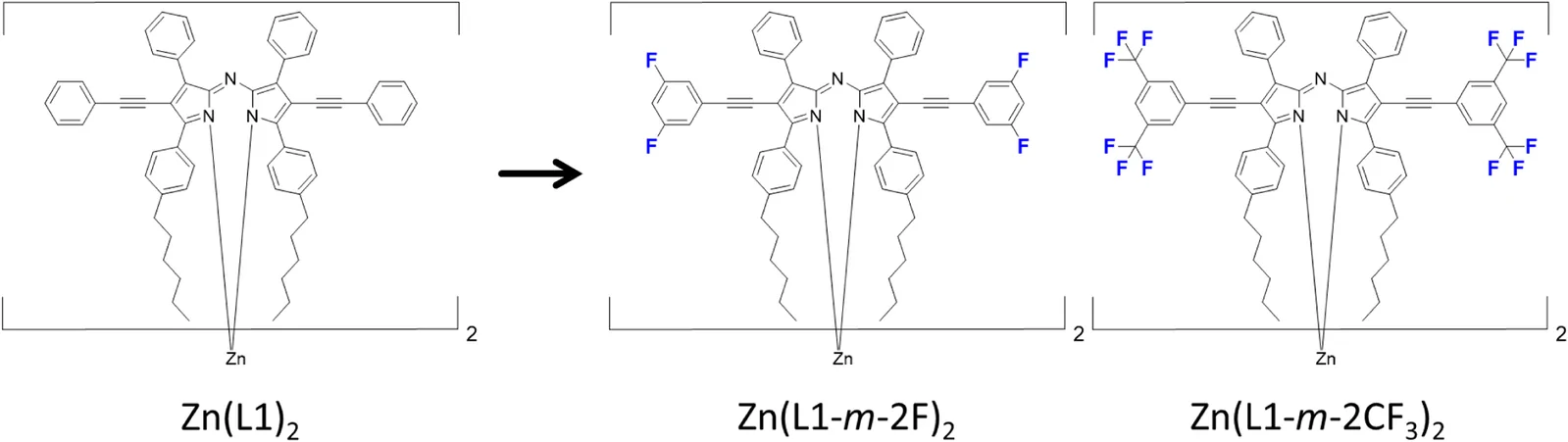
Chemical structures of the molecules investigated.

## Results and Discussion

### Synthesis

The synthesis of the fluorinated ADPs is shown in Scheme 1. First, an azadipyrromethene derivative with iodine at the pyrrolic positions (ADPI_2_) was synthesized according to the literature.^19^ In step (a), ADPI_2_ was reacted with tributyl­(phenylethynyl)tin derivatives via Stille coupling, yielding ligands functionalized with phenylethynyl (L1), 3,5-difluorophenylethynyl (L1-*m*-2F), or 3,5-bis­(trifluoromethyl)­phenylethynyl (L1-*m*-2CF_3_). The ligands were then chelated with Zn­(II) (step b) by reacting the ligand with NaH followed by ZnCl_2_ at room temperature. The Zn­(II)-chelated molecules were purified via liquid–liquid diffusion recrystallization in THF/MeOH for 4 days. The Zn­(II)-chelated ADPs generally required only one recrystallization to achieve acceptable purity. The identity and purity of the Zn­(II) complexes were confirmed by ^1^H NMR spectroscopy, MALDI-TOF MS, and elemental analysis; see the SI. Fluorination was confirmed by ^19^F NMR, observing the triplet signal near −109 ppm for aromatic fluorine in L1-*m*-2F and a broad singlet near −63 ppm for trifluoromethyl groups in L1-*m*-2CF_3_.

**Scheme 1:**
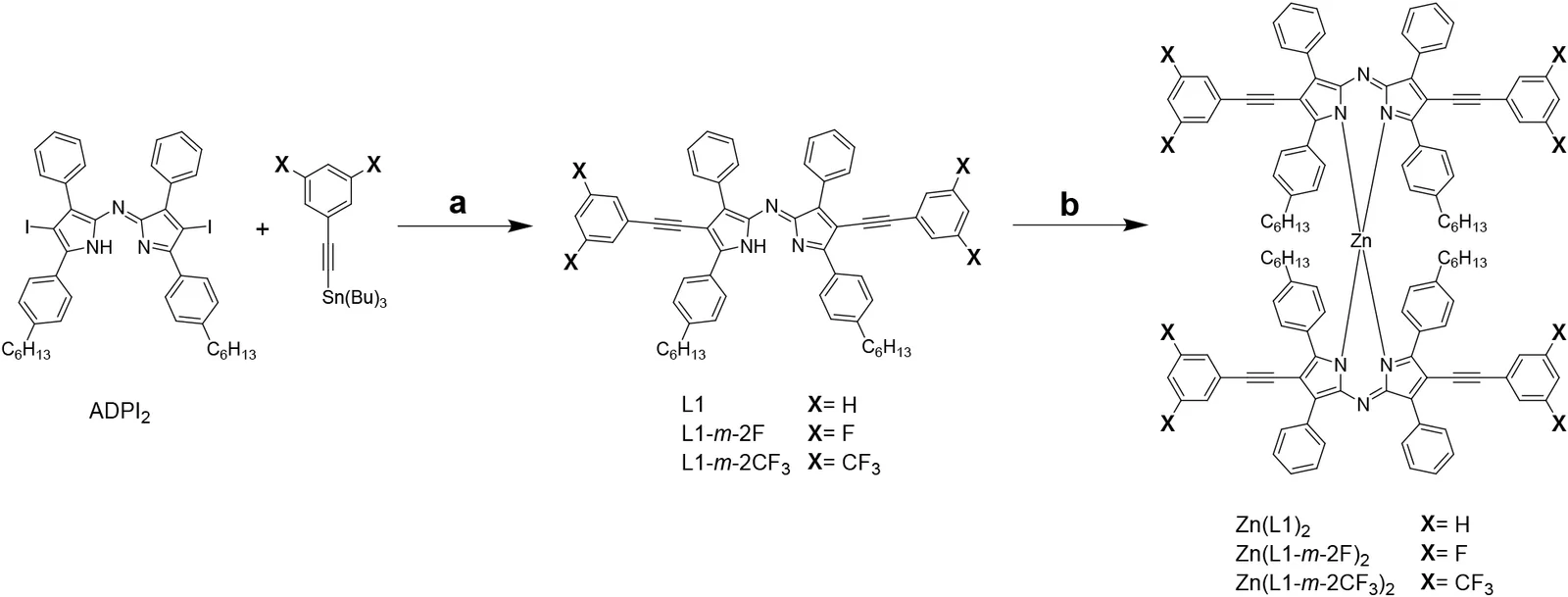
Synthesis of the Zn­(II) Complexes: a) Pd­(PPh_3_)_4_ in Xylene at 130 °C for 1 h, Then Tributyl­(phenylethynyl)­tin Derivatives at 130 °C for 16 h; b) NaH in Tetrahydrofuran at R.T. for 4 h, Then ZnCl_2_ at R.T. for 16 h

### Thermal Properties

The thermal properties were evaluated by thermogravimetric analysis (TGA) and differential scanning calorimetry (DSC), and the results are summarized in Figure 3 and Table 1. TGA shows that all compounds are thermally stable with decomposition temperatures at 5% weight loss (T_d5%_) ranging from 434 to 454 °C. These decomposition temperatures are sufficiently high for device applications. DSC revealed one endothermic peak in the first heating cycle and no peaks during the first cooling cycle and subsequent heating and cooling cycles. This is consistent with the samples melting and then forming an amorphous solid upon cooling at 10 °C/min, typical for ADP-based complexes.^26^ To confirm that the endothermic peaks are melting transitions, we measured the melting point temperatures using a melting point apparatus (Meltemp, values not reported), and they were like the endothermic peak temperatures. Fluorination slightly increases the melting points from 212 °C for Zn­(L1)_2_ to 225 and 219 °C for Zn­(L1-*m*-2F)_2_ and Zn­(L1-*m*-2CF_3_)_2_, respectively. Of the three complexes, Zn­(L1-*m*-2F)_2_ showed the highest melting point, suggesting that this complex has the strongest intermolecular interactions in crystals. ΔH_fusion_ for the Zn­(II) complexes did not significantly change with fluorination, with 43 J/g for Zn­(L1)_2_, 47 J/g for Zn­(L1-*m*-2F)_2_, and 42 J/g for Zn­(L1-*m*-2CF_3_)_2_. Since ΔH_fusion_ strongly depends on the degree of crystallinity in the solid sample, and considering the complexes were recrystallized under similar conditions, we conclude that each of the Zn­(II) complexes has a similar tendency to crystallize.

**Figure 3:**
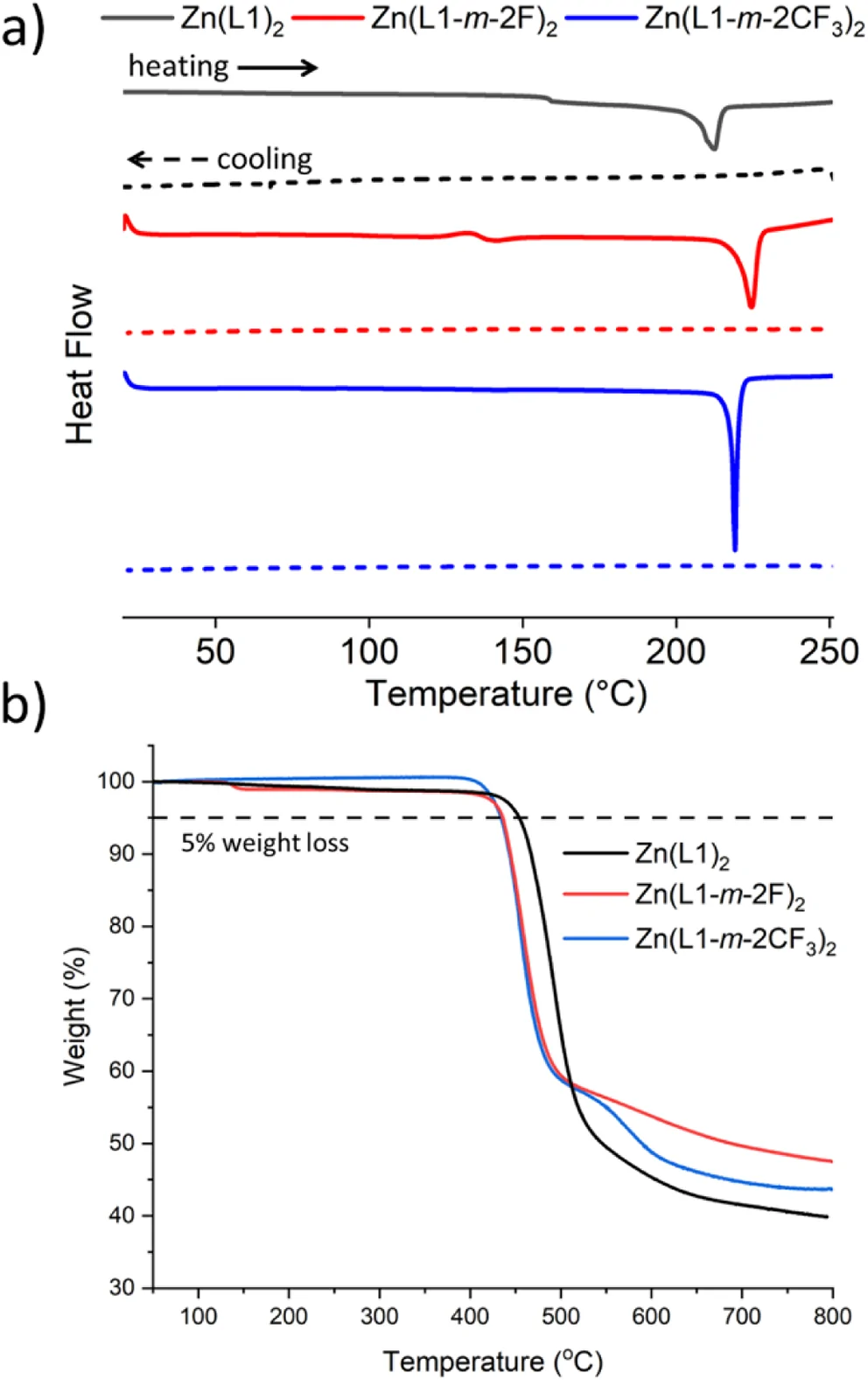
a) DSC thermograms of complexes on the first heating and cooling cycles. b) TGA plot of complexes, showing 5% weight loss (T_d5%_).

**Table 1:** Summary of Thermal Properties Obtained by TGA and DSC

Complex	T_d5%_ (°C)	T_m_ (°C)	ΔH_fusion_ (J/g)
Zn(L1)_2_	454	212	43
Zn(L1-*m*-2F)_2_	436	225	47
Zn(L1-*m*-2CF_3_)_2_	434	219	42

### Optical Properties

The UV–vis absorption spectra of the Zn­(II) complexes in chloroform solution and in as-cast films are shown in Figure 4 and the optical properties are summarized in Table 2. In solution, all complexes exhibit strong absorption in the visible-NIR region, with high molar absorptivity (ε) on the order of 10^4^–10^5^ M^–1^ cm^–1^. The maximum ε decreased from 158,000 M^–1^ cm^–1^ for Zn­(L1)_2_ to 127,000 M^–1^ cm^–1^ for Zn­(L1-*m*-2CF_3_)_2_, though that is still very high and adequate for a light absorber. The absorption λ_max_ slightly blue shifts with fluorination: λ_max_ for Zn­(L1)_2_ is longest at 679 nm, followed by Zn­(L1-*m*-2F)_2_ (668 nm) and then Zn­(L1-*m*-2CF_3_)_2_ (663 nm).

**Figure 4:**
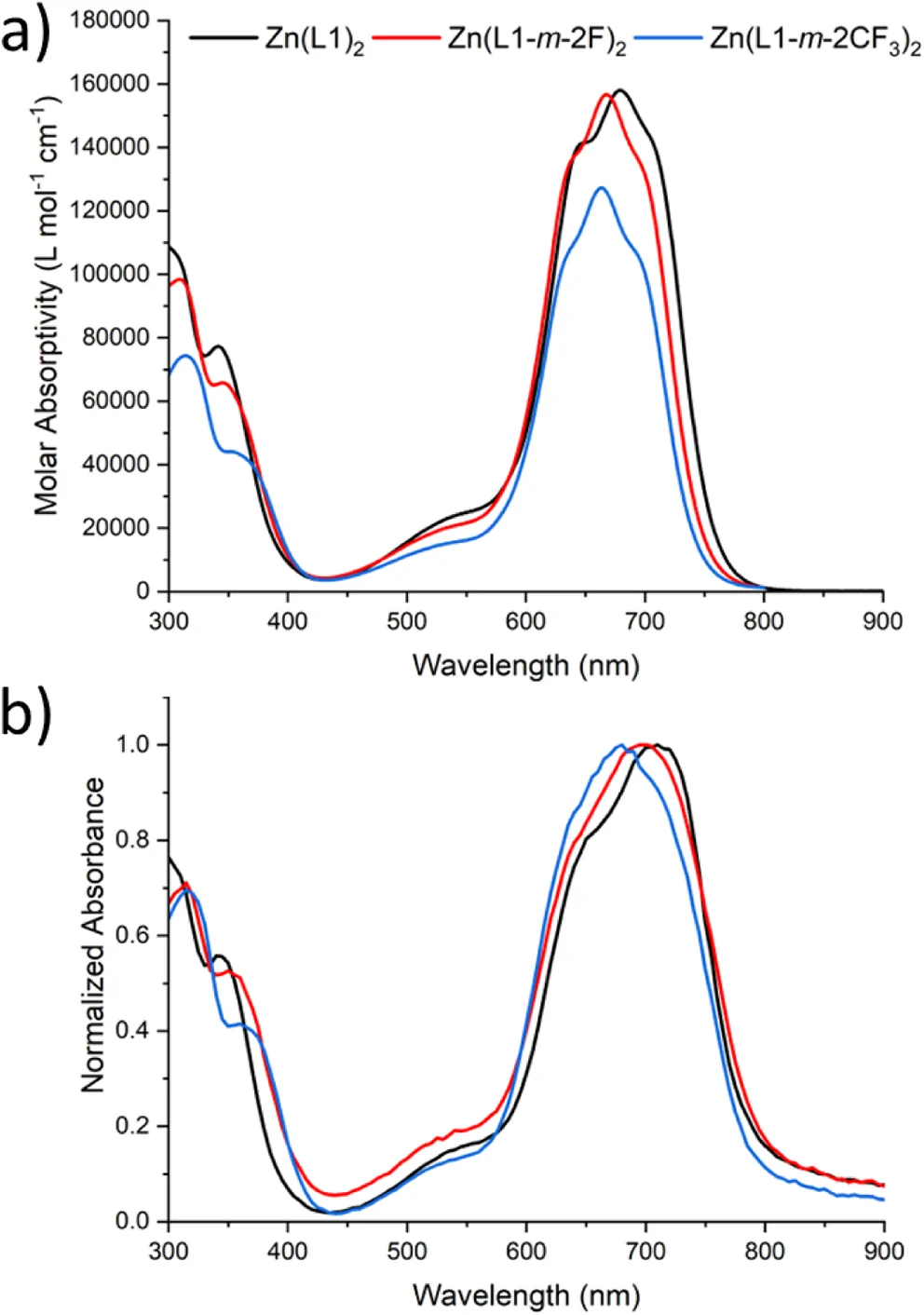
UV–vis spectra of the Zn­(II) complexes in a) CHCl_3_ solutions and b) as-cast films cast from CHCl_3_ solutions.

**Table 2:** Summary of Optical Properties of CHCl_3_ Solutions and As-Cast Films

	**Solution**	**As-cast film**			
Complex	λ_max_ (nm) (ε, 10^3^ M^–1^ cm^–1^)	λ_max_ (as-cast, nm)	λ_max_ red-shift (nm)^a^	λ_onset_ (as-cast, nm)	Optical gap (eV)
**Zn(L1)**_**2**_	300 (109), 342 (77), 650 (142), 679 (158)	710	31	787	1.58
**Zn(L1-** *m* **-2F)**_**2**_	310 (98), 346 (66), 668 (157)	695	27	801	1.55
**Zn(L1-** *m* **-2CF**_**3**_)_**2**_	314 (74), 356 (44), 663 (127)	680	17	790	1.57

a Red shift of main absorption band λ_max_ upon film formation.

To investigate the optical properties of the complexes in films, we dissolved the complexes in chloroform and spin-coated them onto ZnO-coated glass slides. The main absorption band of all Zn­(II) complexes red-shifted upon film formation. The as-cast film absorption of Zn­(L1)_2_ shifted from 679 to 710 nm (a 31 nm red shift from solution), Zn­(L1-*m*-2F)_2_ shifted from 668 to 695 nm (27 nm red shift), and Zn­(L1-*m*-2CF_3_)_2_ shifted from 663 to 680 nm (17 nm red shift). The red shift upon film formation is consistent with increased intermolecular interactions in films. The magnitude of the red shift decreases with fluorination, from 31 nm in Zn­(L1)_2_ to 27 and 17 nm in Zn­(L1-*m*-2F)_2_ and Zn­(L1-*m*-2CF_3_)_2_, respectively. The optical gaps of the complexes were estimated from the onset of the main absorption band and slightly decreased with fluorination. The complexes did not show any measurable emission, whether in solution or in the solid state.

After the as-cast film absorption spectra were obtained, the slides were annealed at 150 °C for 10 min. Thermal annealing was performed to allow the molecules to reorient into a more thermodynamically favorable morphology. The absorption spectra of the annealed films are shown in Figure 5 and summarized in Table 3. Upon annealing, the λ_max_ of Zn­(L1)_2_, Zn­(L1-*m*-2F)_2_, and Zn­(L1-*m*-2CF_3_)_2_ red-shifted by 5, 35, and 25 nm, respectively. In addition, the λ_onset_ of Zn­(L1)_2_ red-shifted by 18 nm upon annealing, whereas Zn­(L1-m-2F)_2_ did not have a significant shift and Zn­(L1-*m*-2CF_3_)_2_ exhibited a blue-shift of 22 nm upon annealing. The absorption onsets were used to estimate the optical gaps. The optical gaps increase with fluorination from 1.54 eV for the nonfluorinated Zn­(L1)_2_ to 1.55 and 1.62 eV for Zn­(L1-*m*-2F)_2_ and Zn­(L1-*m*-2CF_3_)_2_, respectively.

**Figure 5:**
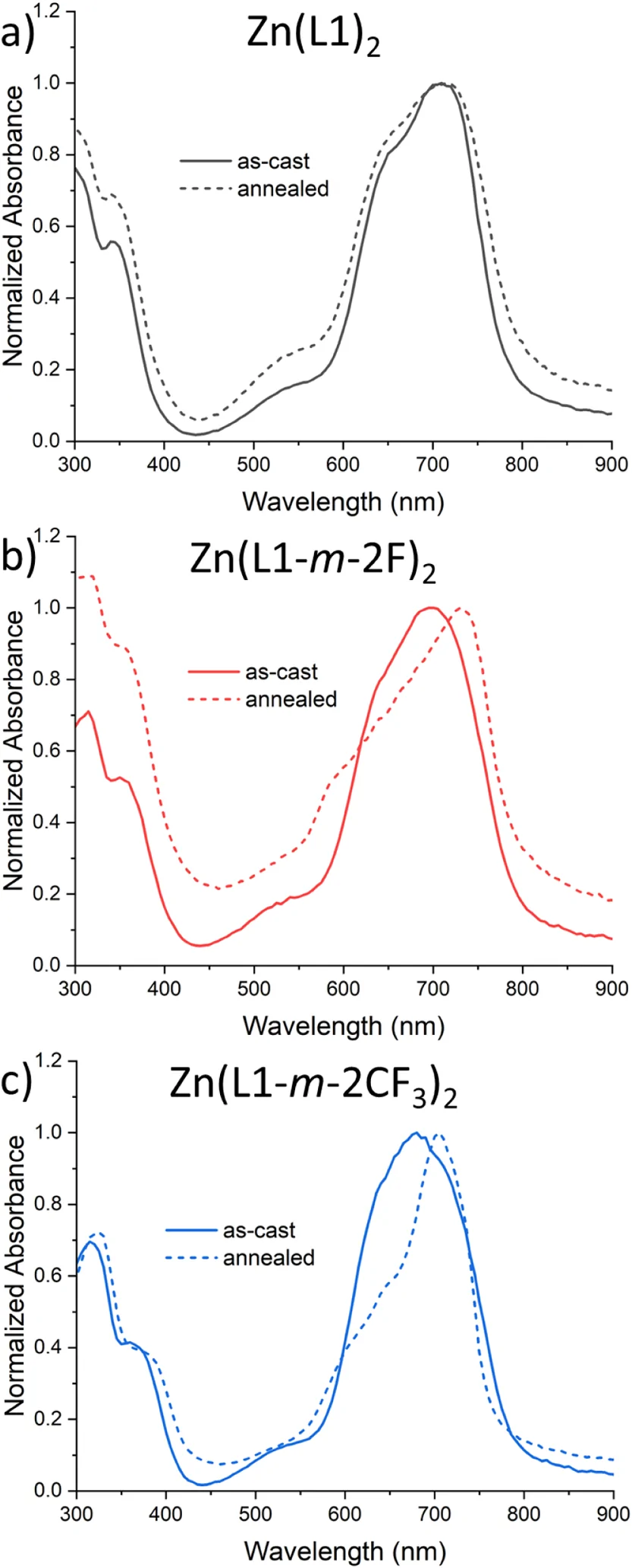
UV–vis absorption spectra of as-cast (solid line) and annealed (dashed line) films of a) Zn­(L1)_2_, b) Zn­(L1-*m*-2F)_2_, and c) Zn­(L1-*m*-2CF_3_)_2_.

**Table 3:** Summary of Optical Properties of Annealed Films

Complex	λ_max_ (nm)	λ_max_ shift (nm)^a^	λ_onset_ (nm)	λ_onset_ shift (nm)^b^	Optical gap (eV)
**Zn(L1)**_**2**_	715	5	807	20	1.54
**Zn(L1-** *m* **-2F)**_**2**_	730	35	800	–1	1.55
**Zn(L1-** *m* **-2CF**_**3**_)_**2**_	705	25	768	–22	1.62

a Shift in λ_max_ from as-cast to annealed films.

b Shift in λ_onset_ from as-cast to annealed films.

### Electrochemical Properties

The cyclic voltammograms of the Zn­(II) complexes in dichloromethane are shown in Figure 6 and the electrochemical properties are summarized in Table 4. Each Zn­(II) complex shows two consecutive reversible oxidation processes and two consecutive reversible reduction processes. Both the E_1/2,ox_ and E_1/2,red_ values anodically shift upon fluorination, indicating that fluorination makes the complexes harder to oxidize and easier to reduce. The E_1/2,ox_ value for the first oxidation event shifts from 0.42 V for Zn­(L1)_2_ to 0.54 and 0.59 V for Zn­(L1-*m*-2F)_2_ and Zn­(L1-*m*-2CF_3_)_2_, respectively. This corresponds to an anodic shift of 0.12 V with −F groups and 0.17 V with −CF_3_ groups. The greater anodic shift observed for −CF_3_ groups is consistent with its stronger electron-withdrawing strength. The E_1/2,red_ values for the first reduction event shift from −1.28 V for Zn­(L1)_2_ to −1.21 V and −1.17 V for Zn­(L1-*m*-2F)_2_ and Zn­(L1-*m*-2CF_3_)_2_, respectively. This corresponds to shifts of 0.07 and 0.11 V for fluorination with −F and −CF_3_ groups, respectively. The effect of fluorination is thus smaller on the reduction event than that on the oxidation event.

**Figure 6:**
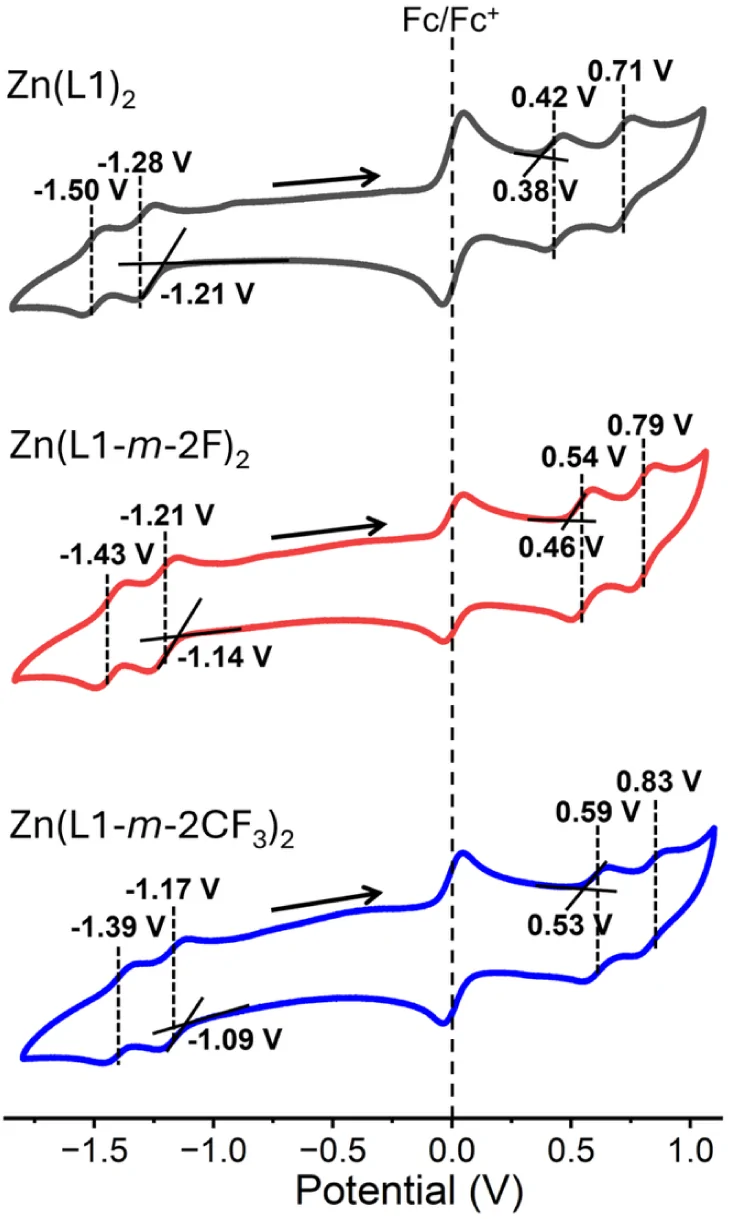
Cyclic voltammograms of Zn­(L1)_2_, Zn­(L1-*m*-2F)_2_, and Zn­(L1-*m*-2CF_3_)_2_ in DCM solutions with Fc/Fc^+^ as the internal standard.

**Table 4:** Electrochemical Properties of Zn­(L1)_2_, Zn­(L1-*m*-2F)_2_, and Zn­(L1-*m*-2CF_3_)_2_ in DCM Solutions

Complex	E_1/2,ox_ (V)	E_1/2,red_ (V)	E_onset, ox_ (V)	E_onset, red_ (V)	IP/EA (eV)	E_gap_ (eV)
Zn(L1)_2_	0.42, 0.71	–1.28, −1.50	0.38	–1.21	5.18/3.59	1.59
Zn(L1-*m*-2F)_2_	0.54, 0.79	–1.21, −1.43	0.46	–1.14	5.26/3.66	1.60
Zn(L1-*m*-2CF_3_)_2_	0.59, 0.83	–1.17, −1.39	0.53	–1.09	5.33/3.71	1.62

The ionization potential (IP) and electron affinity (EA) energies were estimated from the onsets of the first oxidation and reduction peaks, respectively. The negative values of the IP and EA are often used to estimate the highest occupied molecular orbital (HOMO) and lowest unoccupied molecular orbital (LUMO) energy levels, respectively.^45^ As expected, the IP and EA slightly increase with −F and −CF_3_ groups. These modest increases are similar to what we have observed in the past for fluorination of these types of complexes, despite having two fluorinated groups on the phenylethynyls rather than one.^26^ Fluorination brings the IP higher than the air stability threshold of 5.2 eV,^46^ suggesting that the fluorinated complexes should be air-stable for hole transport. The IP increases slightly more than the EA with fluorination, resulting in a small increase in the energy gap (E_gap_) from 1.59 eV for Zn­(L1)_2_ to 1.60 and 1.62 eV for Zn­(L1-*m*-2F)_2_ and Zn­(L1-*m*-2CF_3_)_2_, respectively.

### DFT Calculations

To further investigate the electronic properties of the Zn­(II) complexes, density functional theory (DFT) calculations were carried out on Zn­(L1)_2_, Zn­(L1-*m*-2F)_2_, and Zn­(L1-*m*-2CF_3_)_2_, with the hexyl groups replaced with methyl groups to reduce computational cost. The impact of fluorine substitution on the electronic structure was evaluated through analysis of the Kohn–Sham frontier orbital energies, as well as IP and EA. The computed properties are summarized in Table 5, while the contour plots of HOMO and LUMO are shown in Figure S26. The HOMO and LUMO contour plots indicate that fluorination does not significantly alter orbital distributions, with the frontier orbitals remaining predominantly localized on the ADP core. Accordingly, fluorination at the *meta* positions is not expected to have a significant impact on the HOMO and LUMO energy levels, as was observed for similar complexes that had one fluorinated substituent at the *para* position.^47^ The HOMO energy levels slightly decrease upon fluorination, going from −4.71 eV in Zn­(L1)_2_ to −4.86 eV and −4.87 eV for Zn­(L1-*m*-2F)_2_ and Zn­(L1-*m*-2CF_3_)_2_, respectively. The LUMO energies decreased from −3.57 eV in Zn­(L1)_2_ to −3.72 eV in Zn­(L1-*m*-2F)_2_ and −3.73 eV in Zn­(L1-*m*-2CF_3_)_2_. These results demonstrate that fluorination stabilizes both the HOMO and LUMO, though the difference between using −F and −CF_3_ is negligible, despite −CF_3_ being a stronger electron-withdrawing group. The calculated IP and EA both increase by 0.15–0.16 eV with the addition of −F and by 0.17 eV with the addition of −CF_3_. The calculated fundamental gap (E_gap_), defined as the difference between IP and EA, is similar for all three compounds, at about 1.58 eV.

**Table 5:** Calculated Ground-State Properties

Complex	HOMO (eV)	LUMO (eV)	IP (eV)	EA (eV)	E_gap_ (eV)
Zn(L1)_2_	–4.71	–3.57	4.92	3.34	1.58
Zn(L1-*m*-2F)_2_	–4.86	–3.72	5.08	3.49	1.59
Zn(L1-*m*-2CF_3_)_2_	–4.87	–3.73	5.09	3.51	1.58

### Single-Crystal X-ray Diffraction

The Zn­(II) chelates were crystallized by slow evaporation of THF over 4 days, yielding large crystals suitable for single-crystal X-ray crystallography. Capped stick structures of the Zn­(II) complexes are shown in Figure 7, and the results are summarized in Table 6. The published crystal structure of Zn­(L1)_2_ is included for comparison.^19^ The −F and −CF_3_ groups (colored in yellow) are located at the expected positions in the molecules. Furthermore, the Zn­(II) complexes have the expected distorted tetrahedral geometry for M­(II) complexes of ADP, with Zn­(L1-*m*-2CF_3_) having a slightly larger dihedral angle of 75.19° compared to 73.19° in Zn­(L1-*m*-2F)_2_ and 71.31° in Zn­(L1)_2_.

**Figure 7:**
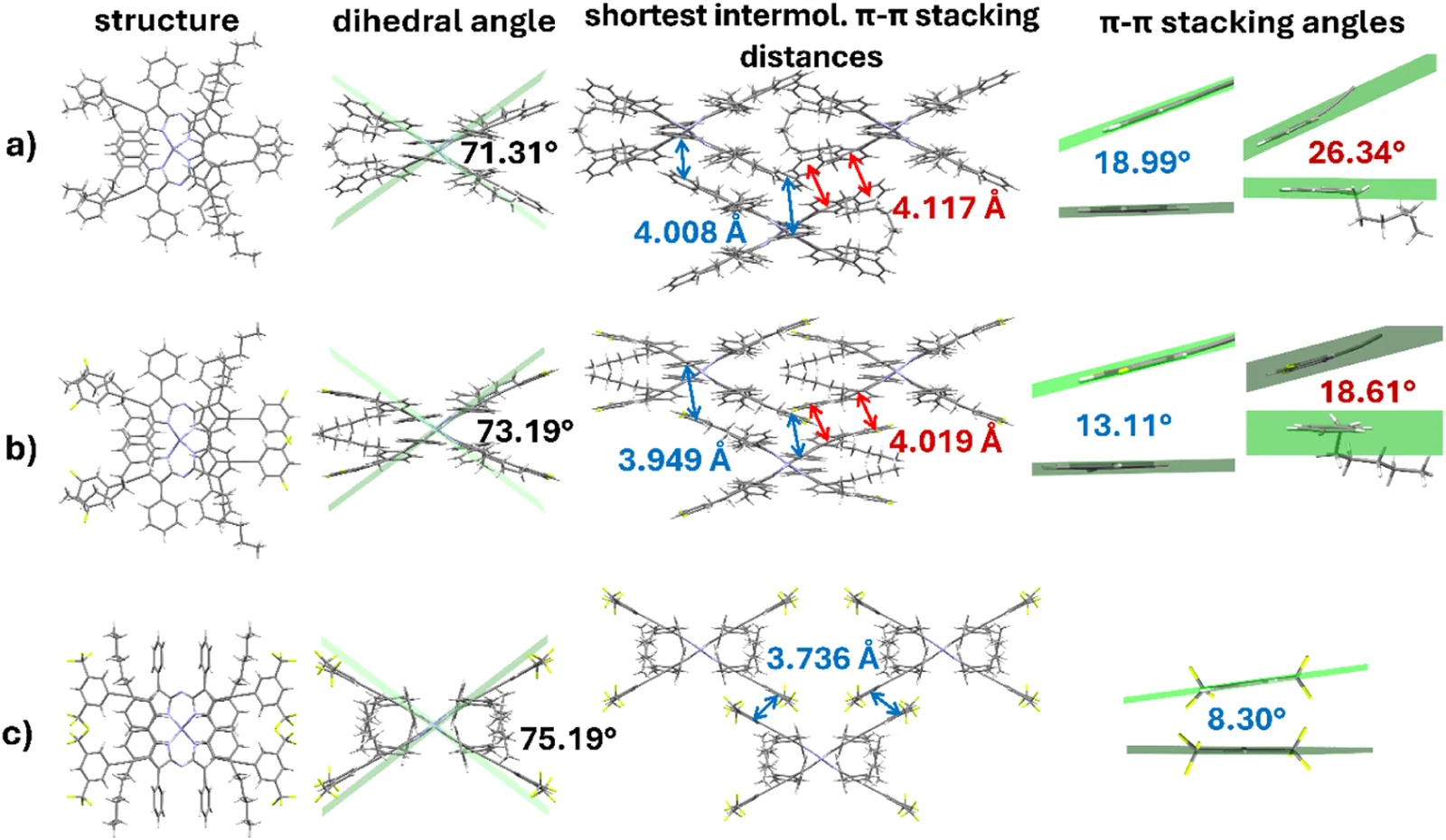
Capped stick structures, dihedral angles between the two ligands, shortest intermolecular π–π stacking distances (centroid-centroid), and π–π stacking angles between intermolecularly stacked phenyls (angle between the phenyl planes) for a) Zn­(L1)_2_, b) Zn­(L1-*m*-2F)_2_, and c) Zn­(L1-*m*-2CF_3_)_2_. For π-π stacking angles of a) and b), the left (blue) involves the pyrrolic-distal phenyls, whereas the right (red) involves the pyrrolic-proximal phenyls. THF solvent molecules were omitted to simplify the structure.

**Table 6:** Summary of Crystallographic Data of the Zn­(II) Complexes

Complex	Intramol. dihedral angle (deg)	Space group	Shortest intermol. distances (Å)^b^	Intermol. dihedral angle (deg)^c^	Groups involved in CF intermol. π–π stacking^d^
Zn(L1)_2_ ^a^	71.31	*C*2/*c*	4.008	18.99	Pyrrolic-distal^e^
			4.117	26.34	Pyrrolic-proximal
Zn(L1-*m*-2F)_2_	73.19	*C*2/*c*	3.949	13.11	Pyrrolic-distal
			4.019	18.61	Pyrrolic-proximal,
Zn(L1-*m*-2CF_3_)_2_	75.19	*Fddd*	3.736	8.30	Pyrrolic-pyrrolic

a Cif file obtained from the literature.^19^

b Intermolecular π–π stacking distance.

c Intermolecular π–π stacking dihedral angle of the shortest intermolecular π–π stacking distance.

d Phenyl groups involved in the shortest cofacial (CF) intermolecular π–π stacking.

e Proximal refers to the proximal phenyl (with the hexyl solubilizing group), pyrrolic refers to the phenylethynylpyrrolic group, and distal refers to the distal phenyl group (without a solubilizing group).

Figure 7 also shows the complexes’ crystal packing. The crystal packing structures of Zn­(L1)_2_ and Zn­(L1-*m*-2F)_2_ are similar with a *C*2/*c* space group, whereas Zn­(L1-*m*-2CF_3_)_2_ exhibited a more highly ordered packing structure with an *Fddd* space group. In Zn­(L1)_2_ and Zn­(L1-*m*-2F)_2_, the shortest intermolecular π–π stacking interactions occur between the pyrrolic phenyl and either the proximal phenyl (with the hexyl solubilizing group) or distal phenyls (without a solubilizing group) of a neighboring molecule. Both interactions occur in both Zn­(L1)_2_ and Zn­(L1-*m*-2F)_2_ and are similar in scale; thus, both are included for comparison. The pyrrolic-distal interaction is 4.008 Å for Zn­(L1)_2_ and 3.949 Å for Zn­(L1-*m*-2F)_2_, and the pyrrolic-proximal interaction is 4.117 Å for Zn­(L1)_2_ and 4.019 Å for Zn­(L1-*m*-2F)_2_. In both molecules, the pyrrolic-distal interaction is a slightly shorter distance than the pyrrolic-proximal interaction, which may be influenced by the steric hindrance of the hexyl group on the proximal phenyl. On the other hand, the shortest intermolecular π–π interactions in Zn­(L1-*m*-2CF_3_)_2_ show CF π–π stacking between the 3,5-bis­(trifluoromethyl)­phenylethynyl moieties (pyrrolic groups) of two neighboring molecules, with an intermolecular distance of 3.736 Å. In addition, the dihedral angles of the stacking interaction decreased with an increasing degree of fluorination: in Zn­(L1)_2_, the stacking angles are 18.99° (pyrrolic-distal) and 26.34° (pyrrolic-proximal); in Zn­(L1-*m*-2F)_2_, the stacking angles are 13.11° (pyrrolic-distal) and 18.61° (pyrrolic-proximal); and in Zn­(L1-*m*-2CF_3_)_2_, the stacking angle is 8.30° (pyrrolic-pyrrolic). Overall, −CF_3_ induced a more ordered crystal packing structure and promoted a more CF π–π stacking with smaller intermolecular distance than H or F. The π–π stacking interaction is a clear parallel-displaced type where the −CF_3_ groups of one group are oriented opposite to the other to minimize dipole moments. This could be due to the greater electron-withdrawing character and/or larger steric profile of trifluoromethyl groups.

### Internal Reorganization Energy and Overlap Integrals

We calculated two important parameters that are related to intrinsic charge carrier mobility at the microscopic level: internal reorganization energy (λ) and overlap integrals (V).^48^ The values of λ^+^ (for hole transfer) and λ^–^ (for electron transfer) were calculated using Nelsen’s four-point method and are summarized in Table 7. The λ^+^ values increased with fluorination from 0.057 eV for Zn­(L1)_2_ to 0.115 and 0.099 eV for Zn­(L1-*m*-2F)_2_ and Zn­(L1-*m*-2CF_3_)_2_, respectively. These values are still low and are comparable to pentacene (0.093–0.098 eV).^32,49^ The λ^–^ values are generally slightly larger than the λ^+^ values, which is common and could be due to greater structural changes when a charge is added to mostly antibonding LUMOs (λ^–^) than when a charge is removed from mostly bonding HOMOs (λ^+^).^33,50^ Interestingly, the complexes’ λ^–^ decreased with fluorination, going from 0.151 eV for Zn­(L1)_2_ to 0.119 and 0.114 eV for Zn­(L1-*m*-2F)_2_ and Zn­(L1-*m*-2CF_3_)_2_, respectively.

**Table 7:** Calculated Internal Reorganization Energies and Largest Overlap Integrals

Complex	λ^+^ (eV)	λ^–^ (eV)	Groups identified in CF intermol. π–π stacking	V_h+_ (meV)	V_e–_ (meV)
Zn(L1)_2_	0.057	0.151	Two pyrrolic-distal^a^	12.03	3.53
			Two pyrrolic-proximal	2.51	4.52
Zn(L1-*m*-2F)_2_	0.115	0.119	Two pyrrolic-distal	25.5	10.6
			Two pyrrolic-proximal	3.66	9.47
			Two distal-proximal	1.88	1.15
Zn(L1-*m*-2CF_3_)_2_	0.099	0.114	One pyrrolic-pyrrolic	23.2	7.49

a Proximal refers to the proximal phenyl (with the hexyl solubilizing group), pyrrolic refers to the phenylethynylpyrrolic group, and distal refers to the distal phenyl group (without a solubilizing group).

The overlap integrals were estimated via single-point energy calculations of the molecular pairs using the Amsterdam density functional (ADF) code. Tables S3–S5 show all pairs identified from the complexes’ crystal packing, along with the direction from the reference “red” molecule, visually identified groups involved in intermolecular interactions, and calculated hole overlap integrals (V_h+_) and electron overlap integrals (V_e–_). The most significant overlaps are summarized in Table 7. Zn­(L1)_2_ showed six distinct pairs with overlap of at least 0.1 meV (with each pair having another symmetrical pair, for a total of 12 neighboring molecules to the selected Zn­(L1)_2_ molecule). The pair with the largest overlap integral has a V_h+_ and V_e–_ of 12.03 and 3.53 meV, respectively. This pair corresponds to the same molecules that show a nearly CF pyrrolic-distal interaction as in Figure 7a. We note that within a pair, there are two distinct pyrrolic-distal interactions, yielding the large overlap integral. The pair with the next largest V corresponds to the same molecules that show a nearly CF pyrrolic-proximal interaction as in Figure 7a. This interaction also occurs twice between the two molecules. For this pair, V_e–_ is larger than V_h+_, at 4.52 meV for V_e–_ versus 2.51 meV for V_h+_, providing a rare example where a specific arrangement leads to larger overlap for electron transport.^35^ In the other pairs, the closest interacting aryl groups are very displaced or more edge-to-edge in orientation, resulting in much weaker overlap. Figure S23 plots the pairs with significant overlap (>1 meV), showing that most of the overlap for Zn­(L1)_2_ occurs in a two-dimensional plane. The pairs with lower overlap listed in Table S3 (<1 meV) provide some directional transport out of the plane.

Zn­(L1-*m*-2F)_2_ showed seven distinct pairs with an overlap of at least 0.1 meV (14 total neighboring molecules). Since the crystal structures of Zn­(L1-*m*-2F)_2_ and Zn­(L1)_2_ have the same *C*2/*c* space group, it is possible to compare V for analogous pairs. For both pairs involving CF intermolecular interactions (pyrrolic-distal and pyrrolic-proximal, as in Figure 7b), the overlap integrals significantly increased (roughly doubled) with fluorination, reaching V_h+_ of 25.5 meV and V_e–_ of 10.6 meV for the CF pyrrolic-distal pair and V_h+_ of 3.66 meV and V_e–_ of 9.47 meV for the CF pyrrolic-proximal pair. As a result, this complex has a high V_e–_ in two dimensions. These values are in the same range as those calculated for oligoacenes that have high charge carrier mobilities.^35^ Interestingly, the pair involving distal and proximal phenyls have significantly higher V_h+_ (3-fold increase from 0.56 to 1.88 meV) and V_e–_ (5-fold increase from 0.22 to 1.15 meV) in Zn­(L1-*m*-2F)_2_ than Zn­(L1)_2_. Figure S24 plots the pairs with significant overlap (>1 meV), which includes the distal/proximal pair. This additional pair provides a significant path for electron transfer out of the plane, a prerequisite for isotropic charge transport.

Zn­(L1-*m*-2CF_3_)_2_ showed three distinct pairs, which each have three other symmetrical pairs, giving 12 total neighboring molecules. The pairs with the highest overlap all involve one pyrrolic group of one molecule interacting with one pyrrolic group of another molecule, as described in Figure 7c. This CF interaction gives a V_h+_ of 23.2 meV and V_e–_ = 7.49 meV. Surprisingly, these overlaps were slightly lower than for the distal-pyrrolic pair in Zn­(L1-*m*-2F)_2_, even though the identified π–π stacking interaction in Zn­(L1-*m*-2CF_3_)_2_ should be stronger based on its much shorter distance (3.7 Å versus ∼4.0 Å). The higher overlap integral observed in the Zn­(L1-*m*-2F)_2_ pair illustrates that optimizing one intermolecular π–π stacking interaction is not sufficient to optimize electron transfer and that other factors such as relative positions of the two molecules matter. Figure S25 plots the pairs of Zn­(L1-*m*-2CF_3_)_2_ with strong overlap (>1 meV) consisting of only one distinct pair (and three symmetrically identical pairs), showing that most of the transport is in a two-dimensional plane. The pairs with weaker overlap would allow directional electron transfer out of the plane but are small in magnitude compared to the dominant pairs.

## Conclusions

Zn­(II) complexes of 2,8-phenylethynyl 1,3,7,9-tetraphenyl azadipyrromethenes with −F or −CF_3_ groups at the *meta* positions of the phenylethynyls were successfully synthesized. Fluorination slightly affected thermal properties by increasing melting points and decreasing decomposition temperatures, had minimal effects on optical properties and slightly increased both IP and EA. On the other hand, fluorination strongly affected crystal packing: fluorination encouraged more cofacial π–π stacking, and −CF_3_ groups induced a more symmetrical crystal packing structure than with −F or −H groups. The −CF_3_ groups also encouraged strong cofacial π–π stacking between the pyrrolic groups of adjacent molecules. Parameters relevant to microscopic intrinsic charge transport were calculated. Reorganization energies were generally low due to these compounds’ highly rigid conjugated structure, and fluorination decreased λ^–^. Overlap integral calculations revealed that fluorination greatly improves both V_h+_ and V_e–_. Interestingly, both fluorinated complexes had similar and relatively high V_e–_ in two dimensions, with Zn­(L1-*m*-2F)_2_ giving the larger overlap integrals than Zn­(L1-*m*-2CF_3_)_2_. While all showed large overlap integrals within a 2-D plane, Zn­(L1-*m*-2F)_2_ showed significant overlap in a third dimension, required for isotropic electron transfer. It is important to point out that these calculations are based on crystal structure data and do not necessarily represent what happens in a film. We are thus currently investigating the effect of fluorination on film morphology and charge carrier mobility; the results will be published separately.

## Experimental Section

### Methods

^1^H, ^13^C, and ^19^F spectra were recorded on a 500 MHz Bruker Ascend Avance III HD spectrometer. Chemical shifts (^1^H) are reported in parts per million relative to Si­(CH_3_)_4_. Elemental analyses (C, H, and N) were performed under optimum combustion conditions by Robertson Microlit Laboratories (Ledgewood, NJ). Matrix-assisted laser-desorption/ionization time-of-flight (MALDI-TOF) mass spectra were collected using a Bruker Autoflex instrument. A 0.25 mg/mL solution of the Zn­(II) complexes in THF and a 1 mg/mL solution of α-CNPV-CH3 (matrix)^51^ were mixed in a 1:1 volume ratio and spotted onto the sample well. The MALDI-TOF mass spectra were obtained using a negative reflectron mode. The laser strength was kept between 20 and 25% to maximize signal and minimize fragmentation. The isotopic patterns were calculated using the Scientific Instrument Services (SIS) Isotope Distribution Calculator available for free online (https://www.sisweb.com/mstools/isotope.htm).

### Crystallography

Single crystals were obtained by slowly evaporating THF solvent from pure samples of the Zn­(II) complexes. THF was added to sufficiently dissolve the complexes and was allowed to evaporate at room temperature over a period of 3–4 days in a well-ventilated area. Single crystals of Zn­(L1-*m*-2F)_2_ or Zn­(L1-*m*-2CF_3_)_2_ were coated with a trace of Fomblin oil and quickly transferred to the goniometer head of a Bruker Quest diffractometer with a fixed chi angle, a sealed tube fine focus X-ray tube, a single crystal curved graphite incident beam monochromator, a Photon II area detector, and an Oxford Cryosystems low temperature device. Examination and data collection were performed with Mo Kα radiation (λ = 0.71073 Å) at 150(2) K.

Data were collected, reflections were indexed and processed, and the files scaled and corrected for absorption using APEX5 and SADABS.^52,53^ The space groups were assigned using XPREP within the SHELXTL suite of programs^54,55^ and solved by direct methods using ShelXS^55^ or dual methods using ShelXT^56^ and refined by full matrix least-squares against F^2^ with all reflections using Shelxl-2019 ^57,58^ using the graphical interface Shelxle.^59^ If not specified otherwise, H atoms attached to carbon, boron, and nitrogen atoms, as well as hydroxyl hydrogens, were positioned geometrically and constrained to ride on their parent atoms. C–H bond distances were constrained to 0.95 Å for aromatic and alkene C–H and CH_2_ and alkyne C–H moieties and to 1.00, 0.99, and 0.98 Å for aliphatic C–H, CH_2_, and CH_3_ moieties, respectively. Methyl CH_3_ H atoms were allowed to rotate but not to tip to best fit the experimental electron density. U_iso_(H) values were set to a multiple of U_eq_(C) with 1.5 for CH_3_ and 1.2 for C–H and CH_2_ units, respectively. The crystallographic data tables (pages S23–S50 in the Supporting Information) were obtained using FinalCIF. The capped stick structures of the molecules (Figure 7) were obtained using the Mercury crystallographic software. The reported intermolecular distances and dihedral angles were obtained by analyzing the crystal packing using Mercury. For distances, the phenyl rings were assigned a centroid and the distance was measured between the centroids. For angles, the phenyl rings were assigned a plane and the dihedral angle was measured between the two planes.

### Thermal Measurements

Thermogravimetric analysis (TGA) was performed on a TA Instruments Q500 thermogravimetric analyzer using a platinum pan. Samples were analyzed from 25 to 800 °C at a ramp rate of 10 °C/min. Differential scanning calorimetry (DSC) was performed on a TA Instruments 250 Discovery differential scanning calorimeter. Approximately 2 mg samples were prepared for DSC and analyzed from 0 to 250 °C for Zn­(L1)_2_ or from 0 to 400 °C for the fluorinated complexes, all at a ramp rate of 10 °C/min. Since the DSC plots were featureless from 250 to 400 °C, the DSC figure only shows data between 0 and 250 °C.

### Optical Properties

UV–visible absorption spectra were collected on an Agilent Cary 5000 Vis-UV-NIR spectrophotometer in chloroform. Spin coating of films was performed on precut microscopic glass substrates using a Laurell Spin Coater WS-650. The glass substrates were cleaned using detergent, DI water, acetone, and IPA by sonicating for 15 min in each solution and dried with nitrogen before use. Thin films were prepared from 10 mg/mL solutions in HPLC-grade chloroform, filtered through a 0.45 μm PTFE filter, and spin-coated at 800 rpm for 40 s, followed by 2000 rpm for 2 s. The films were then annealed at 150 °C for 10 min in ambient air.

### Electrochemical Measurements

Electrochemical data were collected at room temperature using an Autolab PGSTAT 302N Exo Chemie potentiostat. Cyclic voltammetry (CV) was performed using 0.1 M Bu_4_NPF_6_ electrolyte solution in dry dichloromethane and a ferrocene/ferrocenium internal standard. The solution was purged with N_2_ for at least 15 min prior to testing. A glassy carbon electrode served as the working electrode, and a Pt electrode served as the quasi-reference electrode. Both electrodes were freshly polished prior to each measurement. A Pt wire served as the counter electrode. Approximately 5 mg of analyte was fully dissolved in 15 mL of electrolyte solution. Measurements were performed at a scan rate of 0.5 V/s. The highest-occupied molecular orbital (HOMO) and lowest-unoccupied molecular orbital (LUMO) energy levels were estimated from the onset of oxidation and reduction potentials, respectively, using the equations:

$$HOMO=-\left(E_{onset,ox\;vs\;Fc/Fc^{+}}+4.8eV\right)$$

$$LUMO=-\left(E_{onset,red\;vs\;Fc/Fc^{+}}+4.8eV\right)$$

### Computational Methods

The molecules’ structures were constructed using Avogadro 1.2.0. The hexyl solubilizing groups were replaced with methyl groups to simplify the calculations. Lowest energy conformers for each Zn­(II) complex were generated using XTB Global Geometry Optimization and Ensemble Generator (XTB-GOAT, version 6.4.0). Geometry optimizations and single-point energy calculations of the lowest-energy conformers were performed in ORCA 6.1.0. All DFT calculations were carried out using the TPSS functional, the def2-TZVP basis set, tight SCF convergence, D3 dispersion corrections with the Becke–Johnson damping scheme (D3BJ), and resolution of the identity with the def2/J auxiliary basis set. Solvation effects were included using the conductor-like polarizable continuum model (CPCM) with a dielectric constant of 8.93 (DCM), using defgrid3 for improved numerical accuracy. For a quantitative assessment of the energy levels, vertical IP and EA were calculated using the ΔSCF, in which the energy differences between the neutral and corresponding charged states were evaluated at the ground-state geometry. The electron reorganization energy (λ^–^) and hole reorganization energy (λ^+^) were calculated using Nelsen’s four-point method:

$$\lambda^{+}=E_{0}\left(Q_{+}\right)-E_{0}\left(Q_{0}\right)+E_{+}\left(Q_{0}\right)-E_{+}\left(Q_{+}\right)$$

$$\lambda^{-}=E_{0}\left(Q_{-}\right)-E_{0}\left(Q_{0}\right)+E_{-}\left(Q_{0}\right)-E_{-}\left(Q_{-}\right)$$

where E is energy, Q is geometry, and the subscripts 0, +, and – denote neutral, cationic, and anionic states, respectively.^60–62^

The overlap integrals were estimated via single-point energy calculations of the molecular pairs. The pairs were obtained from the crystal structure by using Mercury 3.10.2. The charge-transfer integral *V* was calculated using the Amsterdam density functional (ADF) code in the SCM Amsterdam Modeling Suite (2026). To simplify the calculations, the hexyl side chains were manually replaced with methyl groups using the ADF software. We followed the instructions for the tutorial from the SCM website.^63^ Each complex was manually defined as one fragment for a total of two fragments. Single-point calculations were performed using the GGA:PW91 functional, TZP basis set, and no frozen core to guarantee an all-electron basis set. Other selections included charge transfer integral properties, no symmetry, multilevel used fragments, and checked charge and spin. Note that the TZP basis set has been shown to give reliable results.^64^ We calculated the overlap integrals for one pair using different methods (MetaGGA:TPSS/TZP, MetaGGA:TPSS/TZ2P, MetaGGA:TPSS-D3/TZP, and MetaGGA:SCAN/TZP) and found the same results as the established GGA:PW91/TZP method.

#### Synthesis

All chemicals were purchased from Sigma-Aldrich, Fisher Scientific, TCI America, or Acros Organics and used as received. The syntheses of 4′-hexylchalcone and phexADP were done according to the literature.^19^

**4-hexylchalcone.** The product is a crystalline white solid (10.44 g, 35.73 mol, 83.79% yield). ^1^H NMR (500 MHz, CDCl_3_) δ 7.96 (d, *J* = 7.7 Hz, 2H), 7.81 (d, *J* = 15.7 Hz, 1H), 7.68–7.63 (m, 2H), 7.55 (d, *J* = 15.7 Hz, 1H), 7.42 (d, *J* = 5.3 Hz, 3H), 7.31 (d, *J* = 7.9 Hz, 2H), 2.69 (t, *J* = 7.8 Hz, 2H), 1.65 (p, *J* = 7.4 Hz, 2H), 1.36 (d, *J* = 6.8 Hz, 1H), 1.31 (q, *J* = 6.7 Hz, 5H), 0.89 (s, 2H), 0.89 (d, *J* = 13.2 Hz, 1H).

**phexADP.** The product is a shiny brown solid (5.21 g, 8.44 mmol, 48.0% yield). ^1^H NMR (500 MHz, CDCl_3_) δ 8.07 (d, *J* = 7.6 Hz, 2H), 7.88 (d, *J* = 7.7 Hz, 2H), 7.43 (t, *J* = 7.5 Hz, 2H), 7.35 (d, *J* = 7.7 Hz, 3H), 7.19 (s, 1H), 2.70 (t, *J* = 7.8 Hz, 2H), 1.68 (p, *J* = 7.6 Hz, 2H), 1.42–1.30 (m, 6H), 0.91 (d, *J* = 6.6 Hz, 3H).

**Tributyl­(phenylethynyl)­stannane.** Ethynylbenzene (8.00 g, 78.4 mmol) and 20 mL dry THF were added to a dry 100 mL Schlenk flask under nitrogen. The solution was cooled to −78 °C in an acetone-dry ice bath. *n*-Butyllithium solution (28 mL, 70 mmol, 2.5 M in Et_2_O) was added dropwise to the solution and was stirred at −78 °C for 1 h. Tributyltin chloride (25 g, 76.8 mmol) was added to the solution. **Caution!** Organotin compounds are highly toxic via ingestion and dermal absorption. All manipulations must be performed inside a ventilated fume hood using personal protective equipment, including a lab coat, safety goggles, and chemical-resistant gloves. The flask was removed from the acetone-dry ice bath, and the solution was stirred at 25 °C for 30 min. Solvents were removed from the flask by rotary evaporation, yielding a cloudy yellow liquid. The crude mixture was passed through a short plug of neutral alumina with hexanes. Hexanes were removed by rotary evaporation to yield the product as a light-yellow oil (28.9 g, 73.9 mmol, 94.2% yield). ^1^H NMR (500 MHz, CDCl_3_) δ 7.40–7.34 (m, 2H), 7.21–7.15 (m, 3H), 1.59–1.48 (m, 6H), 1.34–1.25 (m, 6H), 1.03–0.92 (m, 6H), 0.85 (t, J = 7.3 Hz, 9H).

**Tributyl­((3,5-difluorophenyl)­ethynyl)­stannane.** 1-ethynyl-3,5-difluorobenzene (2.05 g, 14.8 mmol) and 10 mL dry THF were added to a dry 50 mL Schlenk flask under nitrogen. The solution was cooled to −78 °C in an acetone-dry ice bath. *n*-Butyllithium solution (5.8 mL, 14.5 mmol, 2.5 M in Et_2_O) was added dropwise to the solution and was stirred at −78 °C for 1 h. Tributyltin chloride (5.2 g, 16.0 mmol) was added to the solution. The flask was removed from the acetone-dry ice bath, and the solution was stirred at 25 °C for 30 min. Solvents were removed from the flask by rotary evaporation, obtaining a cloudy yellow liquid. The crude mixture was passed through a short plug of neutral alumina with hexanes. Hexanes were removed by rotary evaporation to yield the product as a light-yellow oil (6.12 g, 14.3 mmol, 96.6% yield). ^1^H NMR (500 MHz, CDCl_3_) δ 6.97–6.91 (m, 2H), 6.73 (tt, J = 9.0, 2.4 Hz, 1H), 1.66–1.57 (m, 6H), 1.38 (h, J = 7.4 Hz, 6H), 1.12–1.03 (m, 6H), 0.94 (t, J = 7.3 Hz, 9H).

**((3,5-bis­(trifluoromethyl)­phenyl)­ethynyl)­tributylstannane.** 1-ethynyl-3,5-bis­(trifluoromethyl)­benzene (2.92 g, 12.9 mmol) and 10 mL dry THF were added to a dry 50 mL Schlenk flask under nitrogen. The solution was cooled to −78 °C in an acetone-dry ice bath. *n*-Butyllithium solution (5.1 mL, 13 mmol, 2.5 M in Et_2_O) was added dropwise to the solution and was stirred at −78 °C for 1 h. Tributyltin chloride (4.8 g, 15 mmol) was added to the solution. The flask was removed from the acetone-dry ice bath, and the solution was stirred at 25 °C for 30 min. Solvents were removed from the flask by rotary evaporation, obtaining a cloudy brown liquid. The crude mixture was passed through a short plug of neutral alumina with hexanes. Hexanes were removed by rotary evaporation to yield the product as a light-yellow oil (6.52 g, 12.4 mmol, 96.1% yield). ^1^H NMR (500 MHz, CDCl_3_) δ 7.85 (s, 2H), 7.75 (s, 1H), 1.68–1.55 (m, 6H), 1.39 (h, J = 7.4 Hz, 6H), 1.15–1.05 (m, 6H), 0.94 (t, J = 7.4 Hz, 9H).

**phexADPI**_**2**_. phexADP (5.05 g, 8.18 mmol), N-iodosuccinimide (4.61 g, 20.5 mmol), glacial acetic acid (15 drops), and 200 mL N_2_-degassed chloroform were added to a 500 mL round-bottom flask. The flask was covered in aluminum foil to keep the mixture in the dark, and the mixture was stirred at 25 °C for 16 h. Chloroform was removed by rotary evaporation. The resulting solids were resuspended in methanol, then vacuum filtrated to isolate the solids, washing with methanol. The product was dried under vacuum to yield a dark purple solid (4.33 g, 4.98 mmol, 60.9% yield). ^1^H NMR (500 MHz, CDCl_3_) δ 7.91 (d, *J* = 7.8 Hz, 1H), 7.70 (d, *J* = 7.0 Hz, 1H), 7.40–7.30 (m, 3H), 2.70 (t, *J* = 7.8 Hz, 1H), 1.68 (p, *J* = 7.5 Hz, 1H), 1.40 (d, *J* = 7.6 Hz, 1H), 1.34 (d, *J* = 6.3 Hz, 3H), 0.94–0.85 (m, 2H).

**L1.** phexADPI_2_ (1.35 g, 1.55 mmol) was added to a 250 mL 3-neck Schlenk flask equipped with a reflux condenser. The flask was purged three times with nitrogen, then Pd­(PPh_3_)_4_ (224 mg, 194 μmol) and 120 mL xylenes were added to the flask. The solution was stirred at 135 °C for 1 h. Tributyl­(phenylethynyl)­stannane (2.01 g, 5.14 mmol) was added to the flask, and the solution was stirred at 135 °C for 16 h. Xylenes were removed by rotary evaporation, leaving a dark blue solid, which was resuspended in methanol. Solids were isolated by vacuum filtration and washed with methanol, yielding the product as a dark blue solid (1.023 g, 1.25 mmol, 80.7% yield). ^1^H NMR (500 MHz, CDCl_3_) δ 8.28 (d, J = 7.9 Hz, 4H), 8.20 (d, J = 7.7 Hz, 4H), 7.49 (d, J = 7.2 Hz, 4H), 7.47–7.39 (m, 6H), 7.39–7.34 (m, 10H), 2.73 (t, J = 7.9 Hz, 4H), 1.71 (p, J = 7.8 Hz, 4H), 1.37–1.35 (m, 12H), 0.93–0.92 (m, 6H).

**L1-** *m* **-2F.** phexADPI_2_ (1.17 g, 1.35 mmol) was added to a 250 mL 3-neck Schlenk flask equipped with a reflux condenser. The flask was purged three times with nitrogen, then Pd­(PPh_3_)_4_ (161 mg, 139 μmol) and 120 mL xylenes were added to the flask. The solution was stirred at 135 °C for 1 h. Tributyl­((3,5-difluorophenyl)­ethynyl)­stannane (1.39 g, 3.25 mmol) was added to the flask, and the solution was stirred at 135 °C for 16 h. Xylenes were removed by rotary evaporation, leaving a dark blue solid, which was resuspended in methanol. Solids were isolated by vacuum filtration and washed with methanol, yielding the product as a dark blue-purple solid (0.564 g, 0.634 mmol, 47.0% yield). ^1^H NMR (500 MHz, CDCl_3_) δ 8.18 (d, J = 7.7 Hz, 4H), 8.10 (d, J = 7.3 Hz, 4H), 7.48–7.34 (m, 10H), 6.95 (d, J = 6.6 Hz, 4H), 6.79 (t, J = 8.9 Hz, 2H), 2.74 (t, J = 8.0 Hz, 4H), 1.71 (p, J = 7.8 Hz, 4H), 1.44–1.31 (m, 12H), 0.91 (t, J = 6.5 Hz, 6H). ^19^F NMR (471 MHz, CDCl_3_) δ −109.48.

**L1-** *m* **-2CF**_**3**_. phexADPI_2_ (1.46 g, 1.68 mmol) was added to a 250 mL 3-neck Schlenk flask equipped with a reflux condenser. The flask was purged three times with nitrogen, then Pd­(PPh_3_)_4_ (228 mg, 197 μmol) and 150 mL xylenes were added to the flask. The solution was stirred at 135 °C for 1 h. ((3,5-bis­(trifluoromethyl)­phenyl)­ethynyl)­tributylstannane (5.23 g, 9.92 mmol) was added to the flask, and the solution was stirred at 135 °C for 16 h. Xylenes were removed by rotary evaporation, leaving a dark blue solid, which was resuspended in methanol. Solids were isolated by vacuum filtration and washed with methanol, then cold chloroform, yielding the product as a dark blue-purple solid (0.986 g, 0.904 mmol, 53.8% yield). No NMR spectrum could be obtained due to the poor solubility in organic solvents.

### General Procedure for Zn­(II) Chelation

The ligand was added with sodium hydride (5 equiv) to a 100 mL 3-neck Schlenk flask under nitrogen. Anhydrous THF (approximately 100 mL per 1 g ligand) was added to the flask, and the mixture was stirred at 65 °C for 3 h. The mixture was cooled to 25 °C, then ZnCl_2_ solution (1.0 M in diethyl ether, 5 equiv) was added to the flask, and the mixture was stirred at 25 °C for 16 h. Solvents were removed by rotary evaporation, then resuspended in methanol and isolated by vacuum filtration. The product was recrystallized by liquid diffusion of THF and methanol, then dried under vacuum. If residual metal impurities were detected during characterization, the solid was stirred in a solution of 0.2 M EDTA (aqueous, pH 9) for 30 min, then filtered and washed with water and methanol.

**Zn­(L1)**_**2**_. L1 (270 mg, 0.330 mmol), sodium hydride (40 mg, 1.7 mmol), 30 mL anhydrous THF, and ZnCl_2_ solution (1.70 mL, 1.70 mmol). The product is a dark blue solid (151 mg, 88.8 μmol, 53.8%). ^1^H NMR (500 MHz, CDCl_3_) δ 8.08–8.03 (m, 8H), 7.62 (d, J = 7.7 Hz, 8H), 7.45–7.40 (m, 12H), 7.37 (d, J = 7.4 Hz, 8H), 7.33–7.27 (m, 12H), 7.00 (d, J = 7.7 Hz, 8H), 2.34 (t, J = 8.2 Hz, 8H), 1.36 (p, J = 7.4 Hz, 8H), 1.28–1.07 (m, 24H), 0.85 (t, J = 7.3 Hz, 12H). ^13^C NMR (126 MHz, CDCl_3_) δ 162.07, 146.57, 145.61, 145.00, 133.43, 131.18, 131.08, 129.73, 128.43, 128.01, 127.93, 127.69, 124.20, 111.99, 96.71, 85.95, 35.93, 31.77, 30.93, 29.50, 22.84, 14.31. Elemental Analysis. Calculated: C, 84.80; H, 6.41; N, 4.94. Measured: C, 84.68; H, 6.46; N, 4.68. MALDI-TOF: calculated for C_120_H_108_N_6_Zn, exact mass 1696.79 *m*/*z*; measured (centroid mass), 1700.47 *m*/*z*.

**Zn­(**L1-*m*-2F)_**2**_. L1-*m*-2F (489 mg, 0.549 mmol), sodium hydride (78 mg, 2.6 mmol), 50 mL anhydrous THF, and ZnCl_2_ solution (3.05 mL, 3.05 mmol). The product is a dark blue solid (230 mg, 125 μmol, 45.5%). ^1^H NMR (500 MHz, CDCl_3_) δ 7.97 (d, J = 6.8 Hz, 8H), 7.55 (d, J = 7.7 Hz, 8H), 7.47–7.42 (m, 12H), 7.02 (d, J = 7.6 Hz, 8H), 6.85 (d, J = 6.5 Hz, 8H), 6.74 (t, J = 9.1 Hz, 4H), 2.35 (t, J = 8.2 Hz, 8H), 1.35 (p, J = 7.5 Hz, 8H), 1.28–1.07 (m, 24H), 0.85 (t, J = 7.3 Hz, 12H). ^13^C NMR (126 MHz, CDCl_3_) δ 163.91, 162.41, 161.93, 161.82, 146.51, 146.20, 146.07, 133.00, 130.99, 129.46, 128.51, 128.27, 127.85, 127.82, 126.63, 114.03, 113.97, 113.87, 113.82, 111.12, 104.08, 94.49, 87.93, 68.14, 35.94, 31.79, 30.99, 29.48, 25.77, 22.81, 14.27. ^19^F NMR (471 MHz, CDCl3) δ −109.75, −109.77 (t, J = 40.1 Hz). Elemental Analysis. Calculated: C, 78.18; H, 5.47; N, 4.56. Measured: C, 77.95; H, 5.64; N, 4.43. MALDI-TOF: calculated for C_120_H_100_F_8_N_6_Zn, exact mass 1840.72 *m*/*z*; measured (centroid mass), 1844.558 *m*/*z*.

**Zn­(L1-** *m* **-2CF**_**3**_)_**2**_. L1-*m*-2CF_3_ (202 mg, 0.185 mmol), sodium hydride (29 mg, 1.2 mmol), 20 mL anhydrous THF, and ZnCl_2_ solution (1.00 mL, 1.00 mmol). The product is a dark blue solid (103 mg, 46.0 μmol, 49.7%). ^1^H NMR (500 MHz, CDCl_3_) δ 7.97 (d, J = 7.1 Hz, 8H), 7.74 (s, 4H), 7.72 (s, 8H), 7.57 (d, J = 7.7 Hz, 8H), 7.54–7.44 (m, 12H), 7.05 (d, J = 7.6 Hz, 8H), 2.38 (t, J = 8.1 Hz, 8H), 1.37 (p, J = 7.6 Hz, 8H), 1.27–1.07 (m, 24H), 0.84 (t, J = 7.2 Hz, 12H). ^13^C NMR (126 MHz, CDCl_3_) δ 162.68, 146.75, 146.51, 146.39, 132.83, 132.23, 131.96, 130.99, 130.76, 129.38, 128.82, 128.29, 127.96, 126.22, 124.17, 122.00, 110.78, 93.75, 89.57, 68.14, 35.98, 31.76, 31.08, 29.44, 25.77, 22.74, 14.18. ^19^F NMR (471 MHz, CDCl_3_) δ −63.17. Elemental Analysis. Calculated: C, 68.52; H, 4.49; N, 3.75. Measured: C, 68.23; H, 4.75; N, 3.64. MALDI-TOF: calculated for C_128_H_100_F_24_N_6_Zn, exact mass 2240.69 *m*/*z*; measured (centroid mass), 2244.661 *m*/*z*.

## Supplementary Material



## Notes

^1^H, ^13^C, and ^19^F NMR spectra of all synthesized compounds, MALDI-TOF mass spectra of the Zn­(II) complexes, crystallographic data tables of the new fluorinated Zn­(II) complexes, calculated HOMO/LUMO contour plots of the Zn­(II) complexes, and atomic coordinates used for DFT calculations of the Zn­(II) complexes (PDF)

## References

[1] Hains A. W., Liang Z., Woodhouse M. A., Gregg B. A. (2010). Molecular Semiconductors
in Organic Photovoltaic Cells. Chem. Rev..

[2] Zhang G., Zhao J., Chow P. C. Y., Jiang K., Zhang J., Zhu Z., Zhang J., Huang F., Yan H. (2018). Nonfullerene Acceptor
Molecules for Bulk Heterojunction Organic Solar Cells. Chem. Rev..

[3] Sirringhaus H. (2014). 25th anniversary
article: organic field-effect transistors: the path beyond amorphous
silicon. Adv. Mater..

[4] Banerjee S., Singh P., Purkayastha P., Ghosh S. K. (2025). Evolution of Organic
Light Emitting Diode (OLED) Materials and their Impact on Display
Technology. Chem. - Asian J..

[5] Chen J., Zhang W., Wang L., Yu G. (2023). Recent Research
Progress
of Organic Small-Molecule Semiconductors with High Electron Mobilities. Adv. Mater..

[6] Kang H., Lee W., Oh J., Kim T., Lee C., Kim B. J. (2016). From Fullerene-Polymer
to All-Polymer Solar Cells: The Importance of Molecular Packing, Orientation,
and Morphology Control. Acc. Chem. Res..

[7] Li D., Zhang X., Liu D., Wang T. (2020). Aggregation of non-fullerene
acceptors in organic solar cells. J. Mater.
Chem. A.

[8] Sauvé G., Fernando R. (2015). Beyond Fullerenes: Designing Alternative Molecular
Electron Acceptors for Solution-Processable Bulk Heterojunction Organic
Photovoltaics. J. Phys. Chem. Lett..

[9] Nielsen C. B., Holliday S., Chen H.-Y., Cryer S. J., McCulloch I. (2015). Non-Fullerene
Electron Acceptors for Use in Organic Solar Cells. Acc. Chem. Res..

[10] Nishimoto H., Kadoya T., Miyake R., Oda T., Nishida J.-I., Kubo K., Tajima H., Kawase T., Yamada J.-I. (2022). An isotropic
three-dimensional organic semiconductor 2-(thiopyran-4-ylidene)-1,3-benzodithiole
(TP-BT): asymmetric molecular design to suppress access resistance. CrystEngComm.

[11] Radford C. L., Hendsbee A. D., Abdelsamie M., Randell N. M., Li Y., Toney M. F., Kelly T. L. (2018). Effect
of Molecular Shape on the
Properties of Non-Fullerene Acceptors: Contrasting Calamitic Versus
3D Design Principles. ACS Appl. Energy Mater..

[12] Kumar R., Aggarwal H., Srivastava A. (2020). Of Twists and Curves: Electronics,
Photophysics, and Upcoming Applications of Non-Planar Conjugated Organic
Molecules. Chemistry.

[13] Li S., Zhang Z., Shi M., Li C.-Z., Chen H. (2017). Molecular
electron acceptors for efficient fullerene-free organic solar cells. Phys. Chem. Chem. Phys..

[14] Armin A., Li W., Sandberg O. J., Xiao Z., Ding L., Nelson J., Neher D., Vandewal K., Shoaee S., Wang T. (2021). A History
and Perspective of Non-Fullerene Electron Acceptors for
Organic Solar Cells. Adv. Energy Mater..

[15] Zhang X., Zhan C., Yao J. (2015). Non-Fullerene Organic
Solar Cells
with 6.1% Efficiency through Fine-Tuning Parameters of the Film-Forming
Process. Chem. Mater..

[16] Sun P., Li X., Wang Y., Shan H., Xu J., Liu C., Zhang C., Chen F., Xu Z., Chen Z. K., Huang W. (2018). Diketopyrrolopyrrole-based
acceptors with multi-arms for organic
solar cells. RSC Adv..

[17] Hildebrand M., Holst D., Bender T., Kronik L. (2022). Electronic Structure,
Bonding, and Stability of Boron Subphthalocyanine Halides and Pseudohalides. Adv. Theory Simul..

[18] Morse G. E., Bender T. P. (2012). Boron Subphthalocyanines as Organic Electronic Materials. ACS Appl. Mater. Interfaces.

[19] Wang C., Wei P., Ngai J. H. L., Rheingold A. L., Gray T. G., Li Y., Pentzer E., Li R., Zhu L., Sauvé G. (2019). A zinc­(II)
complex of di­(naphthylethynyl)­azadipyrromethene with low synthetic
complexity leads to OPV with high industrial accessibility. J. Mater. Chem. A.

[20] Sauve G. (2019). Designing
alternative non-fullerene molecular electron acceptors for solution-processable
organic photovoltaics. Chem. Rec..

[21] Teets T. S., Partyka D. V., Updegraff J. B., Gray T. G. (2008). Homoleptic, four-coordinate
azadipyrromethene complexes of d(10) zinc and mercury. Inorg. Chem..

[22] Bessette A., Ferreira J. G., Giguere M., Belanger F., Desilets D., Hanan G. S. (2012). Azadipyrromethene
dye derivatives in coordination chemistry:
the structure-property relationship in homoleptic metal­(II) complexes. Inorg. Chem..

[23] Mao Z., Senevirathna W., Liao J. Y., Gu J., Kesava S. V., Guo C., Gomez E. D., Sauvé G. (2014). Azadipyrromethene-Based Zn­(II) Complexes
as Nonplanar Conjugated Electron Acceptors for Organic Photovoltaics. Adv. Mater..

[24] DiScipio R., Sauvé G., Crespo-Hernández C. E. (2018). Photodynamics in
Metal-Chelating Tetraphenylazadipyrromethene Complexes: Implications
for Their Potential Use as Photovoltaic Materials. J. Phys. Chem. C.

[25] Wang C., Daddario C., Pejić S., Sauvé G. (2020). Synthesis
and Properties of Azadipyrromethene-Based Complexes with Nitrile Substitution. Eur. J. Org. Chem..

[26] Zhao M., Jimenez J. C., Wang C., Rui G., Ma T., Lu C., Rheingold A. L., Li R., Zhu L., Sauvé G. (2022). Monofluorination
of Naphthyls Promotes the Cofacial π–π Stacking
and Increases the Electron Mobility of Non-Planar Zinc­(II) Complexes
of Di­(naphthylethynyl)­azadipyrromethene. J.
Phys. Chem. C.

[27] Knight L., Jimenez J. C., Tran Q., Zhao M., Pugh M. H., Brancel C. D., Zhang H., Li R., Yuan Y., Li Y., Zhu L., Sauvé G. (2024). Tuning the
charge transport properties
of non-planar zinc­(ii) complexes of azadipyrromethenes using solubilizing
groups. Mater. Adv..

[28] Marcus R. A. (1993). Electron
transfer reactions in chemistry: theory and experiment (Nobel lecture). Angew. Chem., Int. Ed..

[29] Chang Y.-C., Chao I. (2010). An Important Key to Design Molecules with Small Internal Reorganization
Energy: Strong Nonbonding Character in Frontier Orbitals. J. Phys. Chem. Lett..

[30] Mas-Torrent M., Hadley P., Bromley S. T., Ribas X., Tarrés J., Mas M., Molins E., Veciana J., Rovira C. (2004). Correlation between
Crystal Structure and Mobility in Organic Field-Effect Transistors
Based on Single Crystals of Tetrathiafulvalene Derivatives. J. Am. Chem. Soc..

[31] Cheung D. L., Troisi A. (2008). Modelling charge transport
in organic semiconductors:
from quantum dynamics to soft matter. Phys.
Chem. Chem. Phys..

[32] Deng W.-Q., Goddard W. A. (2004). Predictions of Hole
Mobilities in Oligoacene Organic
Semiconductors from Quantum Mechanical Calculations †. J. Phys. Chem. B.

[33] Senevirathna W., Daddario C. M., Sauvé G. (2014). Density Functional
Theory Study Predicts
Low Reorganization Energies for Azadipyrromethene-Based Metal Complexes. J. Phys. Chem. Lett..

[34] Gajdos F., Oberhofer H., Dupuis M., Blumberger J. (2013). On the inapplicability
of electron-hopping models for the organic semiconductor phenyl-C61-butyric
acid methyl ester (PCBM). J. Phys. Chem. Lett..

[35] Bredas J.
L., Calbert J. P., da Silva Filho D. A., Cornil J. (2002). Organic semiconductors:
a theoretical characterization of the basic parameters governing charge
transport. Proc. Natl. Acad. Sci. U. S. A..

[36] Huang J., Kertesz M. (2004). Intermolecular transfer integrals for organic molecular
materials: can basis set convergence be achieved?. Chem. Phys. Lett..

[37] Hunter C. A., Sanders J. K. M. (1990). The Nature of
Pi-Pi Interactions. J. Am. Chem. Soc..

[38] Xiao Q., Levine M. S., Iverson B. L. (2026). Rethinking the Terms “pi-Stacking”
and “pi-pi Stacking” Again: A Proposal to Clarify the
Language of Aromatic Interactions. J. Am. Chem.
Soc..

[39] Mas-Torrent M., Rovira C. (2011). Role of molecular order and solid-state structure in
organic field-effect transistors. Chem. Rev..

[40] Mottishaw J. D., Sun H. (2013). Effects of
Aromatic Trifluoromethylation, Fluorination, and Methylation
on Intermolecular π-π Interactions. J. Phys. Chem. A.

[41] Liu H., Kang S., Lee J. Y. (2011). Electronic
Structures and Charge
Transport of Stacked Annelated β-Trithiophenes. J. Phys. Chem. B.

[42] Maiti B., Schubert A., Sarkar S., Bhandari S., Wang K., Li Z., Geva E., Twieg R. J., Dunietz B. D. (2017). Enhancing charge
mobilities in organic semiconductors by selective fluorination: a
design approach based on a quantum mechanical perspective. Chem. Sci..

[43] Hansch C., Leo A., Taft R. W. (1991). A Survey
of Hammett Substituent Constants and Resonance
and Field Parameters. Chem. Rev..

[44] Pejić S., Thomsen A. M., Etheridge F. S., Fernando R., Wang C., Sauvé G. (2018). Fluorination
increases the electron mobility of zinc
azadipyrromethene-based electron acceptors and enhances the performance
of fullerene-free organic solar cells. J. Mater.
Chem. C.

[45] Sworakowski J., Janus K. (2017). On the reliability of determination of energies of HOMO levels in
organic semiconducting polymers from electrochemical measurements. Org. Electron..

[46] Griggs S., Marks A., Bristow H., McCulloch I. (2021). n-Type organic
semiconducting polymers: stability limitations, design considerations
and applications. J. Mater. Chem. C.

[47] Etheridge F. S., Fernando R. J., Pejić S., Zeller M., Sauvé G. (2016). Synthesis
and characterization of fluorinated azadipyrromethene complexes as
acceptors for organic photovoltaics. Beilstein
J. Org. Chem..

[48] Deng W.-Q., Sun L., Huang J.-D., Chai S., Wen S.-H., Han K.-L. (2015). Quantitative
prediction of charge mobilities of π-stacked systems by first-principles
simulation. Nat. Protoc..

[49] Chen H.-Y., Chao I. (2005). Effect of perfluorination
on the charge-transport properties of organic
semiconductors: density functional theory study of perfluorinated
pentacene and sexithiophene. Chem. Phys. Lett..

[50] Chen W.-C., Chao I. (2014). Molecular Orbital-Based Design of
π-Conjugated Organic Materials
with Small Internal Reorganization Energy: Generation of Nonbonding
Character in Frontier Orbitals. J. Phys. Chem.
C.

[51] Knight L. R., Tran Q. D., Raufman D., Paparella A. A., Brancel C. D., Jadhav N., Zou Q., Rodrigues V., Jimenez J. C., Wu S., Zhu L., Sauve G. (2025). Side chain
engineering of near-IR aza-BODIPY dyes enable processable films with
high hole mobility in diodes. J. Phys. Chem.
C.

[52] Apex5 v2023.9–2, SAINT V8.40B; Bruker AXS Inc.: Madison, WI, USA, 2023.

[53] Krause L., Herbst-Irmer R., Sheldrick G. M., Stalke D. (2015). Comparison of Silver
and Molybdenum Microfocus X-ray Sources for Single-Crystal Structure
Determination. J. Appl. Crystallogr..

[54] SHELXTL Suite of Programs, Version 6.14, 2000–2003; Bruker Advanced X-ray Solutions; Bruker AXS Inc.; Madison, WI, USA, 2003.

[55] Sheldrick G. M. (2008). A Short
History of SHELX. Acta Crystallogr., Sect. A.

[56] Sheldrick G. M. (2015). SHELXT
– Integrated space-group and crystal-structure determination. Acta Crystallogr., Sect. A: Found. Adv..

[57] SHELXL2019; University of Göttingen: Germany, 2019.

[58] Sheldrick G. (2015). Crystal Structure
Refinement with SHELXL. Acta Crystallogr., Sect.
C: Struct. Chem..

[59] Hubschle C. B., Sheldrick G. M., Dittrich B. (2011). ShelXle: a Qt graphical
user interface
for SHELXL. J. Appl. Crystallogr..

[60] Klimka̅ns A., Larsson S. (1994). Reorganization energies
in benzene, naphthalene, and
anthracene. Chem. Phys..

[61] Nelsen S. F., Blackstock S. C., Kim Y. (1987). Estimation of inner shell Marcus
terms for amino nitrogen compounds by molecular orbital calculations. J. Am. Chem. Soc..

[62] Nelsen S. F., Yunta M. J. R. (1994). Estimation of marcus λ for p-phenylenediamines
from the optical spectrum of a dimeric derivative. J. Phys. Org. Chem..

[63] SCM. Electron and Hole Mobilities in Organic Electronics: Charge Transfer Integrals; 2026, https://www.scm.com/doc/Tutorials/ElectronicTransport/ElectronAndHoleMobilities.html.

[64] Coropceanu V., Cornil J., da Silva
Filho D. A., Olivier Y., Silbey R., Bredas J.-L. (2007). Charge
transport in organic semiconductors. Chem. Rev..

