# Supplementary material for: Fluorinated Arylethynyl Substituents in Zn(II) Complexes of Azadipyrromethene Guide Self-Assembly in Crystals
Source: Inorg Chem. 2026 Oct 15;65(43):25389–401. doi: 10.1021/acs.inorgchem.6c03689 (PMC13733264; doi:10.1021/acs.inorgchem.6c03689)
Supplement: Supplementary file 1 [file ic6c03689_si_001.pdf]

Supporting Information

Fluorinated arylethynyl substituents in Zn(II) complexes of azadipyrromethene guide self-assembly in crystals

David A. Raufman[1], Neha Jadhav[1], Shashika A. Arachchige[1], Lexi R. Knight[1], Jayvic C. Jimenez[2], Genevieve Sauve[1,]*

[1]Department of Chemistry, Case Western Reserve University, 10900 Euclid Avenue, Cleveland, OH 44106, USA.

[2]Lawrence Livermore National Laboratory, Livermore, California 94550, USA.

*Corresponding author email address: gxs244@case.edu

Tables of Contents

NMR Spectra S3
MALDI-TOF mass spectra S16
Crystallographic Information S19
Crystal packing and calculated overlap integrals S24
Computational data S33

NMR Spectra

^{1}H NMR (500 MHz, $CDCl_3$) δ 7.40 – 7.34 (m, 2H), 7.21 – 7.15 (m, 3H), 1.59 – 1.48 (m, 6H), 1.34 – 1.25 (m, 6H), 1.03 – 0.92 (m, 6H), 0.85 (t, *J* = 7.3 Hz, 9H).

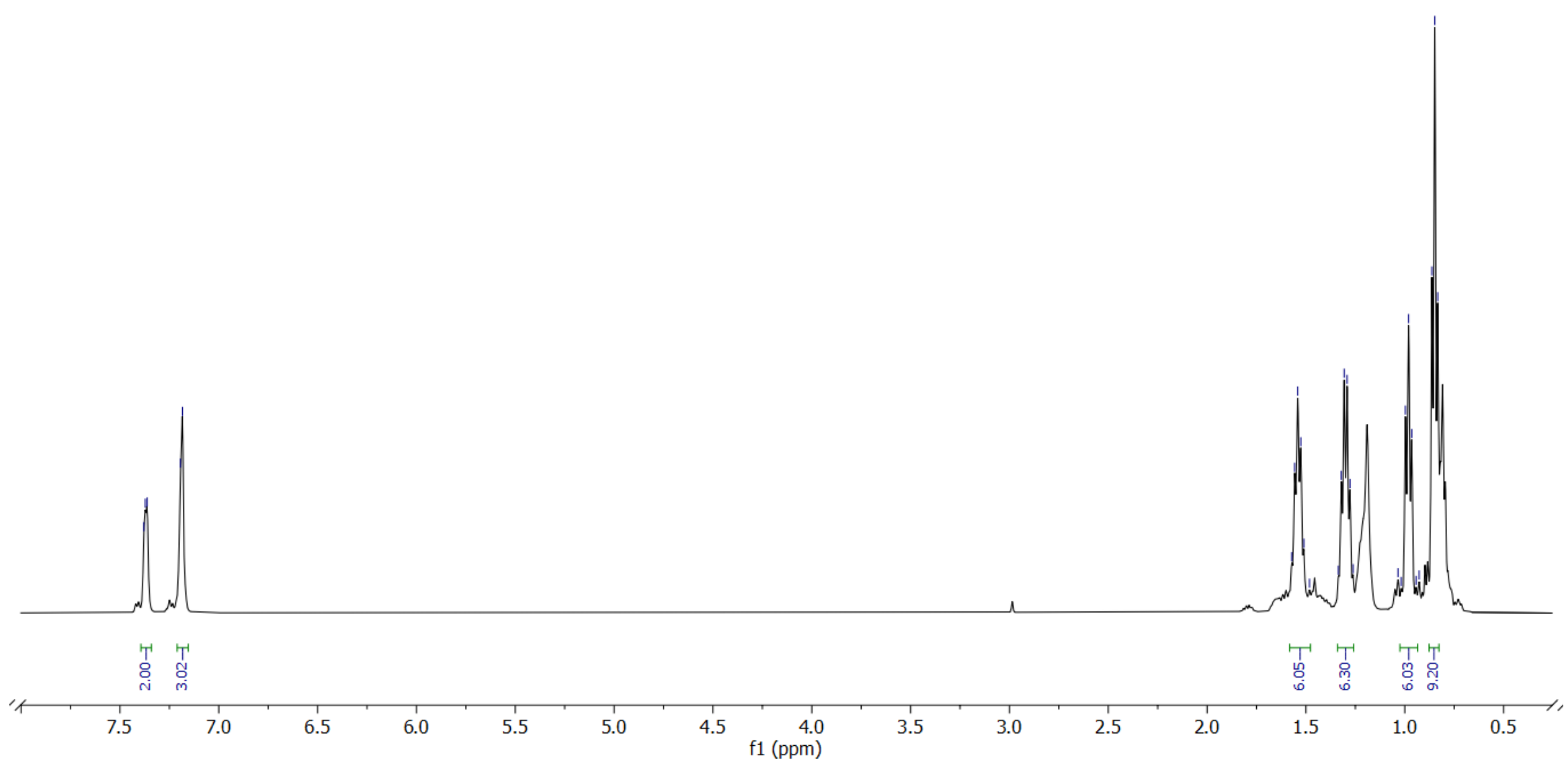

Figure S1: ^{1}H NMR spectrum of tributyl(phenylethynyl)stannane. The spectrum shows the presence of starting material (small aromatic peaks and ethynyl C-H around 3 ppm) and residual hexanes (CH3 ~0.7 ppm and CH2 ~1.2 ppm). The product was not further purified because neither of these impurities are expected to affect the next step.

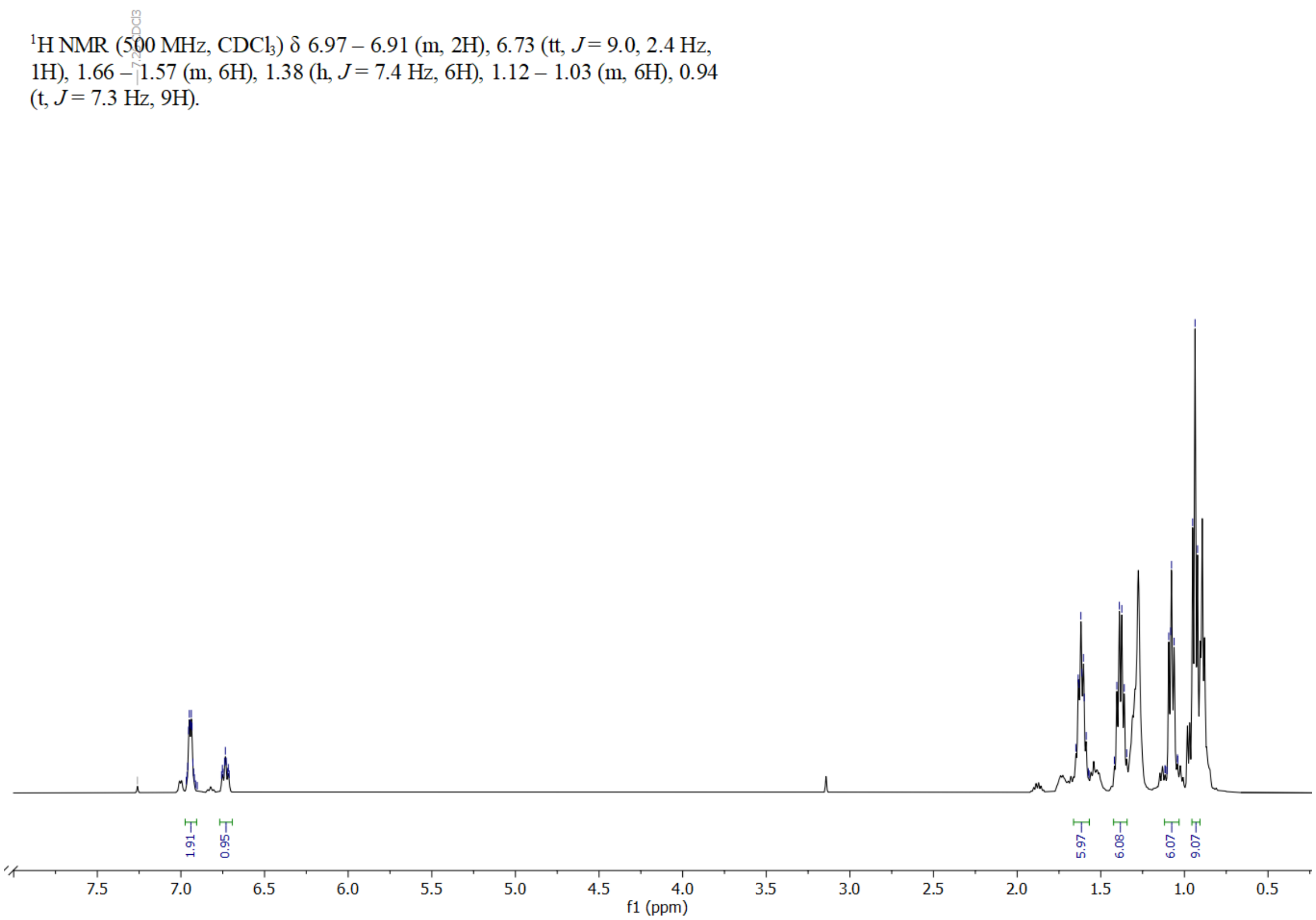

Figure S2: ^{1}H NMR spectrum of tributyl((3,5-difluorophenyl)ethynyl)stannane. The spectrum shows the presence of starting material (small aromatic peaks and ethynyl C-H around 3.2 ppm) and residual hexanes.

^{1}H NMR (500 MHz, $CDCl_3$) δ 7.85 (s, 2H), 7.75 (s, 1H), 1.68 – 1.55 (m, 6H), 1.39 (h, *J* = 7.4 Hz, 6H), 1.15 – 1.05 (m, 6H), 0.94 (t, *J* = 7.4 Hz, 9H).

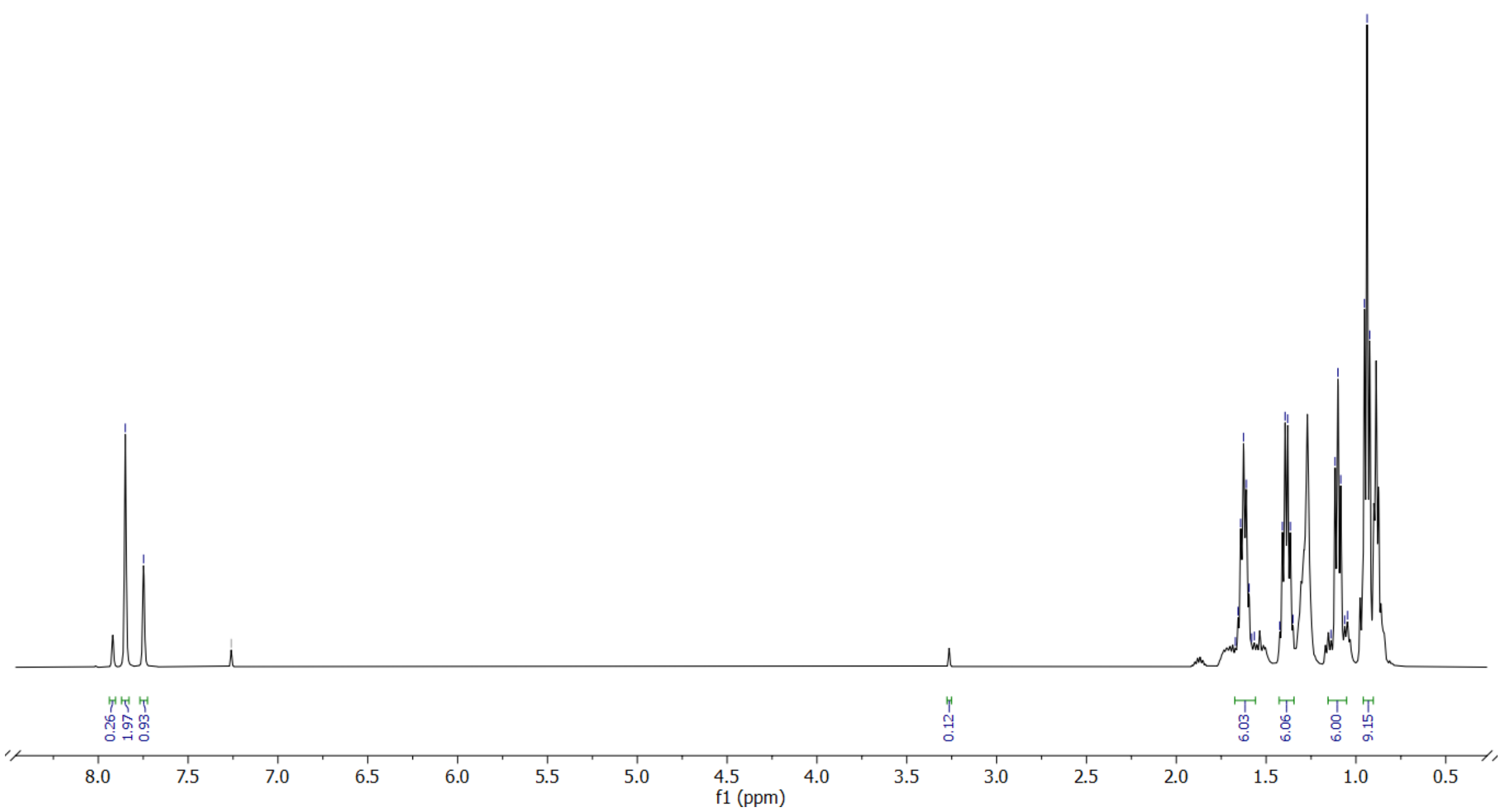

Figure S3: ^{1}H NMR spectrum of ((3,5-bis(trifluoromethyl)phenyl)ethynyl)tributylstannane. The spectrum shows the presence of starting material (ethynyl C-H around 3 .5 ppm and aromatic peak around 7.9 pmm) and residual hexanes.

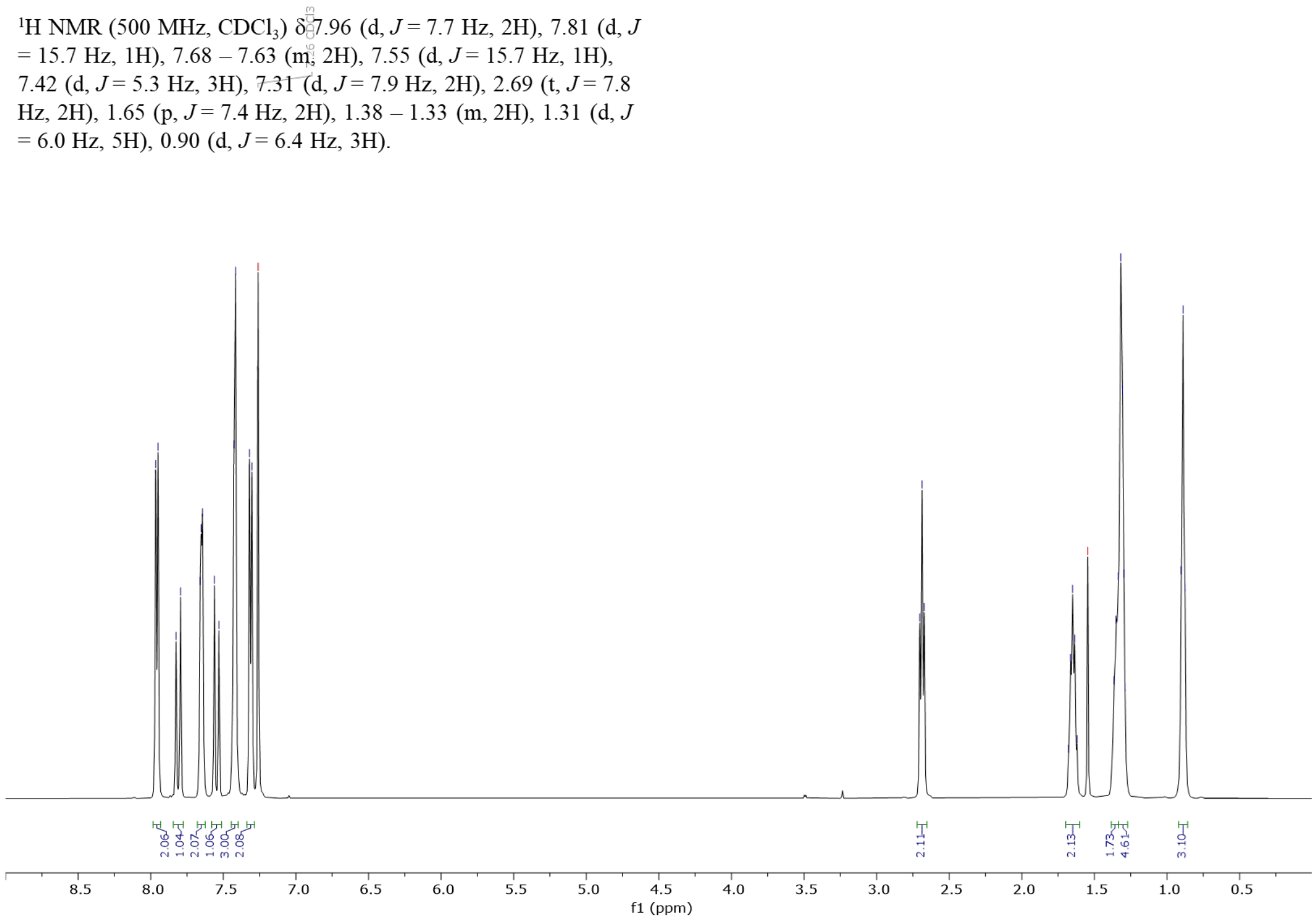

Figure S4: ^{1}H NMR spectrum of 4-hexylchalcone.

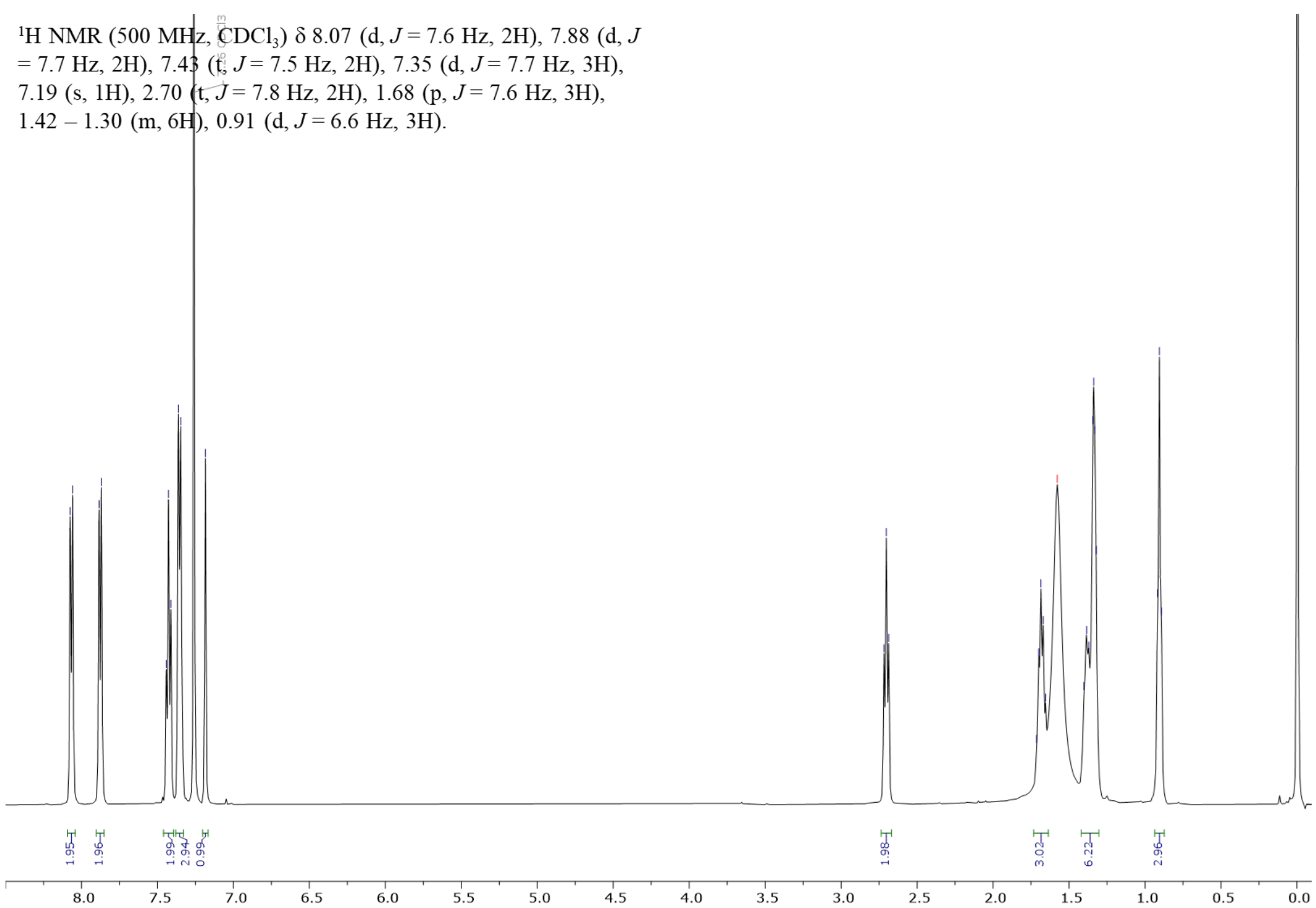

Figure S5: ^{1}H NMR spectrum of phexADP.

^1H NMR (500 MHz, $CDCl_3$) δ 7.91 (d, *J* = 7.8 Hz, 1H), 7.70 (d, *J* = 7.0 Hz, 1H), 7.40 – 7.30 (m, 3H), 2.70 (t, *J* = 7.8 Hz, 1H), 1.68 (p, *J* = 7.5 Hz, 1H), 1.40 (d, *J* = 7.6 Hz, 1H), 1.34 (d, *J* = 6.3 Hz, 2H), 0.94 – 0.85 (m, 2H).

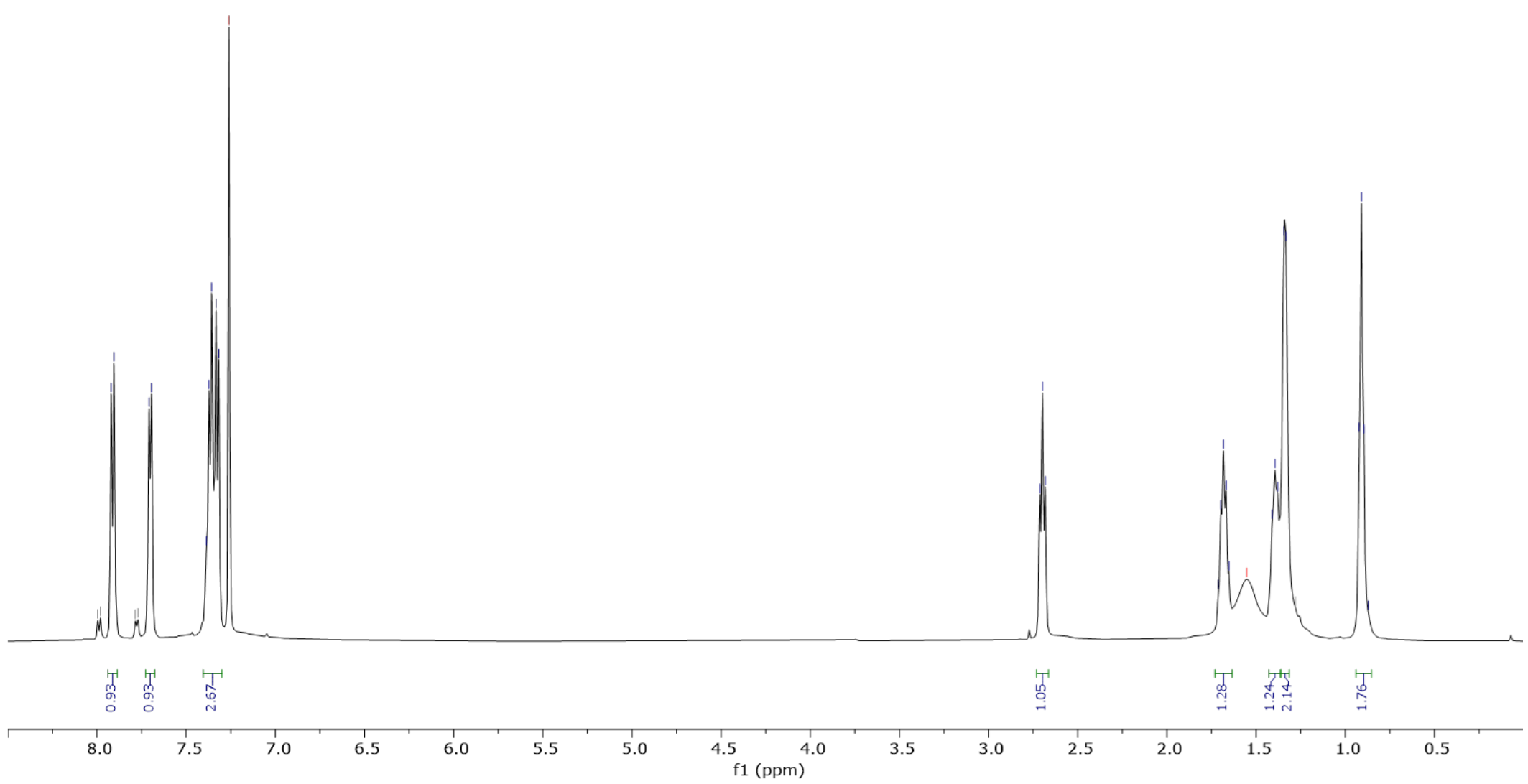

Figure S6: ^1H NMR spectrum of phexADPI$_2$.

^{1}H NMR (500 MHz, $CDCl_3$) δ 8.28 (d, J = 7.9 Hz, 4H), 8.20 (d, J = 7.7 Hz, 4H), 7.49 (d, J = 7.2 Hz, 4H), 7.47 – 7.39 (m, 6H), 7.39 – 7.34 (m, 10H), 2.73 (t, J = 7.9 Hz, 4H), 1.71 (p, J = 7.8 Hz, 4H), 1.37 – 1.35 (m, 12H), 0.93 – 0.92 (m, 6H).

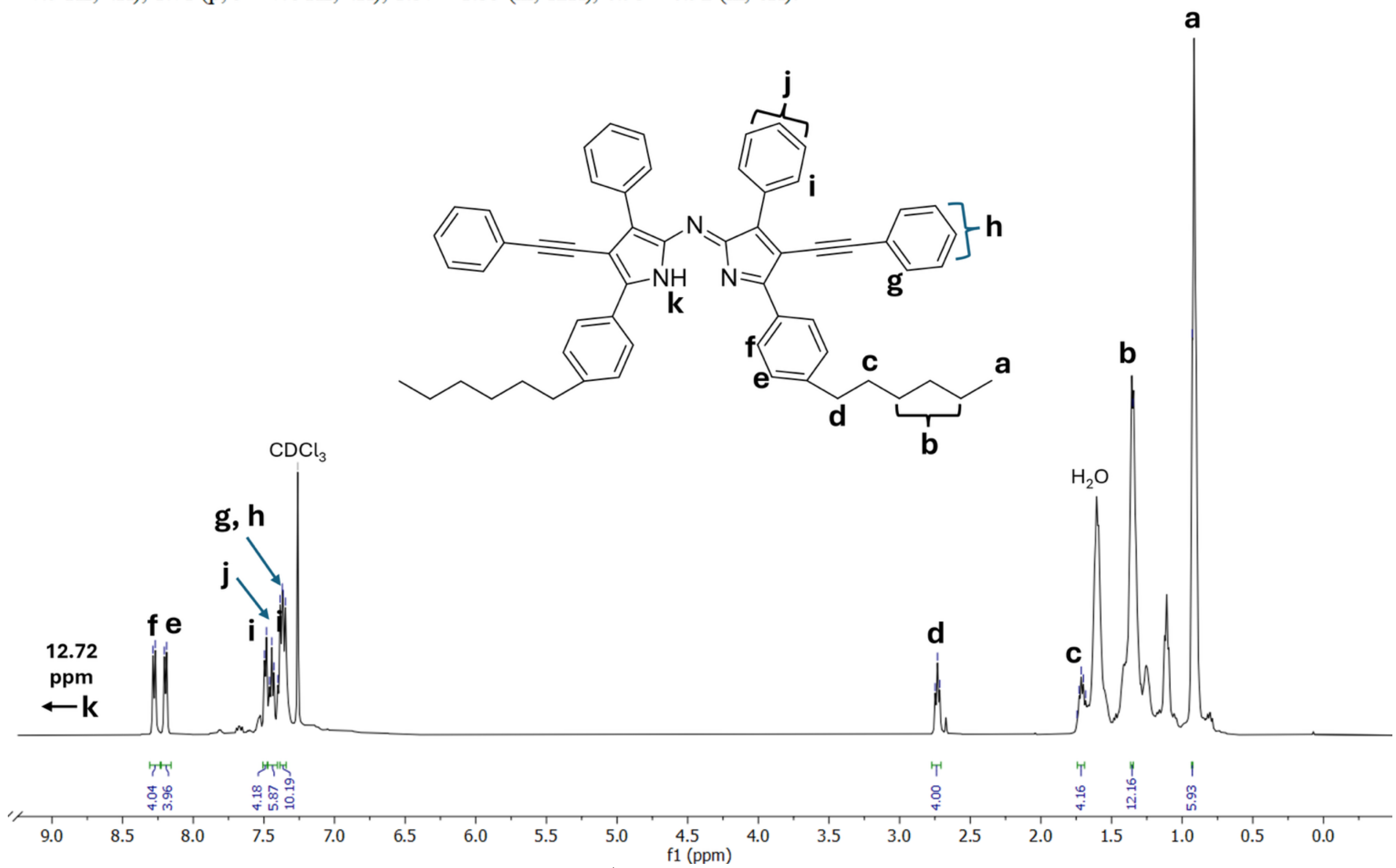

Figure S7: ^{1}H NMR spectrum of L1.

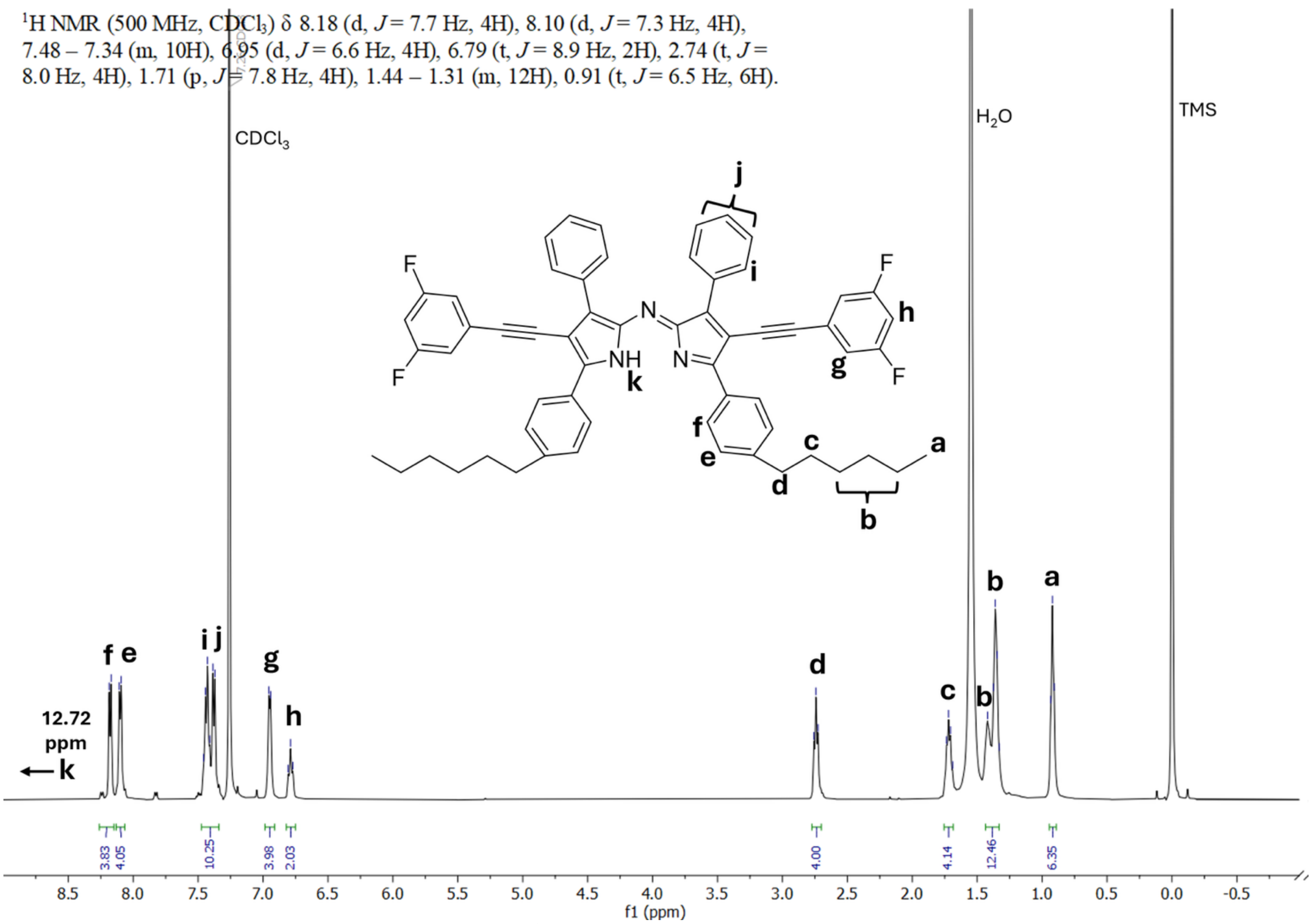

Figure S8: ^{1}H NMR spectrum of L1-*m*-2F.

^{19}F NMR (471 MHz, $CDCl_3$) δ -109.48.

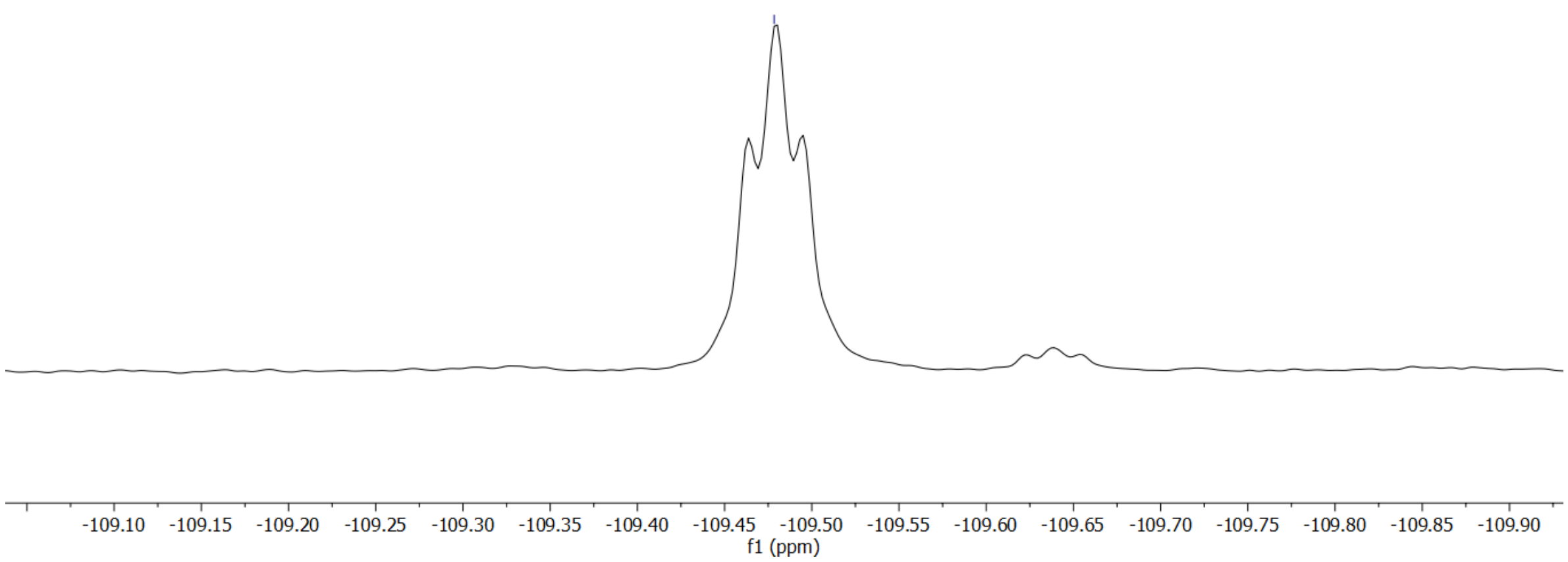

Figure S9: ^{19}F NMR spectrum of L1-*m*-2F.

^{1}H NMR (500 MHz, $CDCl_3$) δ 8.08 – 8.03 (m, 8H), 7.62 (d, *J* = 7.7 Hz, 8H), 7.45 – 7.40 (m, 12H), 7.37 (d, *J* = 7.4 Hz, 8H), 7.33 – 7.27 (m, 12H), 7.00 (d, *J* = 7.7 Hz, 8H), 2.34 (t, *J* = 8.2 Hz, 8H), 1.36 (p, *J* = 7.4 Hz, 8H), 1.28 – 1.07 (m, 24H), 0.85 (t, *J* = 7.3 Hz, 12H).

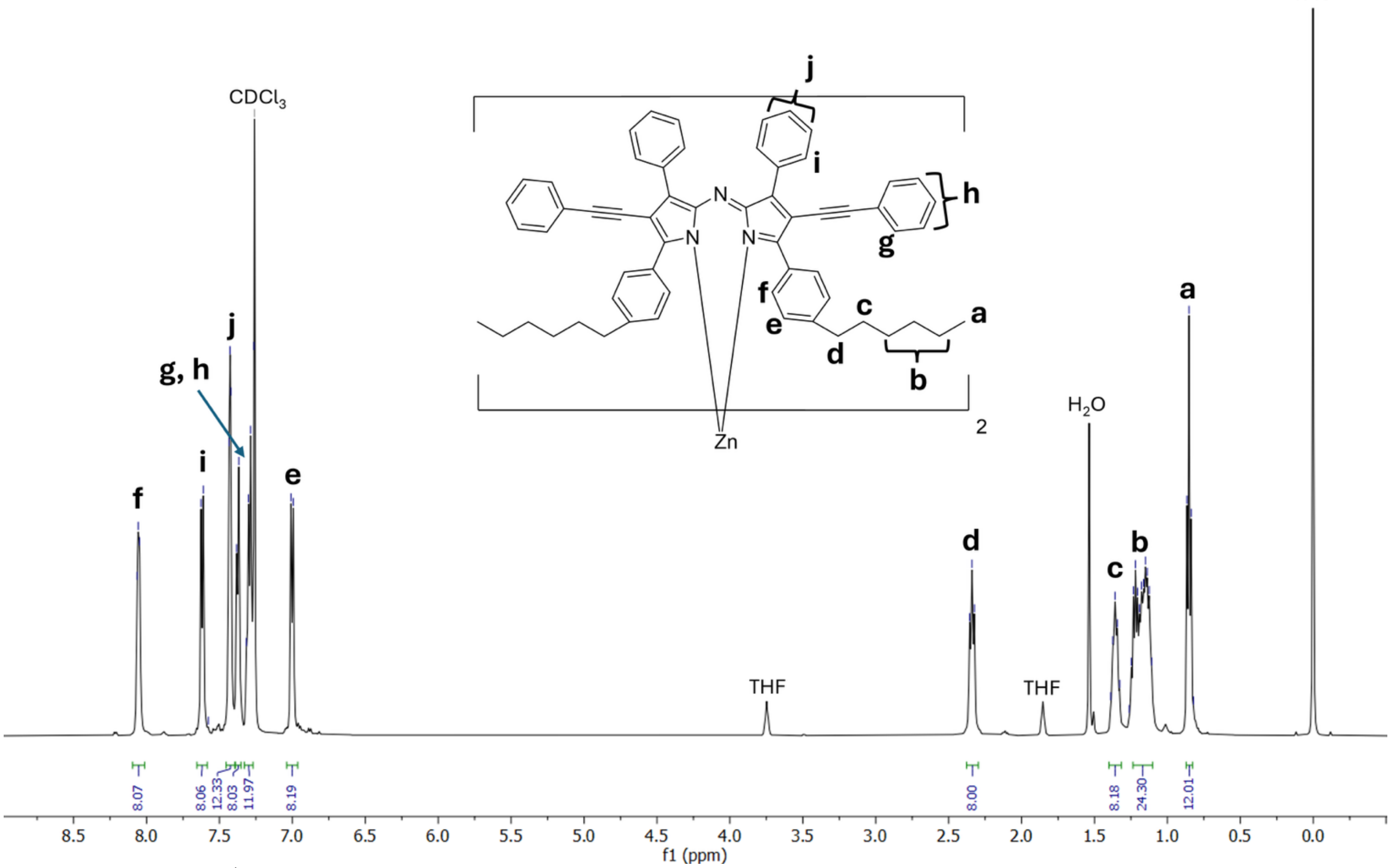

Figure S10: ^{1}H NMR spectrum of $Zn(L1)_2$. The peaks at 1.85 ppm and 3.75 ppm correspond to residual THF, which gets trapped between the ligands in the solid.

^{13}C NMR (126 MHz, $CDCl_3$) δ 162.07, 146.57, 145.61, 145.00, 133.43, 131.18, 131.08, 129.73, 128.43, 128.01, 127.93, 127.69, 124.20, 111.99, 96.71, 85.95, 35.93, 31.77, 30.93, 29.50, 22.84, 14.31.

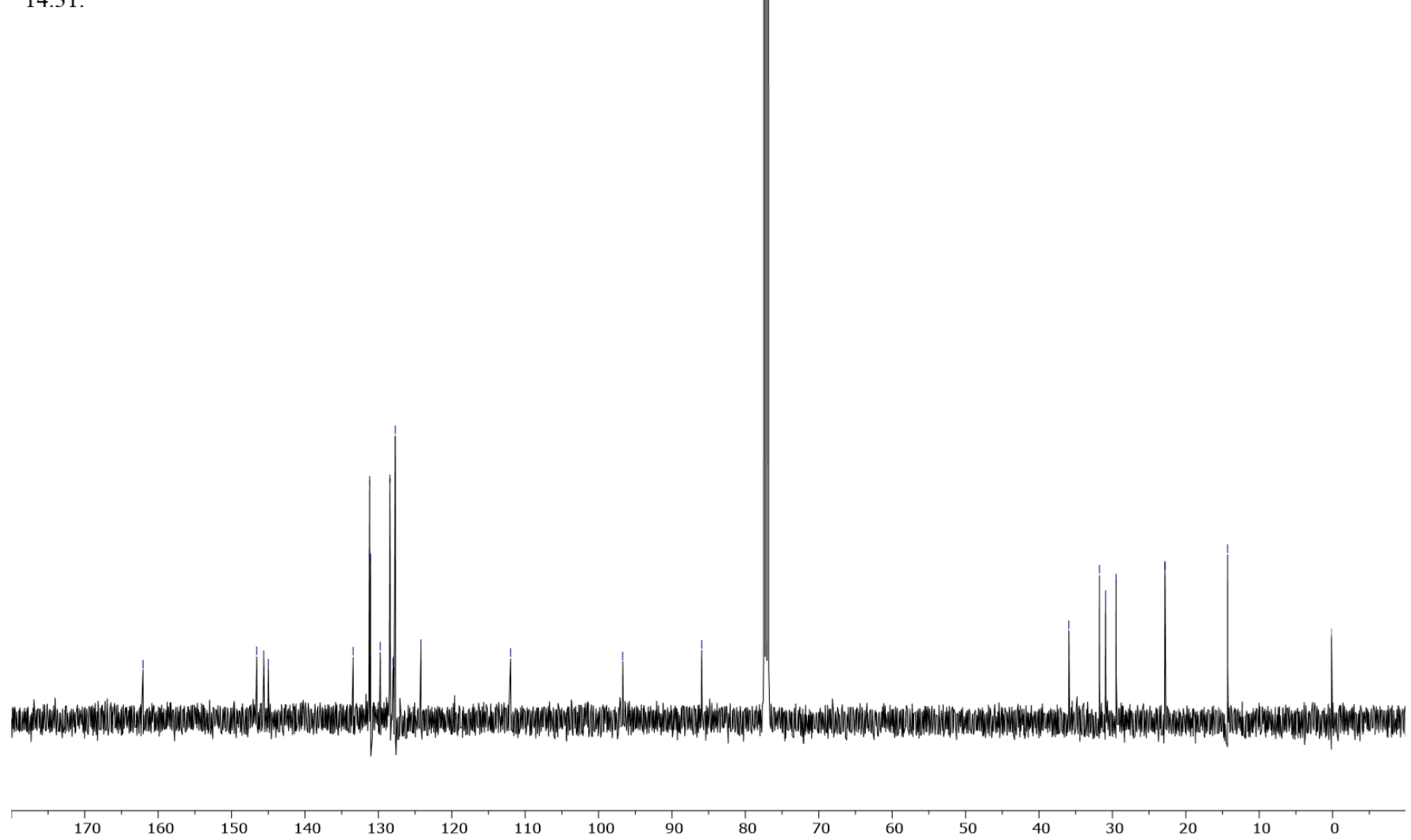

Figure S11: ^{13}C NMR spectrum of $Zn(L1)_2$.

^{1}H NMR (500 MHz, $CDCl_3$) δ 7.97 (d, *J* = 6.8 Hz, 8H), 7.55 (d, *J* = 7.7 Hz, 8H), 7.47 – 7.42 (m, 12H), 7.02 (d, *J* = 7.6 Hz, 8H), 6.85 (d, *J* = 6.5 Hz, 8H), 6.74 (t, *J* = 9.1 Hz, 4H), 2.35 (t, *J* = 8.2 Hz, 8H), 1.35 (p, *J* = 7.5 Hz, 8H), 1.28 – 1.07 (m, 24H), 0.85 (t, *J* = 7.3 Hz, 12H).

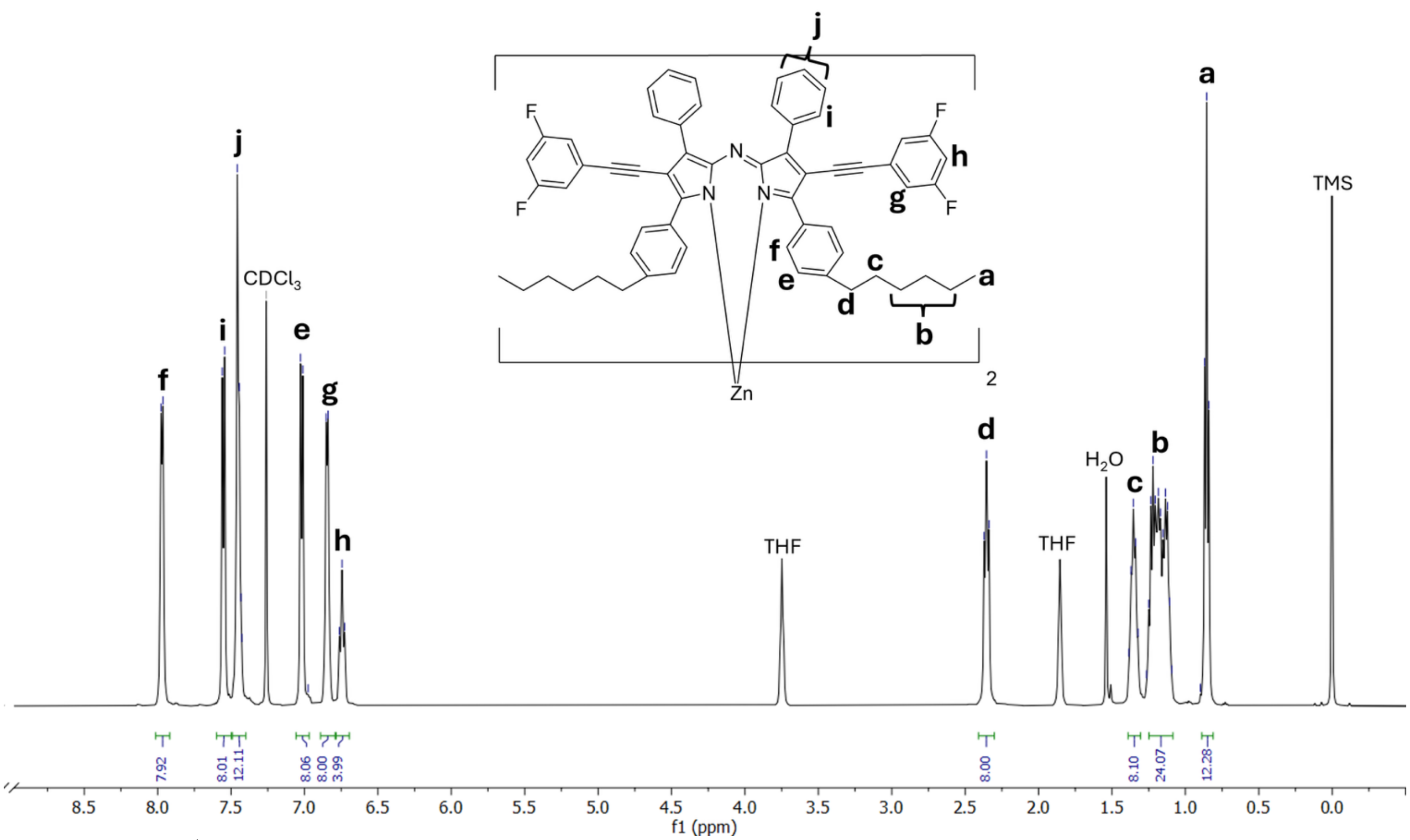

Figure S12: ^{1}H NMR spectrum of Zn(L1-*m*-2F)$_2$. The peaks at 1.85 ppm and 3.75 ppm correspond to residual THF, which gets trapped between the ligands.

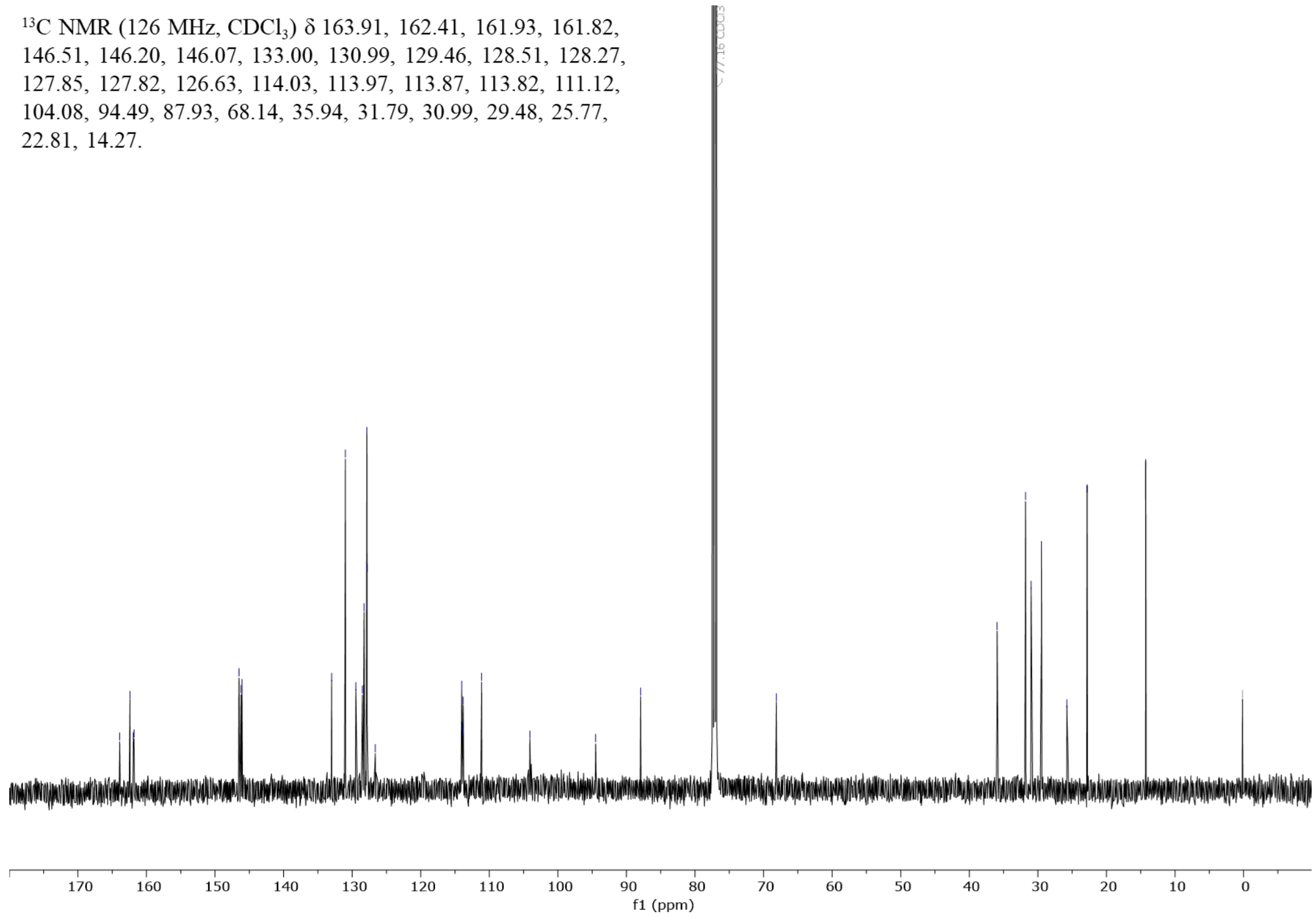

Figure S13: ^{13}C NMR spectrum of Zn(L1-*m*-2F)$_2$.

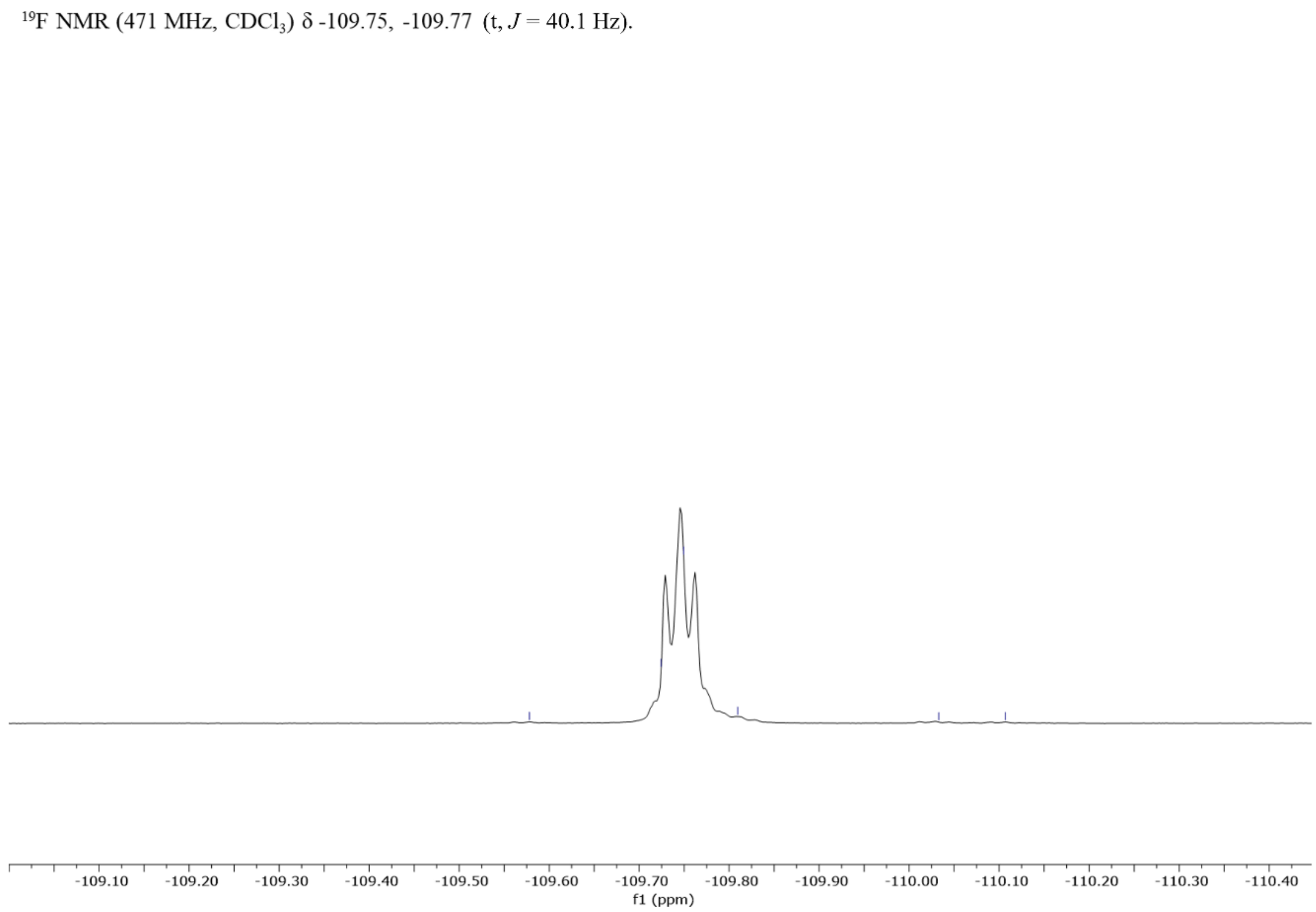

Figure S14: ^{19}F NMR spectrum of Zn(L1-*m*-2F)$_2$.

^{1}H NMR (500 MHz, $CDCl_3$) δ 7.97 (d, *J* = 7.1 Hz, 8H), 7.74 (s, 4H), 7.72 (s, 8H), 7.57 (d, *J* = 7.7 Hz, 8H), 7.54 – 7.44 (m, 12H), 7.05 (d, *J* = 7.6 Hz, 8H), 2.38 (t, *J* = 8.1 Hz, 8H), 1.37 (p, *J* = 7.6 Hz, 8H), 1.27 – 1.07 (m, 24H), 0.84 (t, *J* = 7.2 Hz, 12H).

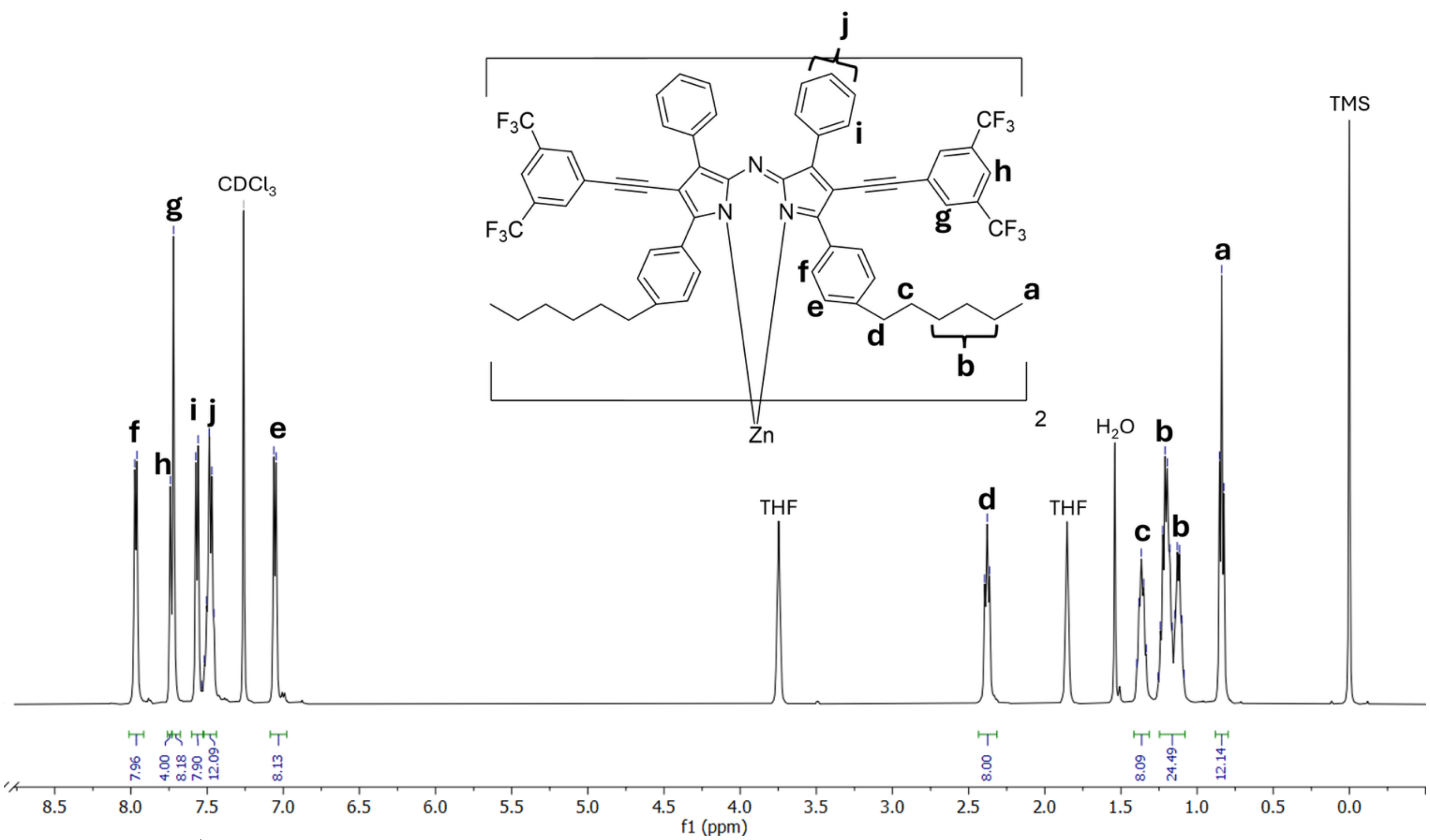

Figure S15: ^{1}H NMR spectrum of Zn(L1-*m*-2CF_3)$_2$. The peaks at 1.85 ppm and 3.75 ppm correspond to residual THF, which gets trapped between the ligands.

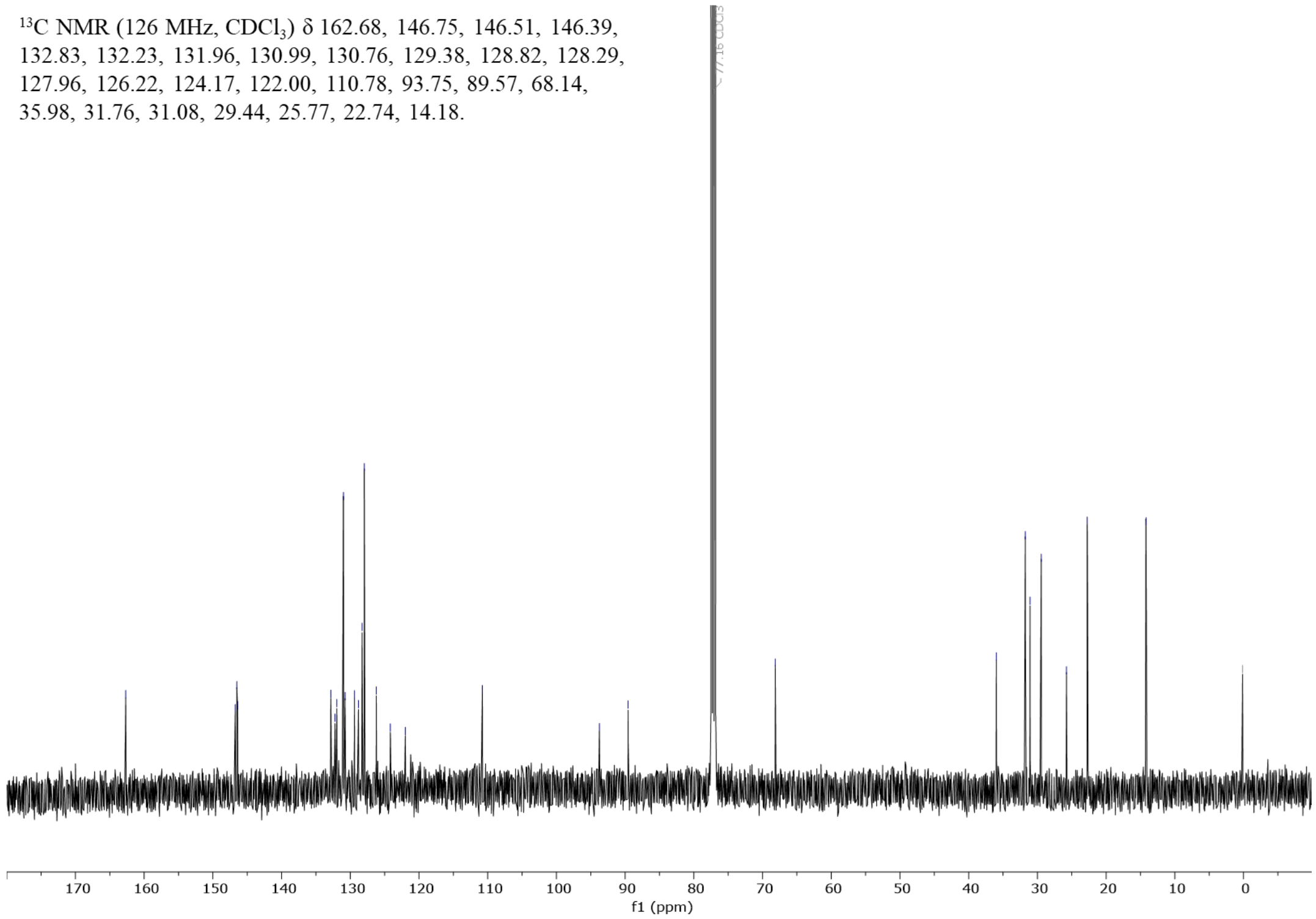

Figure S16: ^{13}C NMR spectrum of Zn(L1-*m*-2CF_3)$_2$.

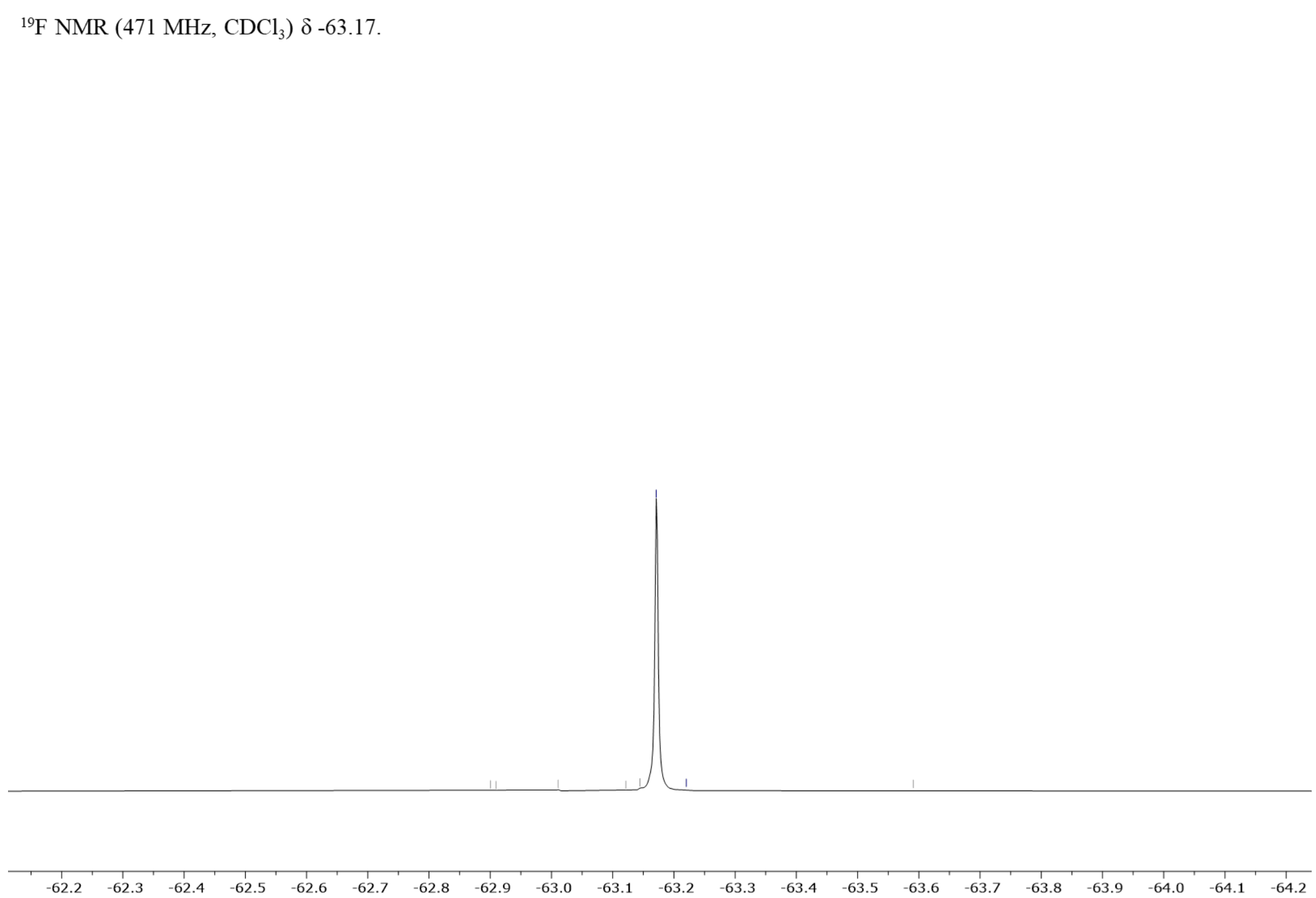

Figure S17: ^{19}F NMR spectrum of Zn(L1-*m*-2CF_3)$_2$.

MALDI-TOF mass spectra

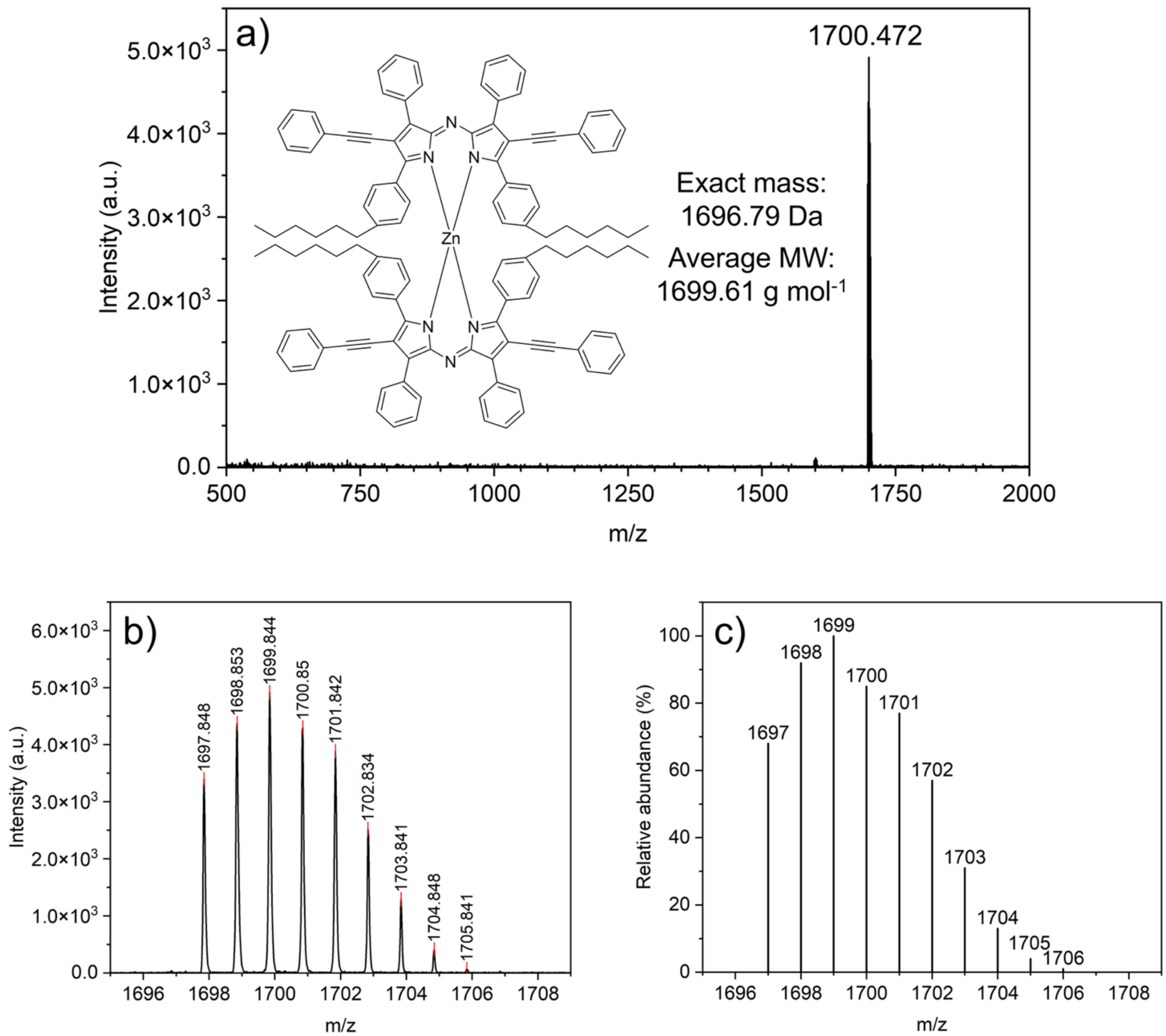

Figure S18: a) MALDI-TOF mass spectrum for $Zn(L1)_2$. The peak label is the centroid mass. b) Zoomed-in part of the mass spectrum showing the isotope pattern. c) Calculated isotope pattern.

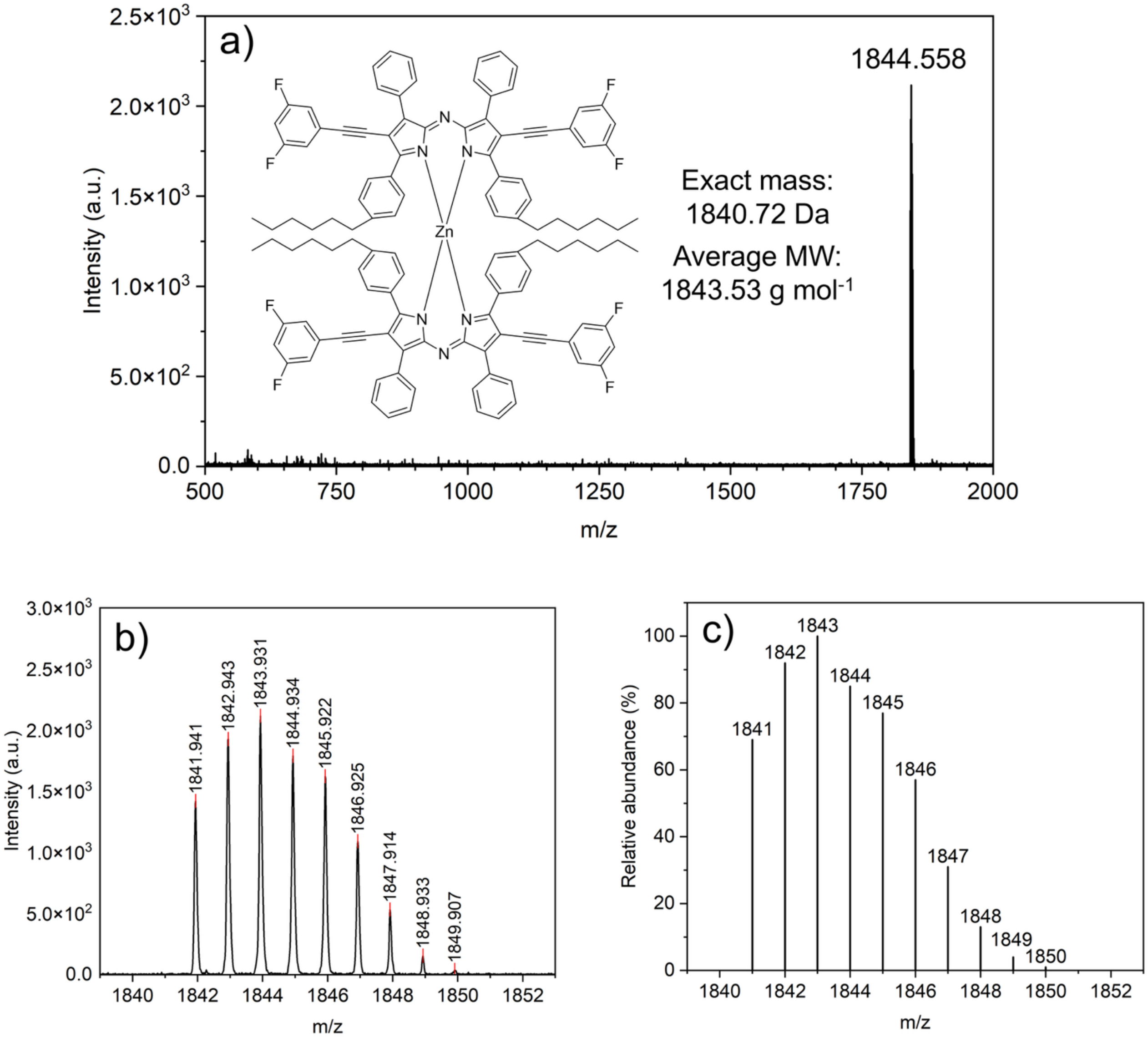

Figure S19: a) MALDI-TOF mass spectrum for Zn(L1-*m*-2F)$_2$. The peak label is the centroid mass. b) Zoomed-in part of the mass spectrum showing the isotope pattern. c) Calculated isotope pattern.

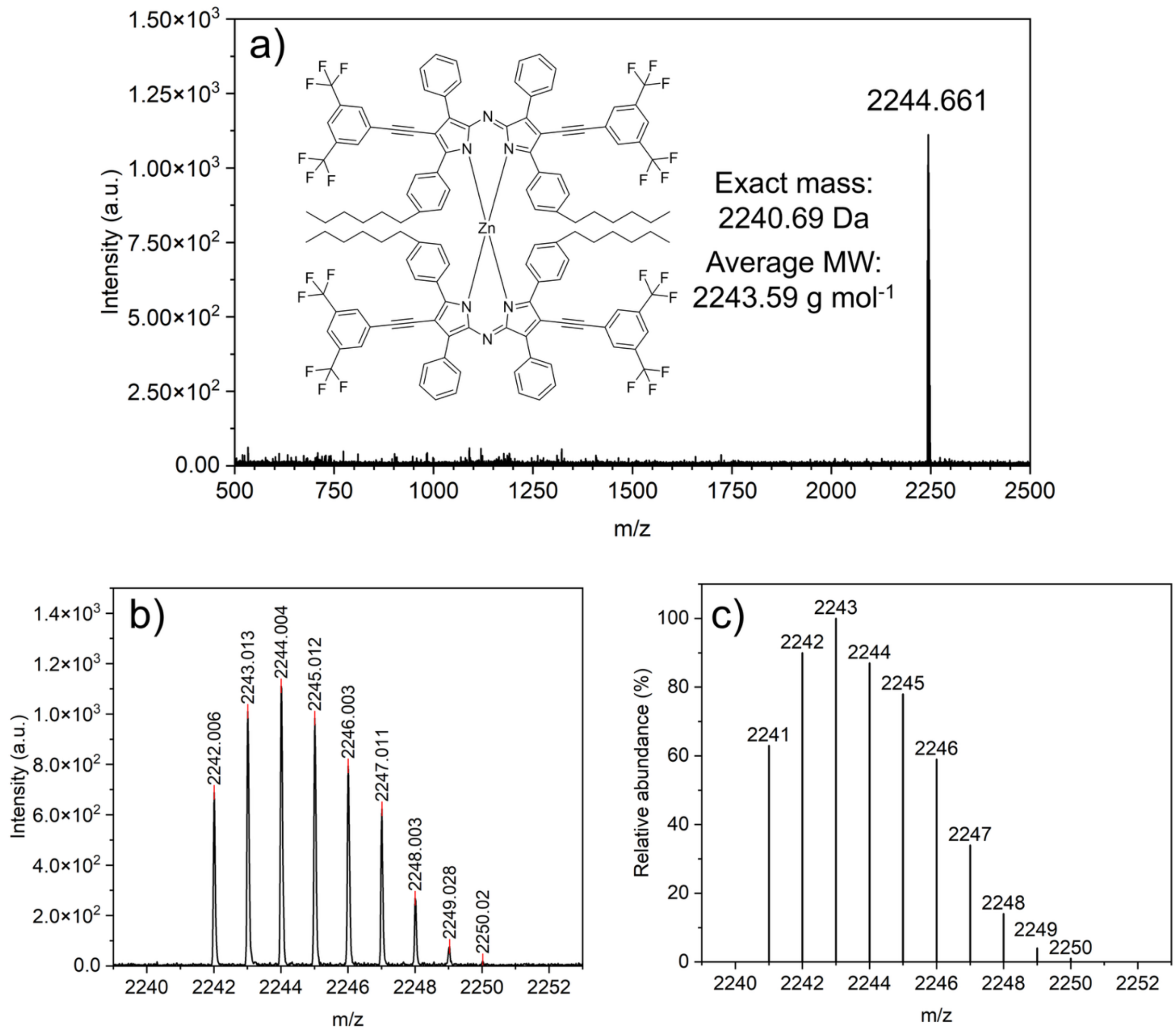

Figure S20: a) MALDI-TOF mass spectrum for Zn(L1-*m*-2CF$_3$)$_2$. The peak label is the centroid mass. b) Zoomed-in part of the mass spectrum showing the isotope pattern. c) Calculated isotope pattern.

Crystallographic Information

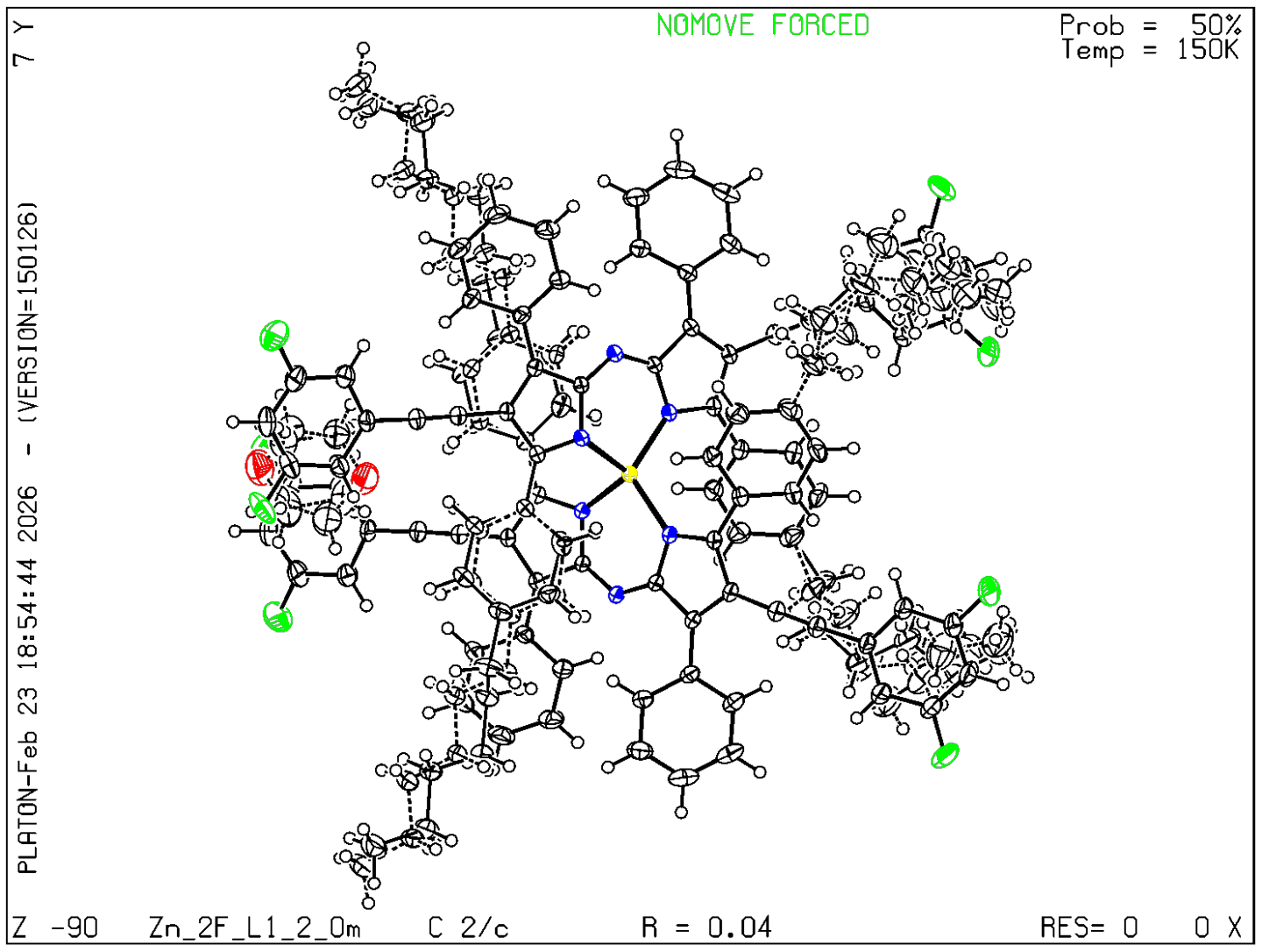

Figure S21: Crystal structure of Zn(L1-*m*-2F)$_2$ obtained from FinalCIF.

Table S1: Crystal data and structure refinement for Zn(L1-*m*-2F)$_2$

CCDC number	2533094
Empirical formula	$C_{124}H_{108}F_8N_6OZn$
Formula weight	1915.53
Temperature [K]	150(2)
Crystal system	monoclinic
Space group (number)	$C2/c$ (15)
a [Å]	33.409(6)
b [Å]	17.521(4)
c [Å]	20.474(4)
α [°]	90
β [°]	124.649(9)
γ [°]	90
Volume [Å^3]	9859(4)
Z	4

ρ_{calc} [gcm^{-3}]	1.291
μ [mm^{-1}]	0.321
F(000)	4016
Crystal size [mm^3]	0.390×0.500×0.520
Crystal colour	red
Crystal shape	block
Radiation	MoK_α (λ=0.71073 Å)
2θ range [°]	4.19 to 66.51 (0.65 Å)
Index ranges	$-51 \le h \le 51$<br>$-26 \le k \le 26$<br>$-31 \le l \le 31$
Reflections collected	190885
Independent reflections	18890<br>R_{int} = 0.0396<br>R_{sigma} = 0.0206
Completeness to θ = 25.242°	100.0
Data / Restraints / Parameters	18890 / 1065 / 916
Absorption correction T_{min}/T_{max} (method)	0.6784 / 0.7465 (multi-scan)
Goodness-of-fit on F^2	1.024
Final R indexes [$I \geq 2\sigma(I)$]	R_1 = 0.0389<br>wR_2 = 0.1079
Final R indexes [all data]	R_1 = 0.0514<br>wR_2 = 0.1171
Largest peak/hole [eÅ^{-3}]	0.44/−0.40

Refinement details for Zn(L1-*m*-2F)$_2$

The alkyl chains in this structure are disordered. The chain of C43 to C48 was refined as three-fold disordered. The chain of C55 to C60 was refined as two-fold disordered, and the adjacent phenylene ring was included in the disorder (the ipso C atom was excluded). Equivalent disordered moieties were restrained to have similar geometries. U_{ij} components of ADPs for disordered atoms closer to each other than 2.0 Å were restrained to be similar. Subject to these conditions the occupancy ratio refined to 0.639(2) to 0.227(2) to 0.134(2) for C43 to C48 and 0.604(3) to 0.396(3) for C55 to C60. A solvate THF molecule is four-fold disordered. It is located on a two-fold axis, inducing two-fold disorder, and in addition disordered by general symmetry (flipping the molecule to make the O atom point in the opposite direction). The two crystallographically unique disordered moieties were restrained to have similar geometries, and chemically equivalent bond distances and angles to be similar within the major moiety. U_{ij} components of ADPs for disordered atoms closer to each other than 2.0 Å were restrained to be similar. Subject to these conditions the occupancy ratio refined to two times 0.277(4) to two times 0.223(4).

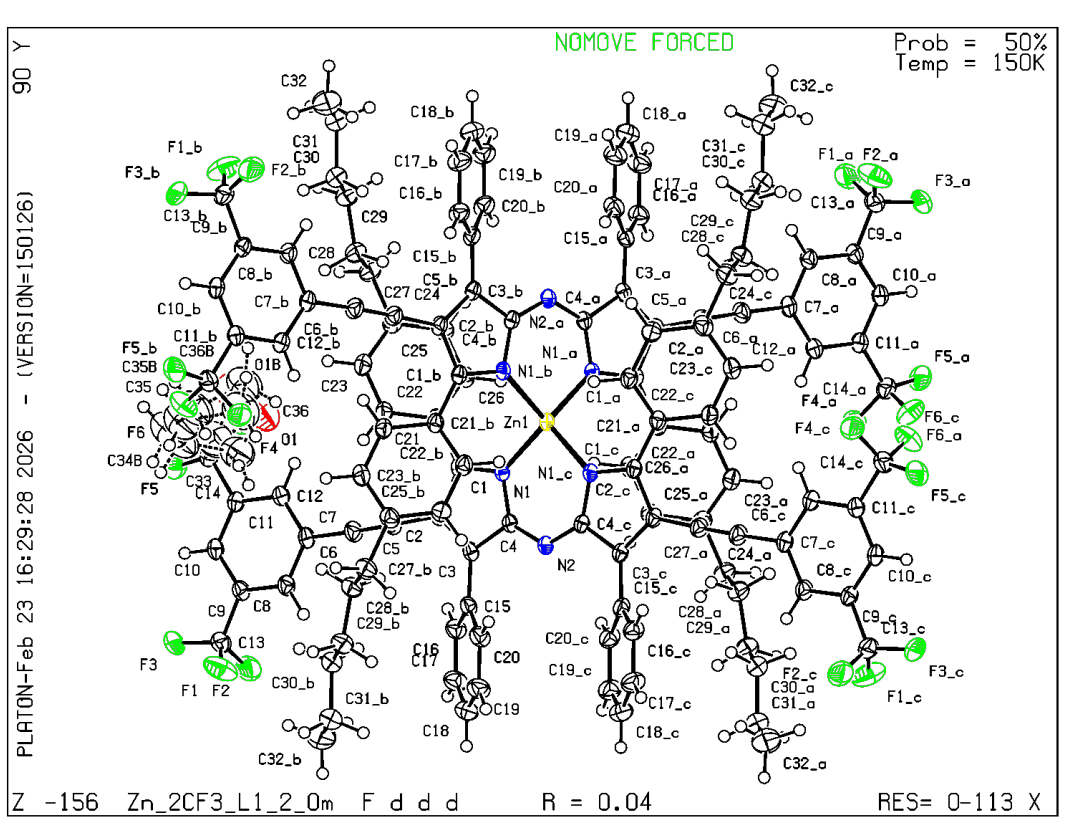

Figure S22: Crystal structure of Zn(L1-*m*-2CF_3)$_2$ generated by FinalCIF.

Table S2: Crystal data and structure refinement for Zn(L1-*m*-2CF_3)$_2$.

CCDC number	2533095
Empirical formula	$C_{136}H_{116}F_{24}N_6O_2Zn$
Formula weight	2387.71
Temperature [K]	150(2)
Crystal system	orthorhombic
Space group (number)	$Fddd$ (70)
a [Å]	22.520(7)
b [Å]	24.765(4)
c [Å]	42.267(9)
α [°]	90
β [°]	90
γ [°]	90
Volume [Å^3]	23573(10)
Z	8
ρ_{calc} [gcm^{-3}]	1.346
μ [mm^{-1}]	0.303
F(000)	9888
Crystal size [mm^3]	0.330×0.380×0.420
Crystal colour	red
Crystal shape	block
Radiation	MoK_α
	(λ=0.71073 Å)
2θ range [°]	4.89 to 66.36
	(0.65 Å)
Index ranges	$-34 \leq h \leq 34$
	$-38 \leq k \leq 36$
	$-62 \leq l \leq 65$
Reflections collected	210619

Independent reflections	11263<br>R_{int} = 0.0483<br>R_{sigma} = 0.0198
Completeness to θ = 25.242°	99.9
Data / Restraints / Parameters	11263 / 226 / 452
Absorption correction T_{min}/T_{max} (method)	0.7034 / 0.7465 (multi-scan)
Goodness-of-fit on F^2	1.048
Final *R* indexes [$I \geq 2\sigma(I)$]	R_1 = 0.0406<br>wR_2 = 0.1068
Final *R* indexes [all data]	R_1 = 0.0581<br>wR_2 = 0.1199
Largest peak/hole [eÅ^{-3}]	0.46/−0.39

Refinement details for Zn(L1-*m*-2CF$_3$)$_2$

A solvate THF molecule is four-fold disordered. It is located on a two-fold axis, inducing two-fold disorder, and in addition disordered by general symmetry. The two crystallographically unique disordered moieties were restrained to have similar geometries, and chemically equivalent bond distances and angles to be similar within the major moiety. The distances between the alpha C atoms were restrained to be at least 2.25(2) Å. U_{ij} components of ADPs for disordered atoms closer to each other than 2.0 Å were restrained to be similar. Subject to these conditions the occupancy ratio refined to two times 0.254(4) to two times 0.246(4).

Crystal packing and calculated overlap integrals

Table S3: Crystal packing of nearest pairs to the selected $Zn(L1)_2$ molecule in red. All nearest pairs with overlap over 0.1 meV are listed. The direction from the red molecule, the main π-π stacking interaction(s), and the hole and electron overlap integrals (in meV) are included.

$Zn(L1)_2$

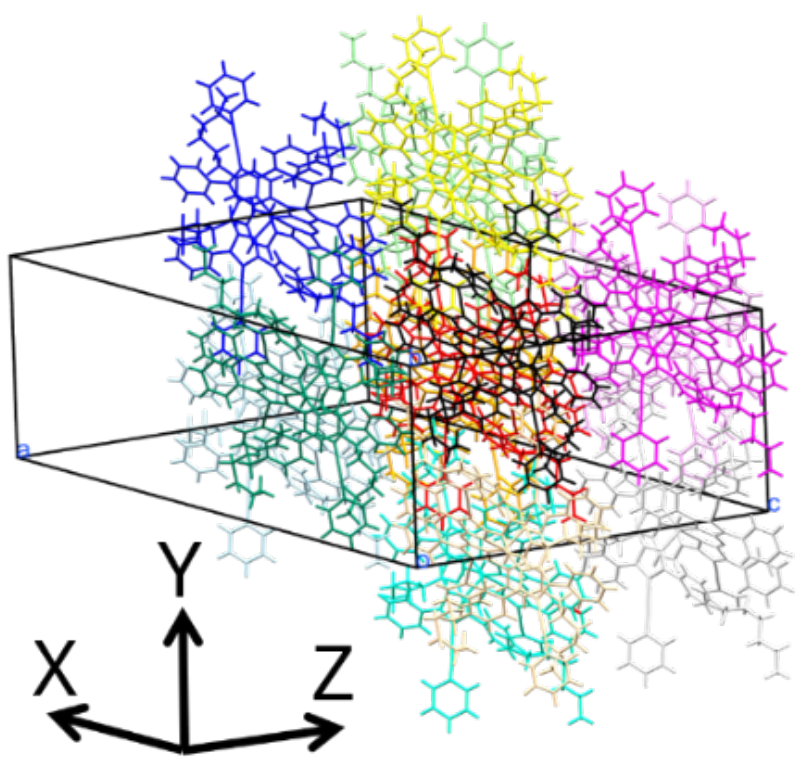

Pair	Direction from red	Interacting Groups	Vh^+ (meV)	Ve^- (meV)
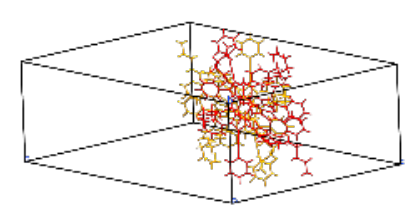	+X, -Y, +Z	2 distal-pyrrolic (CF) proximal-proximal (PD)	12.03	3.53
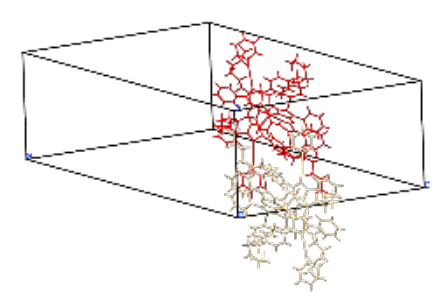	-X, -Y, -Z	2 distal-pyrrolic (CF), proximal-proximal (PD)	12.03	3.53
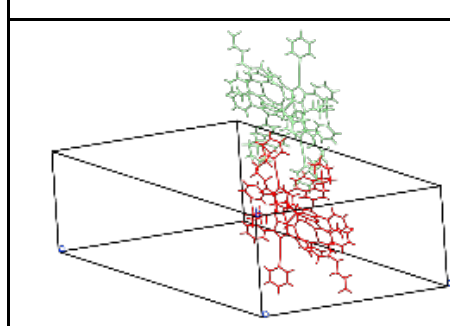	+X, +Y, +Z	2 proximal-pyrrolic (CF)	2.51	4.52
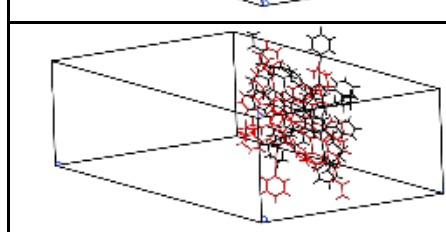	-X, +Y, -Z	2 proximal-pyrrolic (CF)	2.51	4.52
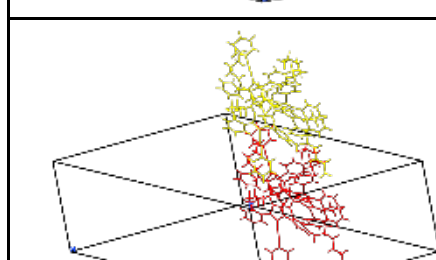	+Y	2 pyrrolic-pyrrolic (EE)	1.38	0.20
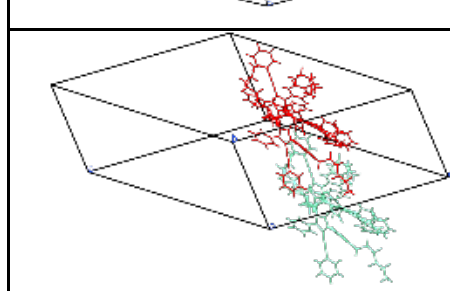	-Y	2 pyrrolic-pyrrolic (EE)	1.38	0.20
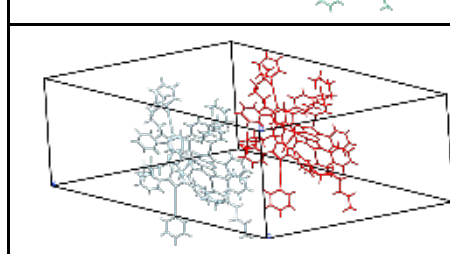	+X, -Y	distal-pyrrolic (EE), distal-proximal (EE)	0.10	0.57
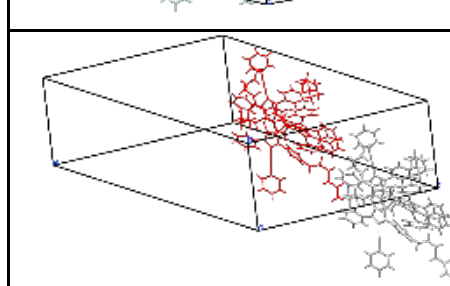	-X, -Y	distal-pyrrolic (EE), distal-proximal (EE)	0.10	0.57
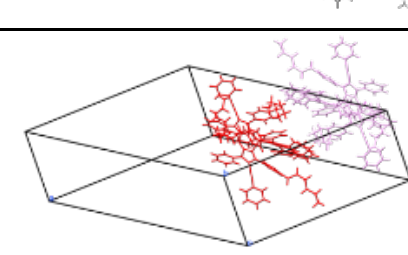	+Z	2 distal-proximal (PD)	0.56	0.22
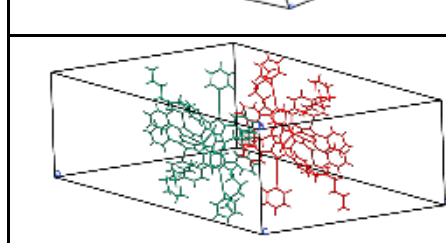	-Z	2 distal-proximal (PD)	0.56	0.22

	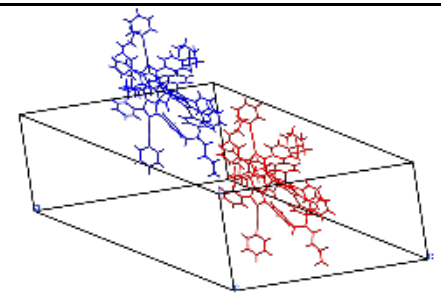	+X, +Y	distal-pyrrolic (EE), distal-distal (EE)	0.41	0.35
	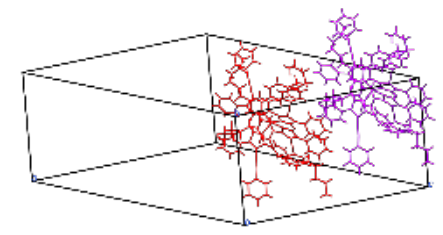	-X, +Y	distal-pyrrolic (EE), distal-distal (EE)	0.41	0.35

CF = cofacial, face-to-face
PD = parallel-displaced
T = T-shaped
EE = edge-to-edge

Table S4: Crystal packing of nearest pairs to the selected Zn(L1-*m*-2F)$_2$ molecule in red. All nearest pairs with overlap over 0.1 meV are listed. The direction from the red molecule, the main π-π stacking interaction(s), and the hole and electron overlap integrals (in meV) are included.

Zn(L1-*m*-2F)$_2$

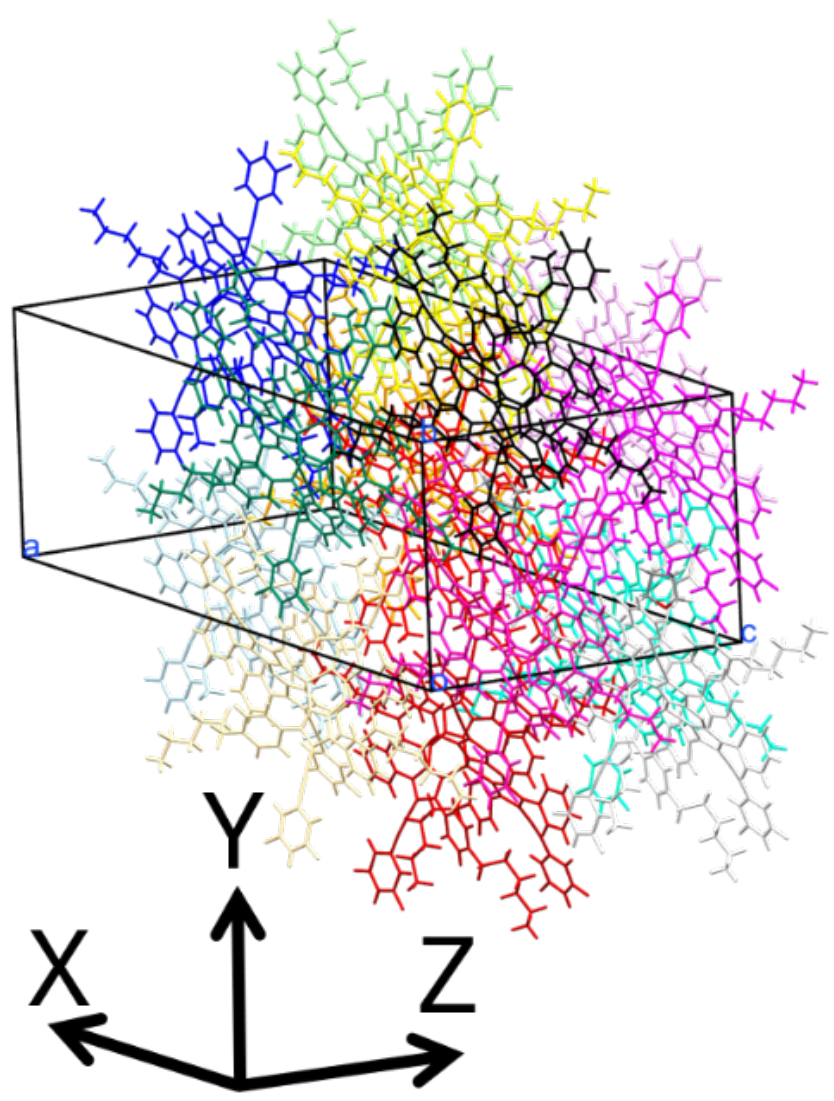

Pair	Direction from red	Interacting Groups	Vh$^+$ (meV)	Ve$^-$ (meV)
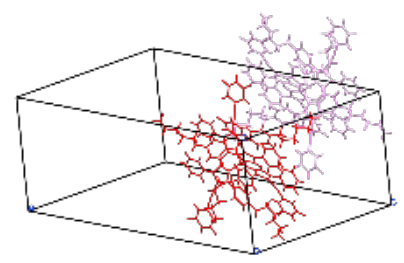	+Y, +Z	2 distal-pyrrolic (CF), proximal-proximal (PD)	25.5	10.6
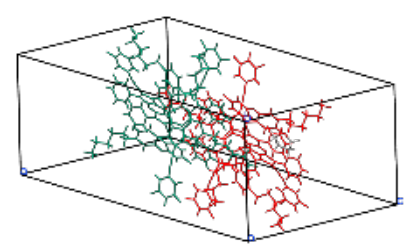	+Y, -Z	2 distal-pyrrolic (CF), proximal-proximal (PD)	25.5	10.6
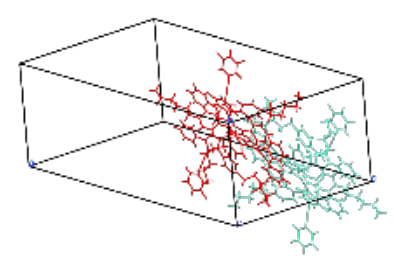	-Y, +Z	2 proximal-pyrrolic (CF)	3.66	9.47
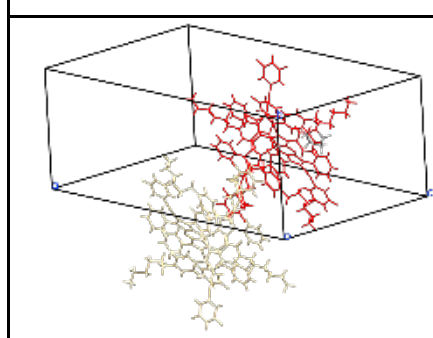	-Y, -Z	2 proximal-pyrrolic (CF)	3.66	9.47
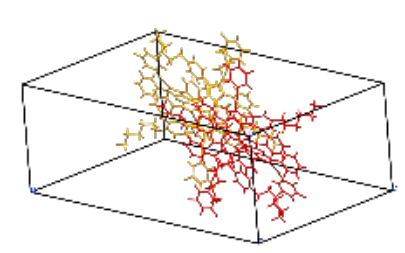	+X, +Z	2 distal-proximal (PD)	1.88	1.15
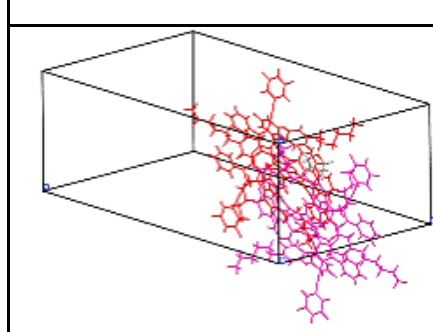	-X, -Z	2 distal-proximal (PD)	1.88	1.15
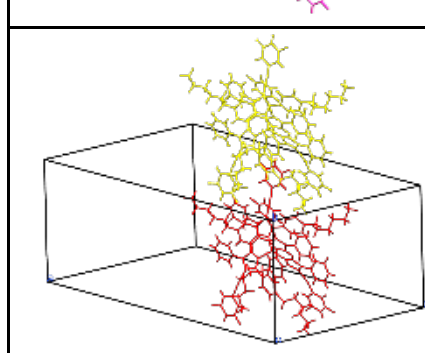	+Y	2 pyrrolic-pyrrolic (EE)	0.60	0.37
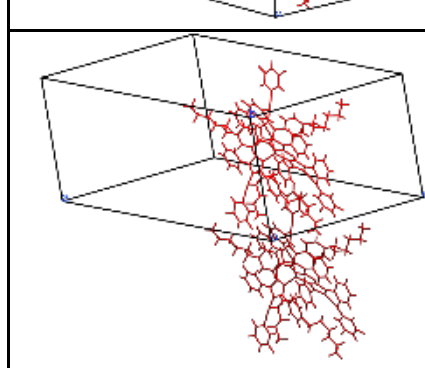	-Y	2 pyrrolic-pyrrolic (EE)	0.60	0.37
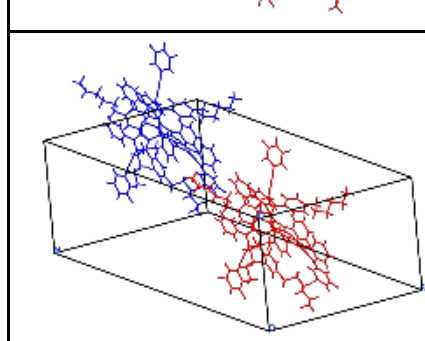	+X, +Y	distal-pyrrolic (EE), distal-distal (EE)	0.31	0.55

	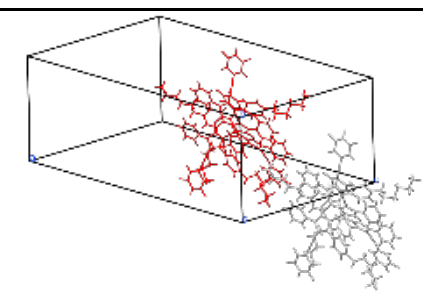	-X, -Y	distal-pyrrolic (EE), distal-distal (EE)	0.31	0.55
	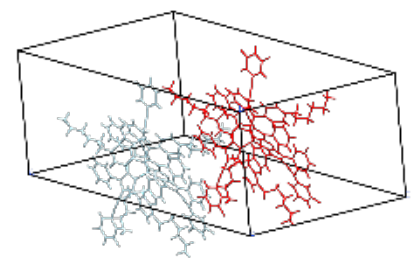	+X, -Y	distal-pyrrolic (EE), distal-distal (EE)	0.30	0.40
	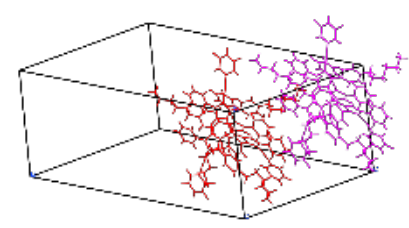	-X, +Y	distal-pyrrolic (EE), distal-distal (EE)	0.30	0.40
	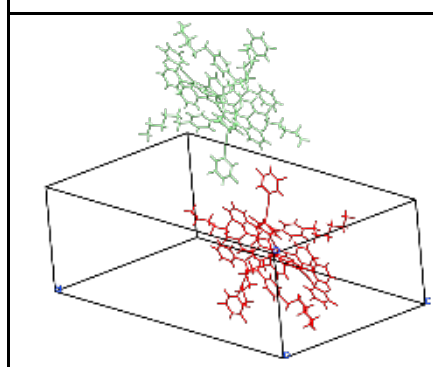	+X, +Y, +Z	pyrrolic-pyrrolic (EE)	0.34	0.38
	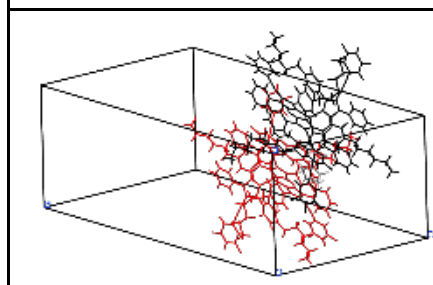	-X, +Y, -Z	pyrrolic-pyrrolic (EE)	0.34	0.38

CF = cofacial, face-to-face
PD = parallel-displaced
T = T-shaped
EE = edge-to-edge

Table S5: Crystal packing of nearest pairs to the selected Zn(L-*m*-2CF_3)$_2$ molecule in red. All nearest pairs with overlap over 0.1 meV are listed. The direction from the red molecule, the main π-π stacking interaction, and the hole and electron overlap integrals (in meV) are included.

Zn(L1-*m*-2CF_3)$_2$

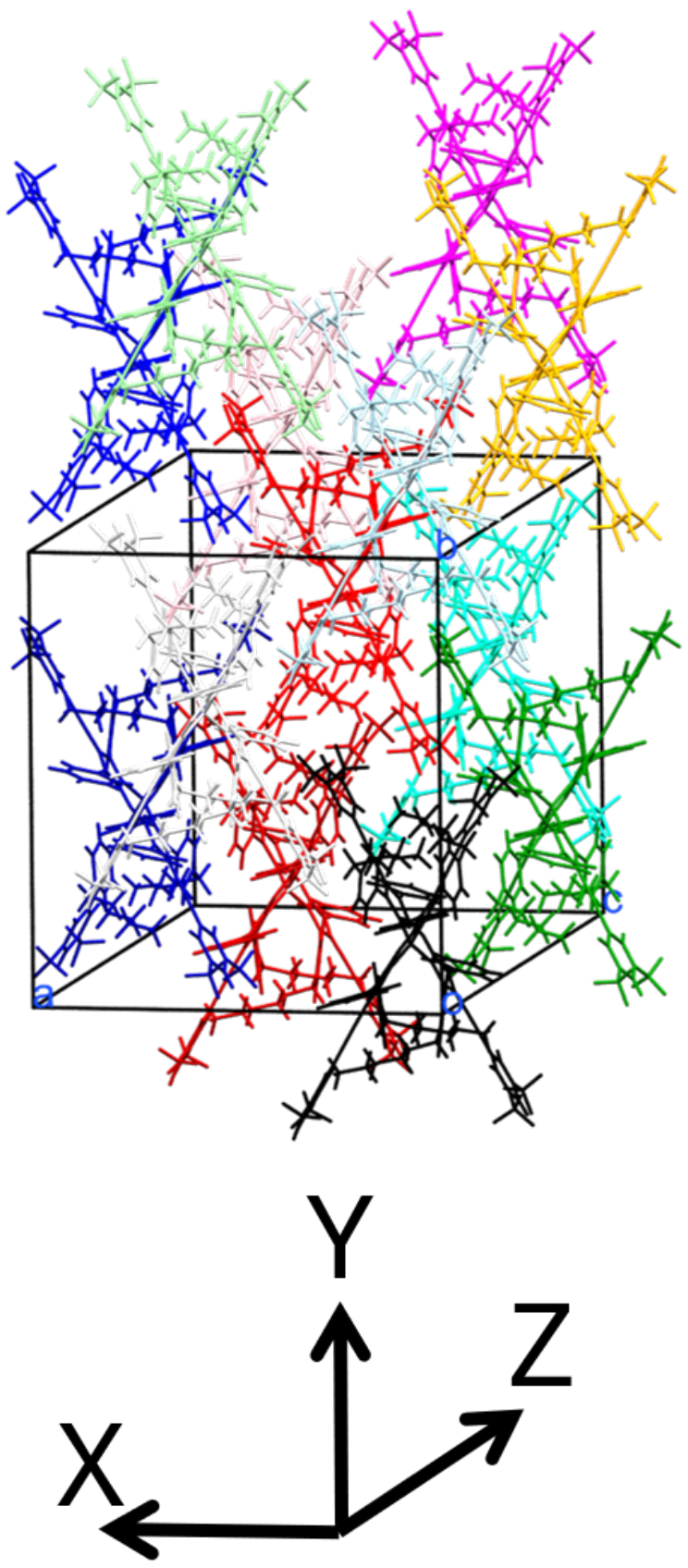

Pair	Direction from red	Interactions	Vh^+ (meV)	Ve^- (meV)
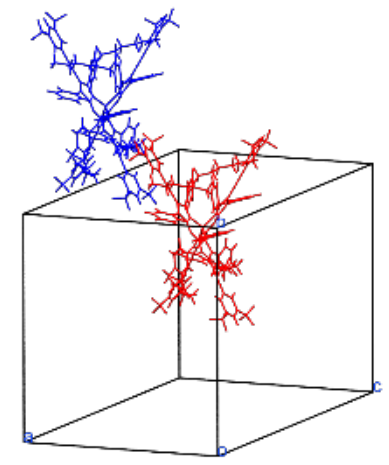	+X, +Y	pyrrolic-pyrrolic (CF)	23.2	7.49
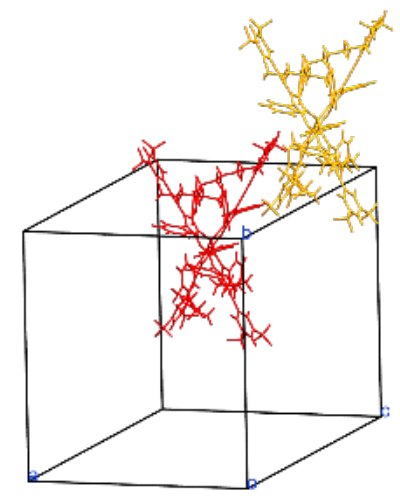	-X, +Y	pyrrolic-pyrrolic (CF)	23.2	7.49
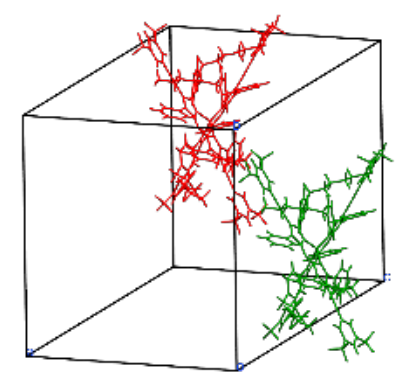	-X, -Y	pyrrolic-pyrrolic (CF)	23.2	7.49
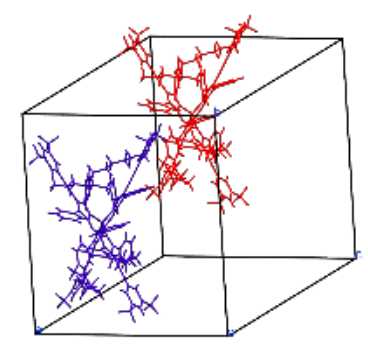	+X, -Y	pyrrolic-pyrrolic (CF)	23.2	7.49
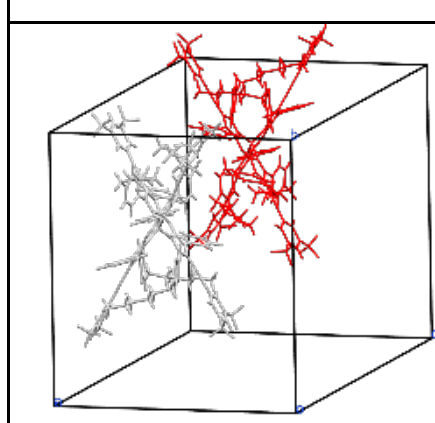	+X, -Y, -Z	2 distal-proximal (T)	0.74	0.59
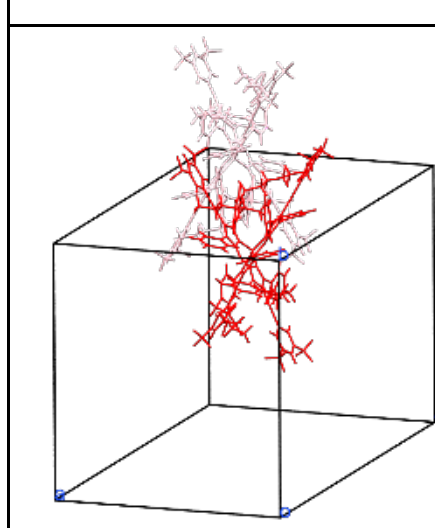	+X, +Y, +Z	2 distal-proximal (T)	0.74	0.59

	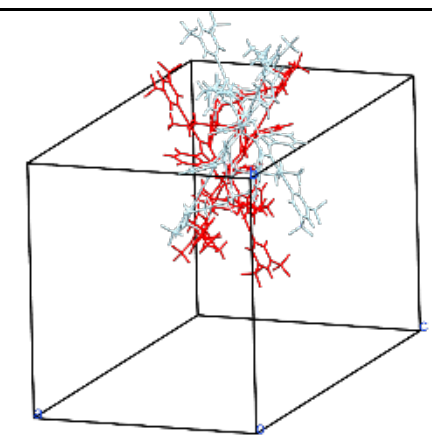	-X, +Y, -Z	2 distal-proximal (T)	0.74	0.59
	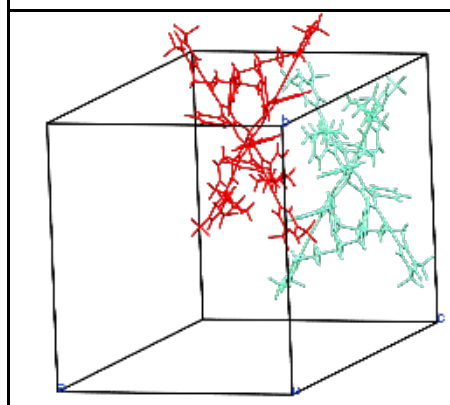	-X, -Y, +Z	2 distal-proximal (T)	0.74	0.59
	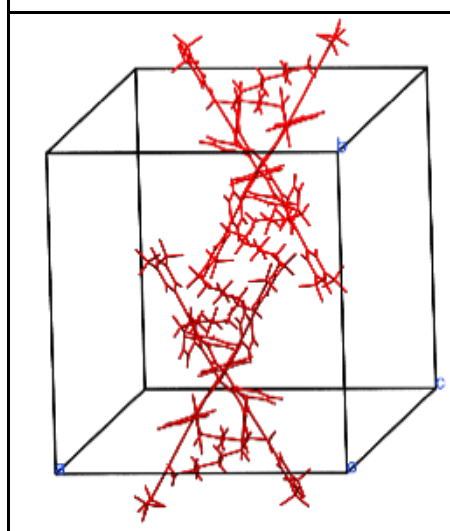	+X,-Y, +Z	pyrrolic-pyrrolic (PD)	0.21	0.20
	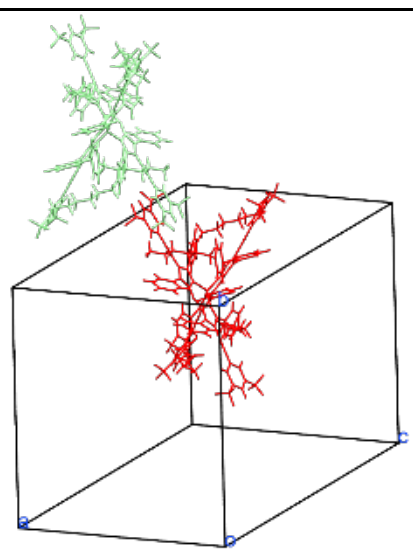	+X, +Y, -Z	pyrrolic-pyrrolic (PD)	0.21	0.20
	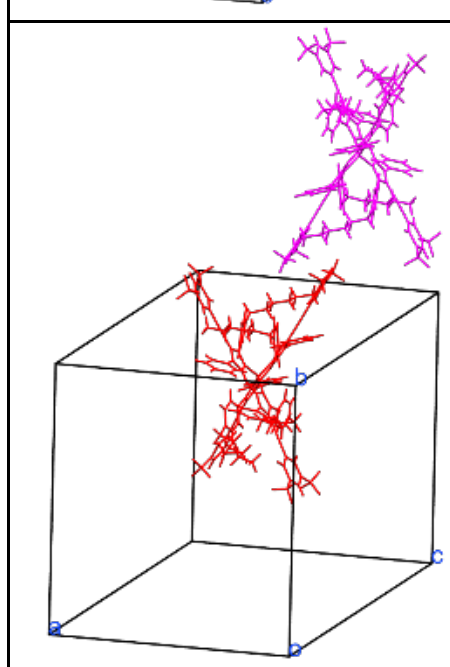	-X, +Y, +Z	pyrrolic-pyrrolic (PD)	0.21	0.20
	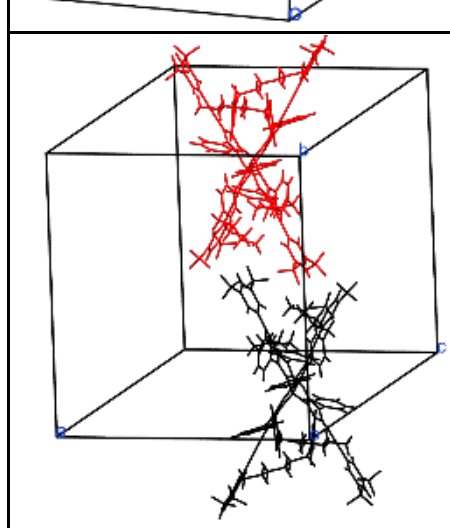	-X, -Y, -Z	pyrrolic-pyrrolic (PD)	0.21	0.20

CF = cofacial, face-to-face
PD = parallel-displaced
T = T-shaped
EE = edge-to-edge

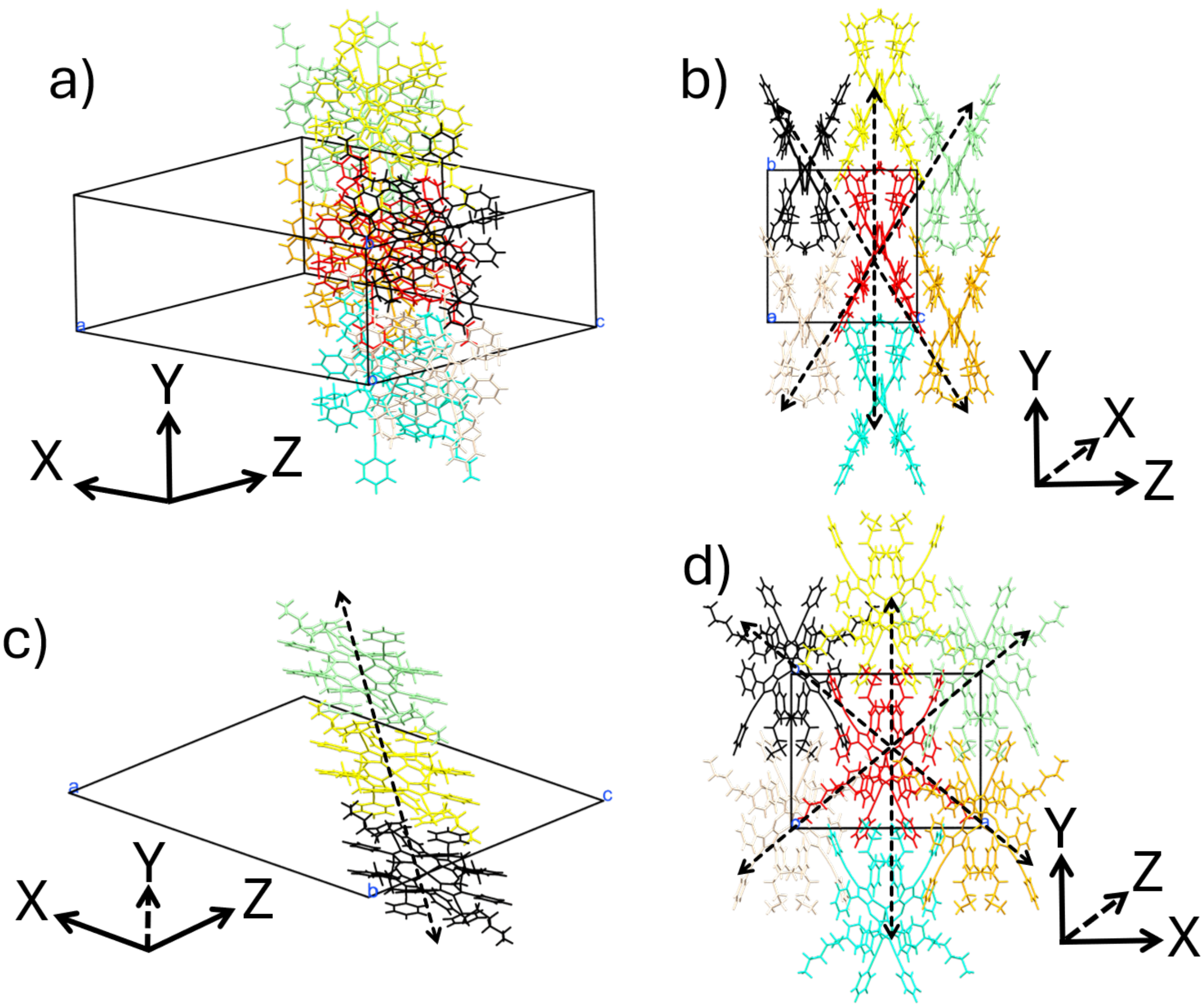

Figure S23: $Zn(L1)_2$ nearest pairs with strong overlap integrals (>1 meV), corresponding to Table S3. Dotted black arrows indicate the direction of charge transport as estimated by the overlap integrals. a) Isometric view, b) viewed along x-axis, c) viewed along y-axis, and d) viewed along z-axis. The collection of nearest pairs forms a plane, which parallel to the y-axis, indicating most of the overlap occurs in mainly two dimensions.

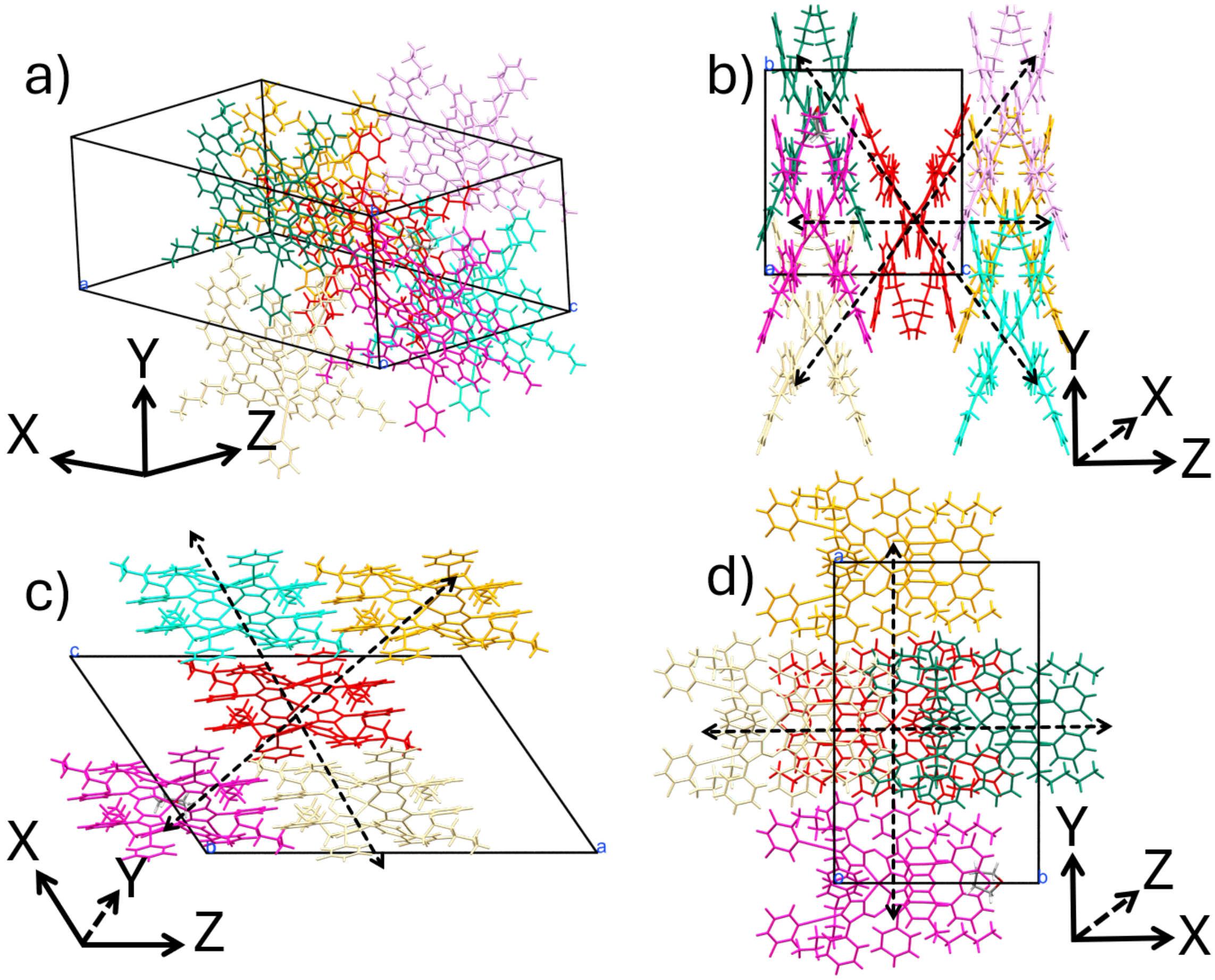

Figure S24: Zn(L1-*m*-2F)$_2$ nearest pairs with strong overlap integrals (>1 meV), corresponding to Table S4. Dotted black arrows indicate the direction of charge transport as estimated by the overlap integrals. a) Isometric view, b) viewed along x-axis, c) viewed along y-axis, and d) viewed along z-axis. The collection of nearest pairs indicates most of the overlap occurs in three dimensions.

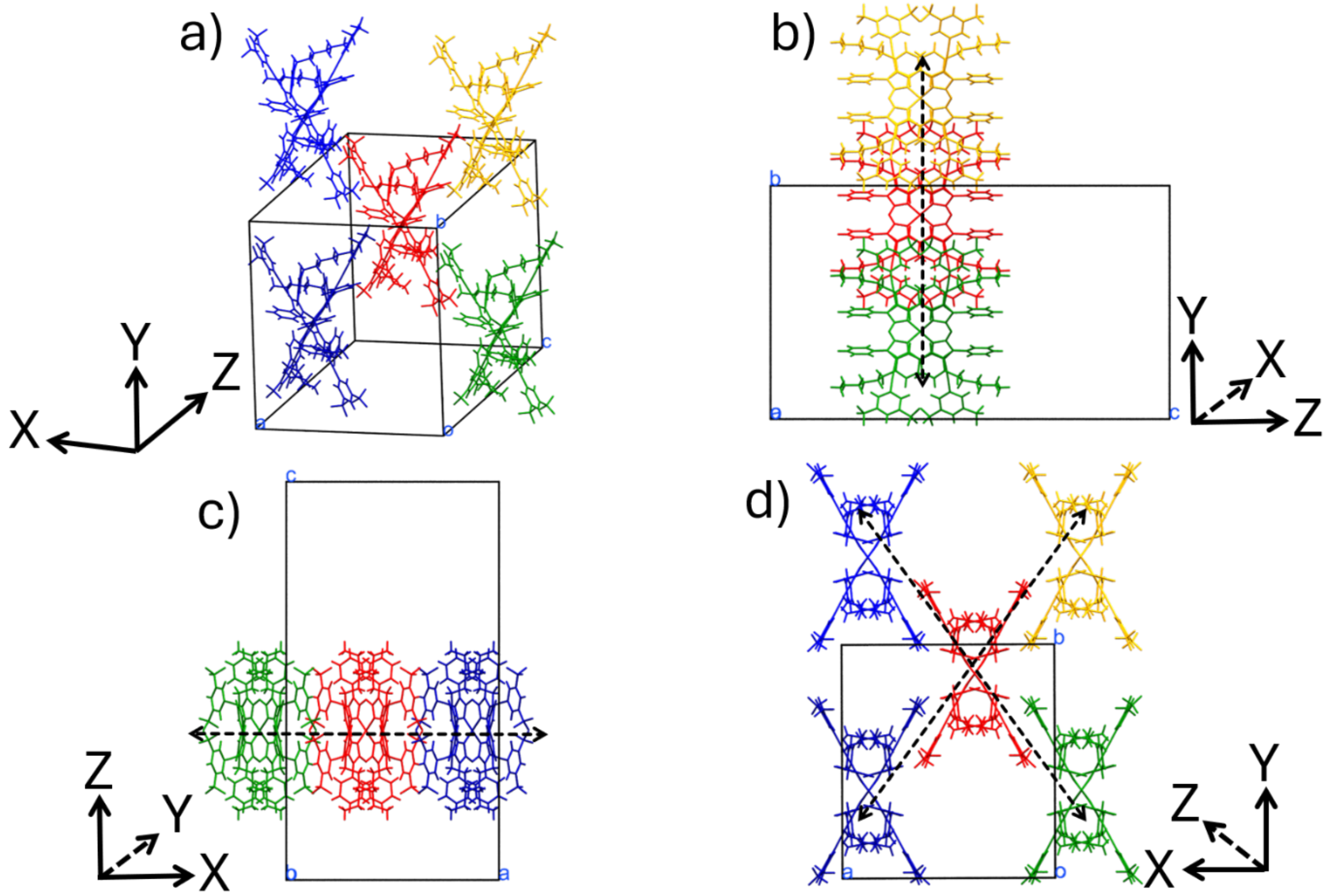

Figure S25: $Zn(L1\text{-}m\text{-}2CF_3)_2$ nearest pairs with strong overlap integrals (>1 meV), corresponding to Table S5. Dotted black arrows indicate the direction of charge transport as estimated by the overlap integrals. a) Isometric view, b) viewed along x-axis, c) viewed along y-axis, and d) viewed along z-axis. The collection of nearest pairs indicates most of the overlap occurs mainly in two dimensions.

Computational data

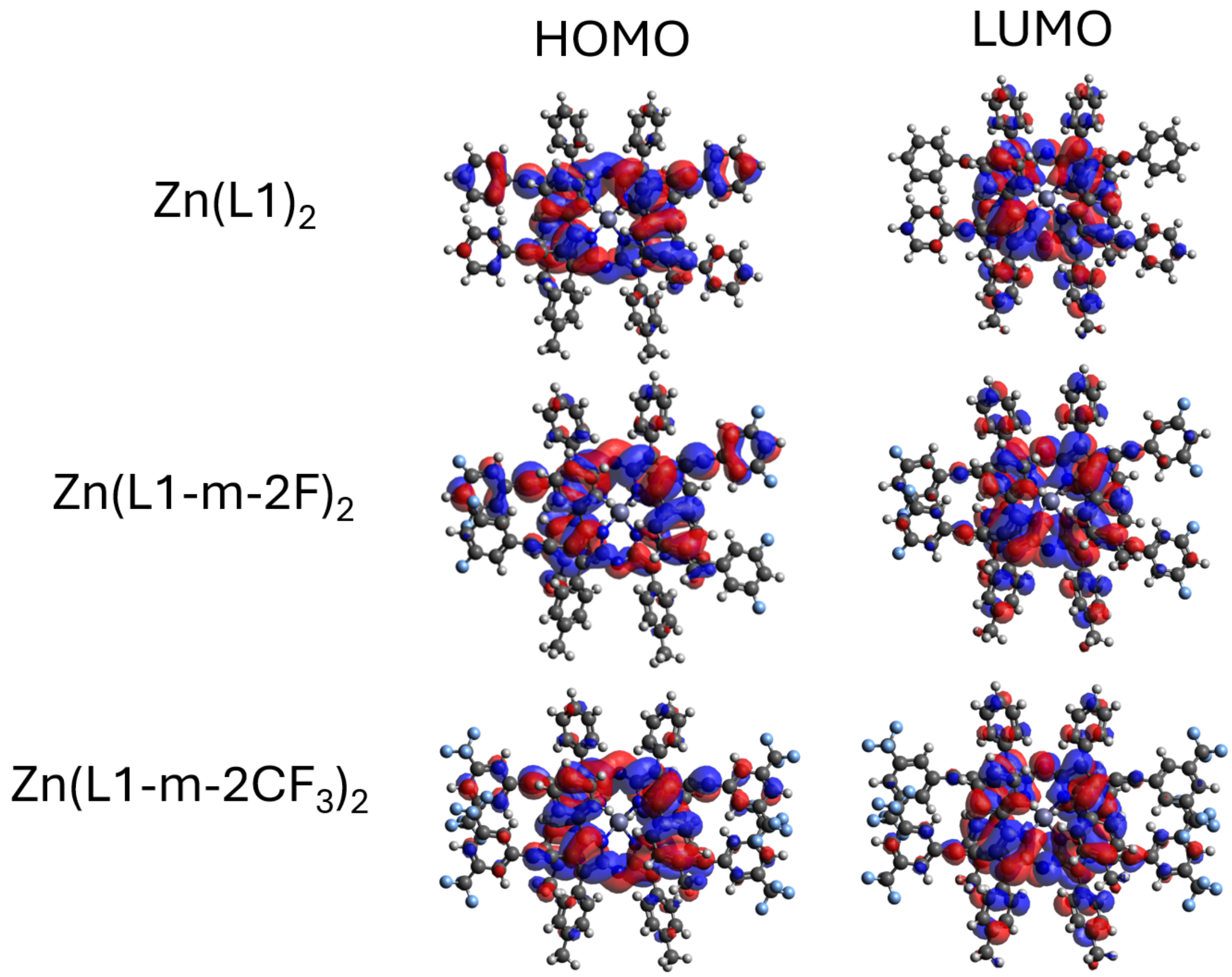

Figure S26: HOMO/LUMO contour plots of $Zn(L1)_2$, Zn(L1-*m*-2F)$_2$, and Zn(L1-*m*-2CF$_3$)$_2$.

Table S6: Coordinates used for the DFT calculations of Zn(L1)2.

C	1.95469224026417	6.29379413469033	-1.30116838575623
C	1.84774123285638	7.45246273936682	-2.05968911432126
C	1.11058313685882	7.47527414452651	-3.25424726061898
H	0.10522091422764	4.21600551377686	-3.24880376433934
C	0.48318403453818	6.29022316923327	-3.65678756868160
C	4.85799109424892	4.63700642118769	0.08984728280385
C	0.58404202259823	5.12397810023939	-2.90364403300950
C	1.32145925503196	5.10099486531960	-1.70530841053848
H	-0.08726232140279	6.27809100986145	-4.58279719182634

H	2.51627775090273	6.31083919192076	-0.37328802430802
H	2.33983764775793	8.36017890699343	-1.71709265159458
C	1.00359298785271	8.73555808355884	-4.07443404158111
C	2.93512673822999	-0.32034575496291	3.13635020731385
C	2.23044309989689	0.55409093610730	2.31725575467572
C	2.90376087938339	1.36983093399421	1.38838276918174
H	6.09079016385522	0.37067815325507	2.07179835782483
C	4.30733076938883	1.30591157108838	1.32881334144981
H	4.87655516078628	-1.08925988259403	3.67886423244374
C	5.00879204233729	0.42724032406114	2.14958151765758
C	4.32777743864115	-0.39384201484283	3.05048164123576
H	4.83980390436194	1.91740867828470	0.61126492654907
H	2.39654367960103	-0.94944914154736	3.83879508869716
H	1.14976849222706	0.61474912516804	2.40233417038482
N	0.90956857668556	1.85536836959249	0.06470155662591
C	2.14282624906500	2.20752046002591	0.47028088834462
Zn	0.11126186786593	0.02797152339705	-0.00413143433686
C	0.44417113971525	2.85546351169796	-0.78781240100004
C	2.52603418398134	3.46246593995656	-0.12869835991977
C	1.43653042065970	3.88881286132343	-0.90851597296612
N	-0.74302000084506	2.90500064516515	-1.37134828960059
C	3.76671605020365	4.09750579401350	0.01070654959963
H	9.63428072773275	6.94055396975021	0.39328862283062
H	-10.61402683672323	1.37138049909598	-2.12873832478899
H	5.64136077114591	6.83740751161866	-1.21022791122086
H	-9.58422090956538	-2.48870259817816	-0.52876153871496
C	6.41258001968959	6.41777430606502	-0.57139552276800
C	7.66626361179973	7.01551888023213	-0.48911976555094
C	8.65711060319150	6.47045442243458	0.33275992168816
H	6.92310010786885	3.81531365924219	1.57850139949341
C	8.38599725302230	5.31779580302376	1.07588686833868
C	6.12962792806353	5.25367741295161	0.17523022309930
C	7.13647429282401	4.71040009529567	1.00214004722325
C	-4.73210168354602	3.41494623148145	-3.05562126860209

C	-5.34113192820756	4.61975251582969	-3.39285653036734
C	-4.88804648045698	5.83687358125466	-2.86719307207166
H	-2.35833590721045	4.60225793326723	-0.94040282555281
C	-3.79745287631759	5.80152084456404	-1.98573900699324
C	-6.15202675887681	0.28478392304021	-1.81182748067643
C	-3.18533749327322	4.60312587169654	-1.63927103437711
C	-3.63738319193167	3.37814368044170	-2.17184894738381
H	-3.42907945547537	6.72927738638939	-1.55323149123813
H	-5.09348449068349	2.49209078085460	-3.49667992929350
H	-6.18032026603980	4.61778068289538	-4.08483343168151
C	-5.54978907625284	7.14182234205546	-3.22992682812899
C	-1.49507763892886	-3.69543086860050	-1.42969552747738
C	-1.46891686757771	-2.31436492031316	-1.58134829281006
C	-2.61178614211532	-1.53889224620260	-1.30458833522447
H	-4.71274681987332	-4.06319316311044	-0.39112244871906
C	-3.78376487381001	-2.19427010998238	-0.88831369735273
H	-2.67807218754698	-5.41182369670763	-0.87265600491673
C	-3.80543559231584	-3.57766505696930	-0.73861052464886
C	-2.66322990705237	-4.33394942022133	-1.00396344954006
H	-4.66327161583807	-1.61168518035715	-0.64900173922621
H	-0.60117699609017	-4.27521928665307	-1.64029167222509
H	-0.56348738463066	-1.82905766393306	-1.93122774454706
N	-1.38502797796640	0.57037362353833	-1.22196124294948
C	-2.54239368829697	-0.08730236647017	-1.41671885318932
C	-7.52953831872181	-0.01432214342745	-1.67073598972703
C	-1.62584387595888	1.92291416021895	-1.45679764047502
C	-3.59443134410065	0.84106632171434	-1.75566358340922
C	-3.00186199695541	2.11409331469514	-1.83181183836837
C	-7.93547463809642	-1.25405339535745	-1.13005347988508
C	-4.95812150074894	0.53301520828187	-1.85198923986090
H	-11.30958853033542	-0.81433877508381	-1.16964976626327
H	7.87234715645345	7.91073347789846	-1.06902912178029
H	-8.20878235260553	1.88495916761066	-2.44353147501180
H	9.15273333055358	4.89025450858621	1.71578817704682

C	-8.51557569744053	0.92883339531629	-2.03053918805678
C	-9.86407141912219	0.63650135751664	-1.84987259520978
C	-10.25584446340819	-0.59246308286691	-1.31091731856485
H	-7.18220429639656	-1.98220669125795	-0.84610328000597
C	-9.28640702457875	-1.53380830351523	-0.95257182803223
C	-0.63721570751409	-5.45864665247118	1.96129779730059
C	-0.59075439647648	-6.78905230910555	2.37056058498084
C	-1.28542098470564	-7.20293636171028	3.50943530333302
H	-2.64145573839978	-4.21939691842295	4.41658497926393
C	-2.02942873263285	-6.27145624805452	4.23895553772688
C	-4.98194156355775	-2.85435121358139	3.03126973472159
C	-2.07705199313126	-4.94039014073044	3.83451622934698
C	-1.37865143859785	-4.50849014634068	2.69026273056000
H	-2.56876220517848	-6.58193160904357	5.12971743198963
H	-0.11111743922754	-5.14575912963139	1.06795495144414
H	-0.01530585115069	-7.50716999401640	1.79270863096395
H	-1.24942729852312	-8.24182039415897	3.82517444879351
C	-4.92684602911950	1.48937413313147	1.54536706744992
C	-4.22794710123852	0.29187499524842	1.63844527658130
C	-2.82901619263804	0.28376068080735	1.77202422203587
H	-2.32812513126170	3.65723279607177	1.75188684923435
C	-2.16173715647400	1.52407841471952	1.81177092988085
C	-5.02920468458151	4.01558663916222	1.47212208409034
C	-2.86845577500786	2.71442158270662	1.71733832023479
C	-4.26673489144732	2.72234490918414	1.58282296089599
H	-1.08410414954332	1.55214152666154	1.94060592870606
H	-6.00679930857805	1.46602481891041	1.42345358287548
H	-4.76412380675571	-0.64552164482620	1.57833800174980
N	-0.81041252306259	-1.05912440645355	1.40638175177265
C	-2.08064468298059	-0.96218781430272	1.84213214929402
C	9.74909981766589	-3.51804163517305	-1.47785113840457
C	-0.38255143024854	-2.36527092914133	1.64531236882795
C	-2.52494056466482	-2.23353895814248	2.36890579775438
C	-1.43423250889398	-3.11146535408938	2.27925411840427

N	0.80281615551092	-2.87976527715628	1.36092068834841
C	-3.82823863384396	-2.55039019255626	2.77578962904571
H	-10.09288885402193	-4.11896406472073	3.73949877816215
H	-9.47190596142280	-1.90967673252402	2.77769800149502
H	-5.92511995104918	-5.14181458645604	4.06613568161204
H	-8.31247860184809	-5.73028434241672	4.38401043964510
C	-6.70444613708828	-4.43773891827465	3.79082613276784
C	-8.04594287818949	-4.76385961840868	3.96556353472196
C	-9.04740069507084	-3.85810508690017	3.60360112775261
H	-7.08950736741733	-1.31587104478544	2.46380915111851
C	-8.69829730850131	-2.61627581576455	3.06481124163876
C	-6.34221405142496	-3.18722702029416	3.24670405882841
C	-7.36088919297540	-2.27777388575914	2.88722383888948
C	3.19038710885564	-4.32134982558983	2.51656600377410
C	3.67958817898559	-5.43343241564170	3.19743874698701
C	4.56468460637820	-6.31282513021527	2.56949328757436
H	4.76914929389402	-4.78662777258373	-0.46276578851173
C	4.95915089632356	-6.06937999162654	1.25101704131175
C	6.22791060095948	-2.32458342255546	-1.23059260338561
C	4.47266058884657	-4.95908041613772	0.56638661175842
C	3.57528589892327	-4.06770444266564	1.18560083278622
H	5.64336905680768	-6.75044069314057	0.75230889037107
H	2.51593956304289	-3.63336514151025	3.01207162263030
H	3.37333750239713	-5.61034207918351	4.22485561148292
H	4.94575411806173	-7.17913000645281	3.10293476779370
C	2.13497666782850	1.60780429661742	-3.42948674602939
C	1.94541242117456	0.50905688883777	-2.60030058459839
C	3.00938082522445	-0.01304335312255	-1.84383317554837
H	5.43644945687571	2.14752763024121	-2.87310244354420
C	4.27641643250409	0.58389157759267	-1.98163032724604
C	3.57716856461884	3.46424407849992	-4.37551386427997
C	4.45442235712942	1.68506064207252	-2.80830848630360
C	3.38705429977506	2.22879909089916	-3.53657164857454
H	5.11118646156062	0.20533660302105	-1.40496509704264

H	1.29537363707367	1.99740344394112	-3.99881467652007
H	0.96491334931458	0.04489309854793	-2.54571138739060
N	1.61206992386530	-1.25971677671189	-0.26467091912035
C	2.78924457685635	-1.09307247054721	-0.89465076186925
C	7.58197449655829	-2.42884098652448	-1.63165104934219
C	1.73985134782822	-2.35872295100738	0.58555448487822
C	3.73145335382127	-2.10062615796400	-0.46459058252633
C	3.06008267929849	-2.91165154720340	0.46321898294954
C	8.41861178992786	-3.42397153264425	-1.08191170675536
C	5.06188912032271	-2.22237827257229	-0.88650282383238
H	11.30948264764111	-2.70730936390837	-2.72900634193007
H	10.38315258142696	-4.28796427462640	-1.04731256579028
H	7.47689221217076	-0.76983208815361	-3.01168534423295
H	9.84937942290394	-0.94852179052284	-3.70836146381974
C	8.11707074655174	-1.53633139897578	-2.58561253878574
C	9.44907041154745	-1.64114308934948	-2.97339685024698
C	10.27028023834806	-2.62973186683087	-2.42310806133008
H	8.01124266924171	-4.11184187449089	-0.34722037080363
H	2.74381086831919	3.60505493603379	-5.06878991602338
H	4.51092926841181	3.41586168126638	-4.94551020677643
H	3.63082571448132	4.35233506379775	-3.73322718860563
H	-6.10770507075368	3.83781772937853	1.49864429400103
H	-4.78874319477392	4.52497300084649	0.53152936066293
H	-4.76569248282250	4.69913918940813	2.28710455892905
H	0.38651338131505	8.57488840391766	-4.96269022919580
H	1.99346030307792	9.07759012909081	-4.39943374501730
H	0.55887545746026	9.54817730963431	-3.48778990903794
H	-6.37342866515912	6.98364236009911	-3.93152965578290
H	-4.83144406248179	7.83073423774880	-3.68996217185591
H	-5.94799482571987	7.64018147626739	-2.33803065529122

Table S7: Coordinates used for the DFT calculations of Zn(L1-*m*-2F)$_2$.

C	3.21859561215404	5.91445788069444	0.36473175601724
C	3.37668574478198	7.21336197903017	-0.10169166585031
C	2.76964049995022	7.63733839133161	-1.29423422972744
H	1.25185538649872	4.69696332142174	-2.12301598221787
C	1.99934404191862	6.70691782458486	-2.00306821219244
C	5.68599154912755	3.48847086266384	1.37069589731859
C	1.83382852546225	5.40375112311285	-1.54395940268681
C	2.43865452657620	4.98011161757521	-0.34547850481553
H	1.53025050188973	7.00572201120238	-2.93768536342367
H	3.68511403461076	5.61965316411382	1.29861788670003
H	3.97882689829595	7.91536889854935	0.47085811969733
C	2.93159397849752	9.05319997580501	-1.78507298725047
C	2.70836662320449	-1.67175877784183	3.01034770710424
C	2.22913998014944	-0.50529025269321	2.42570110953054
C	3.10460716111596	0.39652488872016	1.79152876256657
H	6.02236989294078	-1.27463621518516	2.35692016371857
C	4.48182563499176	0.11073005758590	1.79750025974838
H	4.44866518023015	-2.87682662387373	3.42786319863176
C	4.95837132333928	-1.05779101008909	2.38463601396379
C	4.07545812614046	-1.95833242119038	2.98434940660726
H	5.16980443395206	0.78520531039814	1.30368821693392
H	2.01378772076993	-2.35922595127049	3.48373031815614
H	1.16665632232351	-0.28740966150758	2.46956751321052
N	1.33564676793382	1.54916435739596	0.56190866444814

C	2.57391532484005	1.55759115799083	1.08769593280680
Zn	0.21776501622172	-0.01346077849431	0.05256526985619
C	1.11508601413271	2.79147063898759	-0.03066138796521
C	3.21171443889899	2.82540051879878	0.83006691444476
C	2.27699968681243	3.61908631855495	0.14056167103775
N	0.00078252637964	3.20347971508723	-0.61753001507289
C	4.52870687223457	3.17497810186406	1.14876283644893
H	10.76117169999624	4.84368430818181	2.23229699342072
F	-9.48558561957491	-1.30468355691417	-2.12753018154709
H	6.87507683506746	5.77992852789537	0.61449448370566
F	-10.15257480688788	3.19034537108082	-0.87870953527064
C	7.52007052265300	5.08989077235366	1.14583307778966
C	8.84556309393209	5.40635874146521	1.38680233722459
C	9.72766922954979	4.56803944436701	2.05924337077233
H	7.54024936239302	2.00859862578323	2.65575277051241
C	9.20690450410057	3.35513854449552	2.49599075251712
C	7.03275047631519	3.84811541438244	1.60637229653366
C	7.89402176533725	2.96680973982018	2.29368921878488
C	-3.77132364760421	4.78706385706230	-2.20715453744379
C	-4.16707195731718	6.11858157010555	-2.23832092574654
C	-3.54921686198448	7.08025794834259	-1.42475475810226
H	-1.32546826788052	5.01865459535274	0.13621650045523
C	-2.51311434871018	6.65518819352547	-0.58287789132546
C	-5.70392483835943	1.64067879609055	-1.84875598839334

C	-2.11280117503660	5.32452582073913	-0.54071416303090
C	-2.73267071694411	4.35756402035414	-1.35638125140733
H	-2.01895896326309	7.37887577932421	0.06147233018506
H	-4.25124576822857	4.07172224313056	-2.86690712221783
H	-4.96328261508445	6.42262532784131	-2.91406671351904
C	-4.00529670353770	8.51651279209815	-1.43534248115568
C	-4.05027270093290	-2.61401273214431	-1.82411011875726
C	-3.80815179145254	-1.26832950437946	-1.56626199837932
C	-2.53715009611584	-0.71558375682763	-1.79972418286183
H	-0.96901077498283	-3.52193859344184	-2.94325766874274
C	-1.51890251454675	-1.54991739707146	-2.30003674660355
H	-3.22529985938145	-4.48210973690126	-2.51558116133742
C	-1.76649760569383	-2.89307662912909	-2.55832181238594
C	-3.03450484784749	-3.43087819245334	-2.32234862390587
H	-0.53930828956418	-1.13126761359118	-2.50483025585419
H	-5.03140085890354	-3.02969723657614	-1.61367586920906
H	-4.59109183919609	-0.65226287030414	-1.14461555489647
N	-1.01691016237405	1.06165301044616	-1.10457370067776
C	-2.23864451513496	0.68276808411551	-1.51355893715649
C	-7.09864385504143	1.42067024940436	-1.76754208120613
C	-1.02429741704897	2.45026151853612	-0.98209167243965
C	-3.10541354054575	1.83067178432171	-1.62896838263024
C	-2.32204356004277	2.96341191825364	-1.34051679053332
C	-7.96540017920008	2.46399791288882	-1.38050603457539

C	-4.49059620930673	1.76391258520568	-1.82065718693873
H	-10.93224646997442	0.74892536456593	-1.39858014165040
F	9.31767851709837	6.60345152772928	0.94271962881660
H	-6.97379553078637	-0.68276759712807	-2.32077793124485
F	10.03959546817291	2.50601414120421	3.15821228005403
C	-7.62101159790553	0.13524932921499	-2.02798374307035
C	-8.98267345349615	-0.06264328108627	-1.88748030158740
C	-9.87088278874049	0.93655203347943	-1.50744741976946
H	-7.58508566098646	3.45628200626758	-1.16833052478959
C	-9.31627672586482	2.18701305267252	-1.26232566902007
C	-3.76120181726845	-4.85714805261346	2.24265936124042
C	-4.14956971509988	-6.19333471346826	2.26106644037644
C	-3.54519170006139	-7.11413119581353	1.40056595928626
H	-1.40134774218403	-5.01423658072278	-0.20028448758999
C	-2.54904279533856	-6.68349532347768	0.52124414448564
C	-5.77179565012841	-1.76665124855150	1.82374486129029
C	-2.15960814955473	-5.34707089249508	0.49672478574804
C	-2.75701959865696	-4.40866993264710	1.36167101607596
H	-2.07833648647282	-7.39036461703828	-0.15659031925837
H	-4.21995683786995	-4.15372864477609	2.92977469516114
H	-4.92041990287874	-6.51925859104883	2.95392873545616
H	-3.84974995946027	-8.15683379762491	1.41460266395809
C	-1.98550311294457	2.88547867238423	2.50423671968693
C	-1.67877666116631	1.55330376157869	2.25624467409456

C	-2.65812028427744	0.66665319774582	1.77167436984222
H	-5.24330364890143	2.87658285937034	1.56406290515734
C	-3.94856519115083	1.17300959518709	1.53332635306099
C	-3.61842230204040	4.82168407034362	2.58309579457168
C	-4.24419038841183	2.50656640527418	1.78117655585532
C	-3.27519343400472	3.38727203512127	2.28065297398829
H	-4.70876512016368	0.52751320613945	1.11439693190941
H	-1.21142185233483	3.54960265093130	2.88061898453952
H	-0.67844516459297	1.18735982251589	2.46208872902666
N	-1.07921163469706	-1.09098589296128	1.13546665892924
C	-2.31670979693862	-0.72483104474032	1.51195219937459
C	9.06542773365396	-3.42654917901050	-2.76857873573888
C	-1.06447216697064	-2.48095118500360	1.02156472307954
C	-3.16660735171730	-1.88802713606628	1.62224030540831
C	-2.36093350237947	-3.00786768926105	1.35525015978251
N	-0.03050357413861	-3.23219481419813	0.67546990766393
C	-4.55535652233629	-1.85395971927242	1.79774828915948
H	-11.02735214582069	-1.01920396593566	1.43048785446317
F	-10.18987950112898	-3.44466327642549	0.92619991903348
H	-7.09659488082414	0.52704293243005	2.28323159792730
F	-9.62647479437097	1.07968584803029	2.11920934463870
C	-7.72558910898674	-0.31096934004165	2.00782334040272
C	-9.09374037529240	-0.15090945953237	1.88420794335019
C	-9.95990492429916	-1.17730629113516	1.52634155601095

H	-7.61258553781239	-3.63959983698352	1.18484876005341
C	-9.37553159537751	-2.41529628947596	1.28712567599289
C	-7.17280831785531	-1.58504704588969	1.75423722685899
C	-8.01631234305527	-2.65520813195326	1.39039251081629
C	3.05497258808001	-5.99905724863389	-0.46856408014675
C	3.15235663227603	-7.33355730992311	-0.08521334433943
C	2.54977001959965	-7.77270031864090	1.09693507050066
H	1.21749044014141	-4.82227159426803	2.14174388908843
C	1.84939478531381	-6.86361288250132	1.89311908754654
C	5.62351454368213	-3.59356526075530	-1.42839917868187
C	1.74863026706939	-5.52751940482794	1.51367572045097
C	2.34692996023613	-5.07307882504907	0.32202505737718
H	1.38453427472252	-7.19481300316733	2.81761923666863
H	3.50794820103350	-5.66990017683749	-1.39768627866421
H	3.69424386935608	-8.03481672432091	-0.71378864174165
H	2.62790095437733	-8.81422940639328	1.39565527200674
C	5.09196854549880	1.05059612856993	-1.93159610696371
C	4.56674976950805	-0.14810102086775	-1.46563767900038
C	3.18835265688451	-0.41160025823046	-1.56262636725503
H	2.25866860632621	2.45881569488301	-3.15164595888901
C	2.37496168464455	0.54176052984678	-2.20126368920352
C	4.83413340082484	3.35790109394847	-2.93471790873489
C	2.91031124996745	1.73359019806977	-2.67188955156371
C	4.27368010570230	2.02391355024049	-2.52155424928175

H	1.31513226520603	0.34653460049197	-2.33475646020162
H	6.15426081418666	1.24833369438533	-1.81060914989745
H	5.21531883852978	-0.86362808721990	-0.97530542390262
N	1.35987596311204	-1.56644582617949	-0.42404127466472
C	2.60110336767018	-1.58796166216353	-0.93963092796907
C	6.94267291393539	-3.95951646783304	-1.78263959519624
C	1.09850339256272	-2.82593593282225	0.11565215200434
C	3.19600776881519	-2.88927538081811	-0.74231327716795
C	2.23661851872131	-3.67973611723590	-0.08807900797313
C	7.77963208235051	-3.03342226232011	-2.44092604689381
C	4.48932063186522	-3.26670310445499	-1.12210176257679
H	10.59350537260691	-4.96470501882937	-2.75432222151921
F	9.87441053622392	-2.53461201383404	-3.40332745839949
H	6.80011000828098	-5.97451183963940	-0.96998648619010
F	9.19293311855635	-6.81066500393686	-1.54102771698690
C	7.42634447135022	-5.24903859097914	-1.47588491669131
C	8.72421855351534	-5.56723334521365	-1.83605322609337
C	9.58163820341208	-4.68599807789675	-2.48507618671404
H	7.42768985235033	-2.03809500982272	-2.68569667044657
H	-4.25776184454445	5.24505034917728	1.80243297119098
H	-2.71702239780436	5.43424819717925	2.66733575573423
H	-4.16588514532081	4.89278095236752	3.53191325155578
H	4.20562423145674	3.83684604177271	-3.69055104811171
H	5.85169987631967	3.25855038695195	-3.32517578180426

H	4.87778865790927	4.02884006252447	-2.06690329450154
H	-4.36608147354869	8.80929805530997	-2.42622164541738
H	-3.19536256171363	9.19023257429475	-1.14012176903155
H	-4.83239738980486	8.66396766999905	-0.72843330032621
H	2.61009003881259	9.14835298637646	-2.82598167632092
H	3.97355780951861	9.38169266287608	-1.70746862271625
H	2.32838614002330	9.74339789998913	-1.18148741624738

Table S8: Coordinates used for the DFT calculations of Zn(L1-*m*-2CF_3)$_2$.

C	3.64216115140880	5.54640461444952	0.09215236928709
C	3.95455987625384	6.79366044685143	-0.43149655892638
C	3.29168101056250	7.29436812748781	-1.56372587216887
H	1.23518715015034	4.63166555615062	-2.12248312180663
C	2.30230562803574	6.49453528127171	-2.14804352518178
C	5.78724865104567	2.68102874751404	0.99987862131661
C	1.98424311199463	5.24156710749835	-1.63323732779746
C	2.64658406512226	4.73985432329875	-0.49685381519541
H	1.78149335451338	6.85359710416607	-3.03270601198192
H	4.15640286816432	5.19565492097847	0.98079213176284
H	4.72225908139316	7.39595766174736	0.04916530631081
C	3.63989071621651	8.64752489854362	-2.12851636346602
C	2.15532110755128	-1.60418844124947	3.34793037877942
C	1.82202802915110	-0.45757394971774	2.63707968062364
C	2.79476251853048	0.23325481324798	1.88827690931131
H	5.46334965839719	-1.77367322328813	2.57695492851642
C	4.11340665515299	-0.25386776973468	1.89200882522842
H	3.72395347773370	-2.98717117425831	3.87950241409672
C	4.44379899797742	-1.40080402228917	2.60743610849706
C	3.46803718030821	-2.08379260305718	3.33401276203457
H	4.86788459097897	0.24684037800533	1.30128095290707
H	1.38895611274334	-2.12663726846928	3.91283371232163

H	0.80521443478288	-0.08082020623061	2.67280560991847
N	1.15925401393816	1.51283128730710	0.60711325250802
C	2.40411223323768	1.39703329262161	1.10097949217628
Zn	-0.02408665186261	0.01362100458297	0.03016529477208
C	1.07130068825619	2.74408093264405	-0.04116185243510
C	3.18844346678055	2.55449568918992	0.74328356789265
C	2.33079658473460	3.43315012500904	0.05478991038451
N	-0.00815717016195	3.24149001711411	-0.62585199970040
C	4.56965876767512	2.67495812021713	0.92907792720682
H	11.06390724344548	2.05855952872021	0.89077640387141
C	-9.82662712095997	-0.69332610701744	-2.25712371223073
H	7.56211376820734	4.33071661119108	-0.16398601918164
C	-10.26550555983025	4.18248748791055	-1.17144128943112
C	8.01447409196010	3.47967514411134	0.33207178676438
C	9.39234217205096	3.29494028369340	0.30433613017325
C	9.98988075314111	2.19701965109496	0.92654349244609
H	7.17471613254825	0.72264116516165	2.15293667823443
C	9.17522094682501	1.27829260037178	1.58742806245273
C	7.19427514581872	2.54964337680737	1.00065882712616
C	7.79546535756951	1.44484871022145	1.63540968026551
C	-3.62788961101700	5.12211071124885	-2.19550087026508
C	-3.92708182647230	6.48046950598425	-2.19496469041631
C	-3.26527795244514	7.37070083768933	-1.33862004488168
H	-1.24186515304470	5.11016612984065	0.22045570967779
C	-2.28624970177570	6.84788837500657	-0.48053740112418
C	-5.80694520896441	2.15577716128969	-1.93712924282485
C	-1.98247141587006	5.49271628863461	-0.47073824147157
C	-2.64707689535383	4.59652702080864	-1.33325788834787
H	-1.76290880074225	7.51398656907254	0.20184019928125
H	-4.14180670014218	4.46201037243234	-2.88635869771284
H	-4.68320818897558	6.86082822760616	-2.87781094683235
C	-3.59831119692824	8.84050696671093	-1.32957065232513
C	-4.51453078736416	-2.24314741792172	-1.84186228122211
C	-4.16901398123670	-0.91327801785160	-1.62183394185329

C	-2.84272620921965	-0.47939989282834	-1.79270612507734
H	-1.46549587381696	-3.45675361474546	-2.73204676315038
C	-1.87784270978626	-1.41883664300392	-2.20563411746438
H	-3.81426504814330	-4.20723764016117	-2.39403938754861
C	-2.22621140032688	-2.74664013136177	-2.42151844675703
C	-3.54650918285328	-3.16657451218540	-2.23752574986324
H	-0.85490181435149	-1.09887809900423	-2.37267522687665
H	-5.54010514831242	-2.56175281825368	-1.68040599416052
H	-4.91838213246660	-0.21392342581745	-1.27812796447527
N	-1.19216072623441	1.17931797066400	-1.09661763616170
C	-2.43804061042097	0.89373059836233	-1.51372992733009
C	-7.21508591576042	2.04168075691163	-1.90104028920364
C	-1.09196823184731	2.56503986973065	-0.97522275162405
C	-3.21032121483407	2.10735256657181	-1.63514271605040
C	-2.34418840170980	3.17526888214917	-1.33529261944215
C	-8.03196714349008	3.15282213984927	-1.61698370657792
C	-4.58962778506482	2.16134354793872	-1.86206706111833
H	-11.08844559072089	1.64056896759018	-1.64925427029537
C	10.25893514030666	4.26062450548604	-0.45899479144332
H	-7.20259035412498	-0.07708365638901	-2.32220519042771
C	9.79398921953251	0.10465096411738	2.29870580326987
C	-7.82114066413828	0.78512258996092	-2.10171013731989
C	-9.20166247633105	0.65328291577820	-2.00718119154650
C	-10.01368223012645	1.75212393652602	-1.72486435446889
H	-7.57631820059611	4.12351032322637	-1.45635904565505
C	-9.41212391880938	2.99651678554845	-1.53407341690770
C	-1.99518241200297	-5.47790362545563	0.37605312644750
C	-2.25832815556564	-6.84512381081543	0.39346040723632
C	-3.18952366855025	-7.37398578421277	1.29024631087199
H	-4.10458996947528	-4.50246895836363	2.86448174384319
C	-3.85828464368971	-6.52180047073709	2.17347733847987
C	-5.89658569381479	-2.25838335152844	1.76926231314662
C	-3.59768803945664	-5.15483010668052	2.16093186881294
C	-2.65837965771013	-4.60865549731101	1.26449726664552

H	-4.57963580181444	-6.92502383394851	2.87878211057035
H	-1.28826793318312	-5.06865307066055	-0.33521953652274
H	-1.74054599798374	-7.49930879809053	-0.30255526826615
H	-3.39485342614691	-8.44068014368720	1.29971810930682
C	-2.55963340221551	2.78374200111929	2.16359147137945
C	-2.13023233804683	1.47209833357896	2.00423754843903
C	-3.02644112196068	0.46284792008156	1.60641444441089
H	-5.83049837265951	2.38683246088857	1.36417560177818
C	-4.36967938757642	0.82458466465563	1.39203990755501
C	-4.36127563566304	4.56812488743019	2.08921272919445
C	-4.79079014537005	2.13588347210805	1.55860942917328
C	-3.89618156724446	3.14420679469957	1.94137808208827
H	-5.07572532657651	0.07816837971412	1.05627643056593
H	-1.84487858286268	3.54495193173082	2.46530194976096
H	-1.09363911287267	1.22382665181770	2.20795226418410
N	-1.28296872103077	-1.15847875537789	1.03816471866350
C	-2.55476526577191	-0.89725891002635	1.39064975667635
C	9.23735165251647	-1.50667818406132	-1.48296281266659
C	-1.14112691681543	-2.54313785999954	0.94578365007700
C	-3.29668734881394	-2.13340277085815	1.50769692468567
C	-2.39144151213086	-3.17787830060387	1.25996306930554
N	-0.03157422042396	-3.20559963988440	0.65662552243607
C	-4.67910611270023	-2.23249390222736	1.70028795691573
H	-11.18679846938746	-1.80380732904570	1.54202701565445
C	-10.34445938017106	-4.33615829574985	1.05508127848884
H	-7.31105755635710	-0.04538565142601	2.16414192328501
C	-9.94459647974004	0.54248230761086	2.13175798119156
C	-7.92346371835818	-0.91398497963528	1.95242106824090
C	-9.30590771875624	-0.79639729128074	1.87615507431739
C	-10.10994846937297	-1.90409426804155	1.60463539755339
H	-7.65226538863945	-4.25138981629569	1.31060023819299
C	-9.49823375899443	-3.14237010936203	1.40860169550259
C	-7.30697726186250	-2.16482563520809	1.74568868429598
C	-8.11536921417660	-3.28486995267961	1.47482768048841

C	3.51512209573752	-5.59910772884492	-0.00611865791823
C	3.77237078884937	-6.86740701651666	0.50578697058368
C	3.11337683132444	-7.30895889759912	1.65660492882148
H	1.23957790205773	-4.54159667384558	2.29066191501561
C	2.19550278244419	-6.46890234342100	2.29128227290020
C	5.82546661427828	-2.78979076920284	-0.78577814720804
C	1.93566948382374	-5.19857696418965	1.78371479552567
C	2.58882889586065	-4.74345927639308	0.62145458402686
H	1.68567286889946	-6.80092169022559	3.19147134292752
H	4.01450606673410	-5.27131213095306	-0.91204640893029
H	4.48343423836149	-7.51632245983313	0.00210380341063
H	3.31621525057686	-8.29850268902180	2.05630920498253
C	4.74461360545626	1.36105968686413	-2.23062265648535
C	4.31508978964564	0.21836983713314	-1.57169787761349
C	2.96924534865571	-0.18936543438234	-1.63190649196209
H	1.80693995042055	2.33055382209763	-3.61448023063640
C	2.07870982569465	0.59444920069626	-2.38852360667720
C	4.33095338050342	3.40170053774366	-3.66104448795038
C	2.51698817491241	1.73998635484763	-3.04141127834988
C	3.85618944414382	2.14916298960083	-2.97433571516857
H	1.03972498079205	0.29520838138759	-2.48020322895503
H	5.78622877930685	1.66061867470406	-2.14669914468974
H	5.01666901220798	-0.34481255227897	-0.97246469679611
N	1.22232576236990	-1.46118357901493	-0.47899368045772
C	2.49220192992975	-1.36088038034687	-0.91144730654455
C	7.23637236837545	-2.71004066518346	-0.82504051277529
C	1.07832499579193	-2.70610094111008	0.13490088778972
C	3.23016297728925	-2.55306241614540	-0.55503491285050
C	2.32510335896826	-3.41864322497074	0.07944636188895
C	7.85262337487370	-1.62535686071727	-1.48100597171330
C	4.60948182269945	-2.72811308930290	-0.71252592160612
H	11.12170736670797	-2.35199974829653	-0.85220745568864
C	9.87560719141965	-0.35719334681046	-2.21586531673754
H	7.58335936325709	-4.50441975532659	0.32517668708399

C	10.28779225651232	-4.52393869205439	0.53084333744017
C	8.04646465715054	-3.66821164726640	-0.18610323156594
C	9.43062449334022	-3.53021839328857	-0.20675801487867
C	10.04288910887570	-2.45356710858410	-0.85003033433843
H	7.23921983951670	-0.88250700483741	-1.97710272100249
F	-11.60662546554454	-4.24167624295605	1.54817423524499
F	-9.82003254410365	-5.49957945079818	1.52246663143722
F	-10.46877693626656	-4.48607418326225	-0.29779157457744
F	-9.13748771518524	1.57616224369162	1.77472772948220
F	-10.23726892490461	0.72198326777374	3.45459962358730
F	-11.11334949745097	0.69674907160503	1.45669033087748
F	-11.01827538802043	-0.83928969584557	-1.62278529295736
F	-9.02861443691543	-1.71570615793928	-1.84882922444135
F	-10.06831946003379	-0.90235849710291	-3.58602699449543
F	-11.53366805346745	4.07343265746132	-1.64550079561740
F	-9.75983332831392	5.34964002607651	-1.65086945324826
F	-10.37131288404016	4.33693253995604	0.18240632191429
F	11.05472822112104	0.02226814003766	-1.65808857779088
F	9.07685805182649	0.74193378665223	-2.24869746623161
F	10.14912521122084	-0.67305758084018	-3.51714865781762
F	10.57187388881084	-4.10354236600337	1.79999213872283
F	11.48452123914963	-4.72750541871939	-0.08112832292007
F	9.69111177528895	-5.73763438912832	0.65232922895871
F	8.98965500151150	-0.99158435837231	2.27891725647183
F	10.03608967348977	0.37879117330846	3.61559382165425
F	10.98372494168972	-0.26322018811669	1.75794841941165
F	10.52788172958178	3.81222244961509	-1.72159618695457
F	11.46343349484003	4.45617965469345	0.14117021637015
F	9.68118647552477	5.48131219405516	-0.59901319644231
H	-5.21199024451562	4.63201166900651	2.77727727737388
H	-4.69352217446403	4.96516992831049	1.12274383240429
H	-3.55917594176033	5.20967667175403	2.46211630123443
H	3.52955829323129	3.86096125897815	-4.24485801322403
H	5.17335281292774	3.18609976821376	-4.32832246354679

H	4.67877274682810	4.13478845979498	-2.92374443603333
H	3.04295796188116	8.86822794277281	-3.01757387906810
H	4.70034805819036	8.69680796523268	-2.40251060506674
H	3.46166874280609	9.43718344812535	-1.38878905144534
H	-4.33818178666612	9.08127064712357	-2.09772937681007
H	-2.70251528759396	9.44668560984310	-1.50755301738497
H	-4.00345722257342	9.14255738662998	-0.35618001406377
